# Small RNAs in metastatic and non-metastatic oral squamous cell carcinoma

**DOI:** 10.1186/s12920-015-0102-4

**Published:** 2015-06-24

**Authors:** Patricia Severino, Liliane Santana Oliveira, Flávia Maziero Andreghetto, Natalia Torres, Otávio Curioni, Patricia Maluf Cury, Tatiana Natasha Toporcov, Alexandre Rossi Paschoal, Alan Mitchell Durham

**Affiliations:** Albert Einstein Research and Education Institute, Hospital Israelita Albert Einstein, Sao Paulo, SP Brazil; Hospital Heliopolis, Departamento de Cirurgia e Otorrinolaringologia, Sao Paulo, SP Brazil; Faculdade Ceres, Sao Jose do Rio Preto, SP Brazil; Departamento de Epidemiologia, Faculdade de Saúde Pública, University of Sao Paulo, Sao Paulo, SP Brazil; Federal University of Technology, Parana, Brazil; Instituto de Matemática e Estatística, University of Sao Paulo, Sao Paulo, SP Brazil

## Abstract

**Background:**

Small non-coding regulatory RNAs control cellular functions at the transcriptional and post-transcriptional levels. Oral squamous cell carcinoma is among the leading cancers in the world and the presence of cervical lymph node metastases is currently its strongest prognostic factor. In this work we aimed at finding small RNAs expressed in oral squamous cell carcinoma that could be associated with the presence of lymph node metastasis.

**Methods:**

Small RNA libraries from metastatic and non-metastatic oral squamous cell carcinomas were sequenced for the identification and quantification of known small RNAs. Selected markers were validated in plasma samples. Additionally, we used *in silico* analysis to investigate possible new molecules, not previously described, involved in the metastatic process.

**Results:**

Global expression patterns were not associated with cervical metastases. MiR-21, miR-203 and miR-205 were highly expressed throughout samples, in agreement with their role in epithelial cell biology, but disagreeing with studies correlating these molecules with cancer invasion. Eighteen microRNAs, but no other small RNA class, varied consistently between metastatic and non-metastatic samples. Nine of these microRNAs had been previously detected in human plasma, eight of which presented consistent results between tissue and plasma samples. MiR-31 and miR-130b, known to inhibit several steps in the metastatic process, were over-expressed in non-metastatic samples and the expression of miR-130b was confirmed in plasma of patients showing no metastasis. MiR-181 and miR-296 were detected in metastatic tumors and the expression of miR-296 was confirmed in plasma of patients presenting metastasis. A novel microRNA-like molecule was also associated with non-metastatic samples, potentially targeting cell-signaling mechanisms.

**Conclusions:**

We corroborate literature data on the role of small RNAs in cancer metastasis and suggest the detection of microRNAs as a tool that may assist in the evaluation of oral squamous cell carcinoma metastatic potential.

**Electronic supplementary material:**

The online version of this article (doi:10.1186/s12920-015-0102-4) contains supplementary material, which is available to authorized users.

## Background

Small noncoding RNAs are regulatory molecules that have recently emerged as important players in several aspects of cellular biology. They are approximately 18 to 30 nucleotides in length and act mostly through the inactivation of complementary sequences. MicroRNAs (miRNAs) and PIWI interacting RNAs (piRNA), for instance, are involved in sequence-specific and chromatin-dependent gene silencing [1]. A variety of small RNAs have been identified to date and the list is continuously growing, partly due to the advent of new sequencing technologies [2, 3].

Among these molecules, miRNAs have been extensively studied. MiRNAs reduce mRNA stability and/or translation due to full or partial sequence complementarity within target mRNAs. They are transcribed as large pri-miRNA, which are then folded into stem-loop structures, and transported to the cytoplasm where they undergo additional processing generating a double-stranded RNA [4, 5]. In general, one of the two complementary RNA molecules will integrate the RNA-induced silencing complex (RISC) and interact with miRNA complementary sites within target transcripts [6]. Initially described by Ambros and colleagues in the model organism *Caenorhabditis elegans* [7], miRNAs have been shown to modulate a broad range of molecular processes involved in tissue homeostasis and, ultimately, in the pathogenesis of human diseases, including cancer [8, 9]. Despite consensus on the role of miRNAs in cancer development, the part they play in the metastatic cascade is not well defined. A number of studies have already addressed miRNAs that may regulate this multistep process (for a review see [10]), and the use of miRNAs expression profile for the discrimination between metastasized and non-metastasized tumors can be exemplified by a recent publication in which the expression level of two miRNAs discriminated between nonmetastatic and metastatic testicular cancer [11].

More recently, genome-wide analysis following The Cancer Genome Atlas [12], showed that changes in the expression levels of other small non-coding RNAs are also associated with cancer, with strong correlations between RNA abundance and disease status [13]. Altogether, these results indicate the possible application of such molecules as disease markers.

Head and neck squamous cell carcinomas (HNSCC) arise from epithelial cells in the lining of the upper aerodigestive tract, comprising the nasal cavity and paranasal sinuses, the nasopharynx, larynx, pharynx, the oral cavity and the oropharynx. HNSCC is one of the leading cancer types by incidence worldwide, with approximately 500,000 new cases a year worldwide and a five-year survival rate of about 40-50 % [14]. Tumors usually develop in men over the age of 60 years and tobacco and alcohol consumption are the most important risk factors [15, 16].

Oral squamous cell carcinoma (OSCC) is a deadly disease and, when grouped with pharyngeal cancer, it is the sixth most common cancer in the world [17]. This tumor is particularly risky because in its early stages it progresses without producing pain or symptoms that might be readily recognized by the patient. It is usually discovered when the cancer has metastasized to the lymph nodes of the neck and at this stage its prognosis is significantly worse than when it is caught in a localized intra oral area. In fact, the presence of cervical lymph node metastasis is considered the most significant prognostic indicator of survival and disease recurrence in patients with HNSCC, with an approximate decrease in 50 % in 5-year survival rate [18]. Thus, markers that could help in the identification of the metastatic phenotype could be of use to the clinical setting.

In this work we aimed at finding small RNAs expressed in OSCC that could be associated with the presence of lymph node metastasis. We selected patients with tumors at early stage of development (T1 or T2) with neck metastases, and later stage tumors (T3 or T4) with no neck lymph node metastases for high-throughput sequencing aiming at the quantification of small RNAs. Selected markers were validated in plasma collected from HNSCC patients before surgery and in additional tumor samples at various pathological stages. The identification of a biomarker in plasma is of great use to the clinical practice due to the minimally invasive characteristic of this kind of assay. Additionally, we used *in silico* analysis to investigate possible new molecules, not previously described, involved in the metastatic process.

## Results and discussion

### Highly expressed MiRNAs are common to OSCC samples despite TNM staging

The small RNA fraction of 18 OSCC was sequenced. Clinical and pathological data associated with the samples are described in Table 1. Two groups of samples were sequenced: small tumors (T1 and T2) presenting lymph node metastasis at the time of diagnosis and larger tumors (T3 and T4), which were metastasis-free at the time of diagnosis. The reasoning behind dividing samples in these two groups was to minimize biological variation within the groups and, possibly, to be able to select stronger markers linked to the metastatic phenotype due to their earlier presence in the tumor progression (i.e., in T1/T2N+ samples) or maybe a protective role due to their presence in non-metastatic larger tumors.

**Table 1:** Clinical data on patients selected for miRNA sequencing and for miRNA identification in plasma

Patient	Site	Gender	Age (yr)	Pathologic stage	Sample	Tissue type	Experiment
p0040	OC-T	Male	48	T4N0M0	microRNA	Tumor	A
p0151	OC-T	Male	47	T3N0M0	microRNA	Tumor	A
p0291	OC-T	Male	55	T4N0M0	Total RNA	Tumor	A
p0340	OC-FOM	Male	51	T4N0M0	microRNA	Tumor	A
p0374	OC-T / OC-FOM	Male	56	T1N2bM0	microRNA	Tumor	A
p0397	OC-FOM	Male	59	T1N3M0	microRNA	Tumor	A
p0418	OC-T	Female	64	T4N0M0	microRNA	Tumor	A
p1022	OC-T	Male	44	T3N0M0	Total RNA	Tumor	A
p1125	OC-T	Male	53	T3N0M0	microRNA	Tumor	A
p1231	OC-FOM	Male	56	T1N2bM0	microRNA	Tumor	A
p1381	OC-FOM	Male	70	T1N2bM0	microRNA	Tumor	A
p1642	OC-T	Male	80	T1N1M0	microRNA	Tumor	A
p0012	OC-FOM	Male	50	T1N1M0	Total RNA	Tumor	A
p0280	OC-FOM	Male	52	T1N2bM0	Total RNA	Tumor	A
p0441	OC-T	Male	54	T1N1M0	microRNA	Tumor	A
p0486	OC-T	Male	56	T3N0M0	microRNA	Tumor	A
p0652	OC-FOM	Male	63	T1N1M0	microRNA	Tumor	A
p0677	OC-FOM	Male	58	T1N2bM0	microRNA	Tumor	A
p1_0250	OP-BT	Male	52	T4aN2aM0	Total RNA	Tumor	B
p1_0301	OP-BT	Male	51	T4N0M0	Total RNA	Tumor	B
p1_0076	OC-T	Male	63	T2N2M0	Total RNA	Tumor	B
p1_0151	OC-T	Male	65	T2N2cM0	Total RNA	Tumor	B
p2_0072	OC-T	Male	53	T3N0M0	Total RNA	Tumor	B
p2_0021	OC-FOM	Male	63	T3N0M0	Total RNA	Tumor	B
p2_0048	OC-FOM	Male	52	T2N2bM0	Total RNA	Tumor	B
p1_0273	OP-BT	Male	68	T1N3M0	Total RNA	Tumor	B
p2_0057	OC-FOM	Male	59	T3N0M0	Total RNA	Tumor	B
p0057	OC-T	Male	57	T4N1M0	microRNA	Tumor	B
p0273	OC-T	Male	56	T4N1M0	microRNA	Tumor	B
p0015	OC-FOM	Male	57	T4N1M0	microRNA	Tumor	B
p0113	OC-FOM	Male	67	T4N0M0	microRNA	Tumor	B
p0166	OC-T	Male	54	T4N0M0	microRNA	Tumor	B
p0335	OC-T	Female	85	T4N0M0	microRNA	Tumor	B
p1_0046	OC-T	Male	54	T4aN2cM0	microRNA	Plasma	C
p1_0105	OC-T	Female	80	T4aN2cM0	microRNA	Plasma	C
p1_0134	OC-T	Male	57	T3N2bM0	microRNA	Plasma	C
p1_0149	OC-T	Male	60	T1N0M0	microRNA	Plasma	C
p1_0151	OC-T	Male	65	T2N2cM0	microRNA	Plasma	C
p1_0328	OC-T	Male	65	T2N0M0	microRNA	Plasma	C
p2_0020	OC-T	Male	58	T2N0M0	microRNA	Plasma	C
p2_0025	OC-T	Male	58	T2N1M0	microRNA	Plasma	C
p2_0068	OC-T	Male	67	T1N0M0	microRNA	Plasma	C
p2_0072	OC-T	Male	53	T3N0M0	microRNA	Plasma	C
p1_0025	OC-FOM	Male	61	T4aN2cM0	microRNA	Plasma	C
p1_0049	OC-FOM	Male	70	T4aN1M0	microRNA	Plasma	C
p1_0061	OC-FOM	Male	50	T4aN2cM0	microRNA	Plasma	C
p1_0111	OC-FOM	Male	55	T1N3M1	microRNA	Plasma	C
p1_0115	OC-FOM	Male	56	T4aN2cM1	microRNA	Plasma	C
p1_0119	OC-FOM	Male	50	T4bN3M0	microRNA	Plasma	C
p1_0220	OC-FOM	Male	50	T1N0M0	microRNA	Plasma	C
p1_0236	OC-FOM	Male	53	T4aN2bM0	microRNA	Plasma	C
p1_0248	OC-FOM	Male	55	T2N2aM0	microRNA	Plasma	C
p1_0308	OC-FOM	Male	50	T4aN0M0	microRNA	Plasma	C
p1_0323	OC-FOM	Male	50	T4aN2cM0	microRNA	Plasma	C
p1_0335	OC-FOM	Female	59	T2N0M0	microRNA	Plasma	C
p2_0021	OC-FOM	Male	63	T3N0M0	microRNA	Plasma	C
p2_0031	OC-FOM	Male	62	T1N2bM0	microRNA	Plasma	C
p2_0035	OC-FOM	Male	57	T1N0M0	microRNA	Plasma	C
p2_0041	OC-FOM	Male	64	T4aN0M0	microRNA	Plasma	C
p2_0048	OC-FOM	Male	52	T2N2bM0	microRNA	Plasma	C
p2_0058	OC-FOM	Male	58	T4N2bM0	microRNA	Plasma	C
p2_0062	OC-FOM	Male	55	T1N0M0	microRNA	Plasma	C
p2_0095	OC-FOM	Male	52	T3N2bM0	microRNA	Plasma	C

OC-T: Oral Cavity – Tongue, OC-FOM: Oral Cavity - Floor of the Mouth. Pathological Stage describes the metastatic status of the samples, with N0 meaning no lymph node metastasis at the time of diagnosis and N > 0 indicating the presence of lymph node metastasis. Experiment description: A- tumor tissue samples used for small RNA sequencing; B – tumor tissue sample used for miRNA expression validation by real-time PCR; C - plasma samples used for miRNA expression detection by real-time PCR

**Fig. 1:**
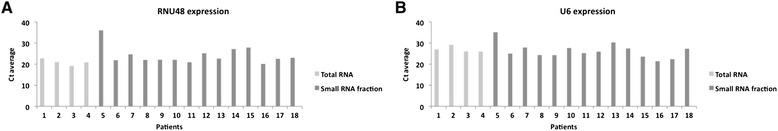
Expression levels of RNU48 and U6 in miRNA samples and total RNA samples used as input for the sequencing protocol. **a**: Expression levels of RNU48; **b**: Expression levels of U6.

Template RNA for library sequencing was either total RNA or the small-RNA fraction, according to the availability in our specimen repository at the time of the experiment. In order to evaluate differences in the small-RNA fraction associated with the kind of RNA template, we assessed the expression levels of two endogenous controls (RNU48 and U6) in each sample. Figure 1 shows that there were no significant differences in the expression levels of these two molecules associated with total RNA or small-RNA fractions.

**Fig. 2:**
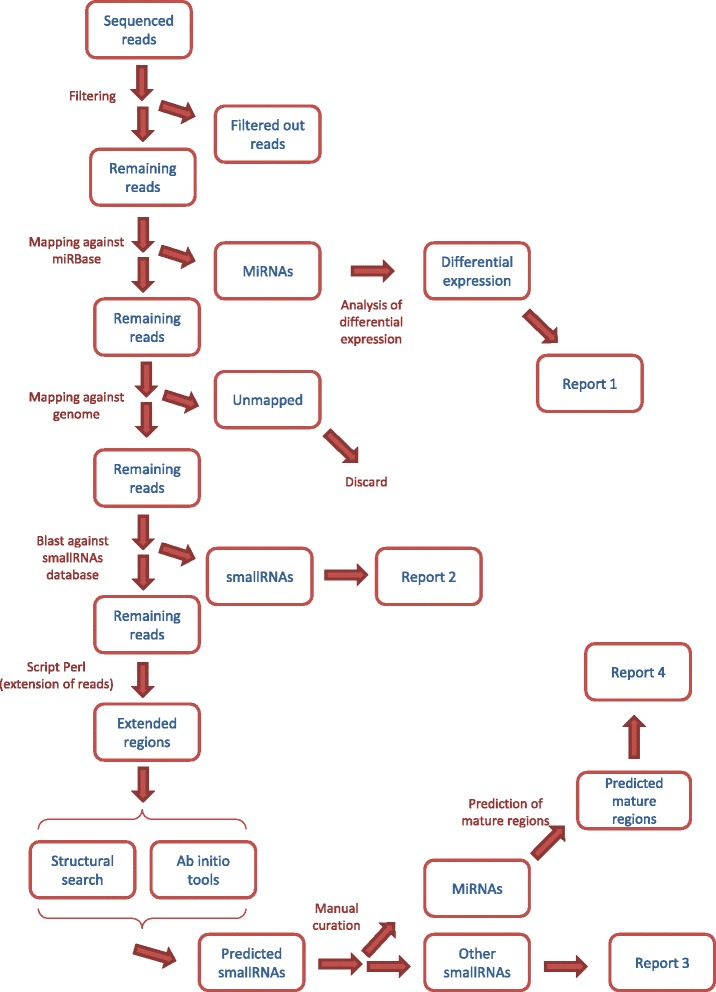
Workflow for sequencing data Analysis

**Fig. 3:**
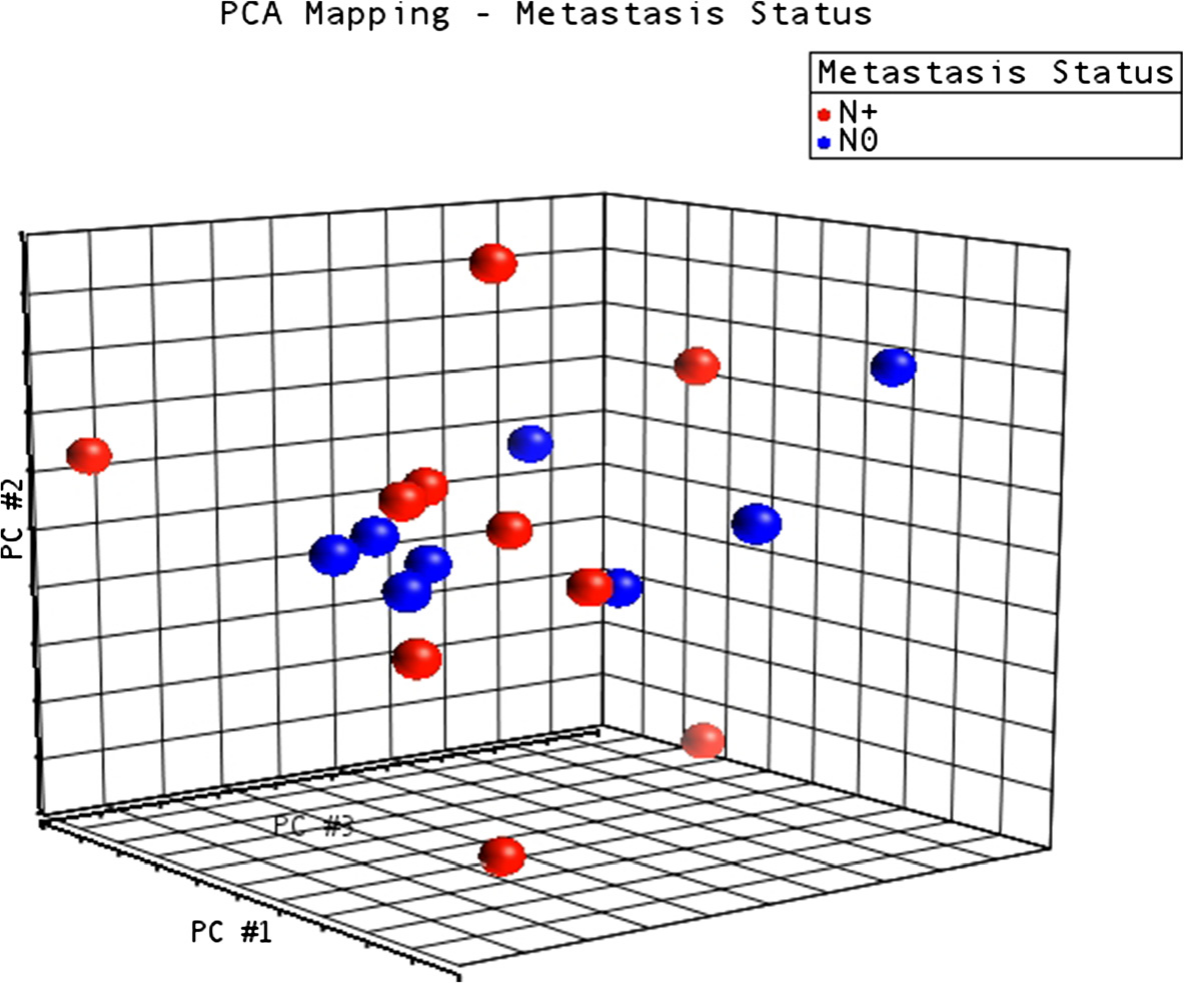
PCA plot depicting global miRNA RNA expression in OSCC samples

The total number of sequenced and mapped reads is reported in Additional file 1. Our analysis of the sequencing data followed the workflow depicted in Fig. 2. In total, 984 mature miRNAs were identified when all samples were considered, regardless of the sequenced arm (i.e., 3p or 5p) (Additional files 2 and 3). Concerning global expression levels of miRNAs, no association with TNM staging was observed (Fig. 3). However, metastatic tumors (N+) seemed to be more heterogeneous in terms of the expression of these small RNAs than non-metastatic (N0) samples, as depicted by the dispersion of samples in the PCA plot.

The 10 most expressed miRNAs corresponded to at least 50 % of the total number of expressed miRNAs (Additional files 4 and 5). MiR-21 and miR-205 were the most expressed molecules in almost all samples. The relative expression of miR-205, known to be mostly expressed in squamous cells, has been previously addressed as a metastasis marker for HNSCC [19]. In our dataset, however, the molecule is highly but ubiquitously expressed and cannot, therefore, be considered a metastasis marker.

Similarly, miR-21 has been previously associated with metastatic tumors and a few mechanistic explanations have been proposed: in hepatocarcinoma it was reported to target the tumor suppressor gene RHOB [20], in non-small cell lung cancer it promoted metastasis by targeting PTEN, and in breast cancer miR-21 was associated with low levels of TIMP-3 in metastatic samples [21]. In squamous cell carcinomas, increased expression of miR-21 was observed in human tumors characterized by p53 mutations and distant metastasis, and the augmented expression of miR-21, mediated by active mTOR and Stat3 signaling, conferred increased invasive properties to mouse keratinocytes in vitro and in vivo [22]. Recently it was associated with cancer invasion via the Wnt/β-Catenin pathway in a study using an oral squamous cell carcinoma cell line [23]. In the set of samples analyzed here we did not identify differential expression of miR-21 between metastatic and non-metastatic tumors, but rather miR-21 was the most or the second most expressed molecule in almost every sample. In a previous report we showed that miR-21 and miR-205 were both highly expressed in squamous cell carcinoma samples, cancer-free surgical margins as well as in a cell line and normal oral keratinocytes, with no marked differences in expression levels [24].

MiR-203, a marker of differentiated human keratinocytes, known to regulate keratinocyte proliferation and regulation in adult epidermis [25, 26] and implicated in cancer progression and metastasis [27, 28], was also abundantly expressed in our samples.

Despite the role of miR-21, miR-203 and miR-205 in cellular processes associated with cancer metastasis, clearly demonstrated when each one is studied independently, their concurrent importance in keratinocyte physiology constitutes a drawback when studying them in regard to proliferation and invasion in the context of squamous cell carcinomas.

### miRNAs as metastasis markers: evidences from tissue and plasma samples

For the identification of miRNAs possibly involved in the metastatic process we selected reads counted at least 10 times per sequenced sample and used the EdgeR Bioconductor software package for data normalization and differential expression analysis between metastatic (N+) and non-metastatic (N0) samples. Table 2 lists miRNAs that presented at least a 2-fold difference in expression between the two groups and which were regulated in at least half the samples from each group.

**Table 2:** Differential miRNA expression between non-metastatic and metastatic OSCC samples

	Fold change (N0/N+)	P value
**hsa-mir-31-5p**	3.33	2.02E-002
**hsa-mir-130b-5p**	3.04	3.14E-002
hsa-mir-301a-3p	2.29	1.06E-001
hsa-mir-1301	−2.07	1.89E-001
hsa-mir-551b-3p	−2.10	1.60E-001
hsa-mir-345-5p	−2.22	1.37E-001
hsa-mir-30a-5p	−2.31	1.25E-001
hsa-mir-326	−2.32	1.18E-001
hsa-mir-769-5p	−2.35	1.13E-001
hsa-mir-139-5p	−2.40	1.02E-001
hsa-mir-218-5p	−2.72	6.69E-002
hsa-mir-106a-5p	−2.81	8.02E-002
**hsa-mir-335-5p**	−3.56	2.16E-002
**hsa-mir-296-5p**	−3.58	2.21E-002
**hsa-mir-20b-5p**	−3.88	1.54E-002
**hsa-mir-23c**	−4.98	4.83E-003
**hsa-mir-1277-3p**	−9.82	1.80E-004
**hsa-mir-181d-5p**	−10.01	1.66E-004

Positive Fold Change: higher expression in non-metastasized OSCC; negative Fold Change: higher expression in metastasized OSCC. p-values were adjusted using the False Discovery Rate (FDR) correction and adjusted p values < 0.05 are in bold in order to highlight statistical significance. The suffix 3p/5p indicate which of the two arms of the miRNA was considered in the analysis, being either exclusively expressed or predominantly expressed in the dataset (Additional files 2 and 3 present detailed information on the expression levels of each arm)

Two microRNAs were significantly more expressed in N0 when compared to N+ samples: miR-31 and miR-130b. In agreement with our data, miR-31 was previously described as a potent inhibitor of breast cancer metastasis [29], and was also shown to impair breast cancer-derived lung metastases through a specific mechanism targeting cell cycle arrest and apoptosis [30]. Higher levels of miR-31 expression in HNSCC when compared to cancer-free tissue have been reported [24, 31] but a potential role for miR-31 in HNSCC metastasis has never been proposed.

MiR-130b was also found up regulated in HNSCC when compared to cancer-free tissues [32, 24] and it is involved in immortalization of normal oral keratinocytes [33]. Its possible role in metastasis was recently demonstrated when its over-expression decreased migration and invasion in colorectal cancer cells [34]. Our data, thus, corroborates the reported involvement of miR-31 and miR-130b in metastasis and supports the their possible use as molecular marker for non-metastatic cancer or as therapeutic molecules. Results for miR-130b and for miR-31 were confirmed in an additional set of 15 samples corresponding to 8 metastatic and 7 non-metastatic tumors of different pathological stages (Table 1). MiR-130b and miR-31 presented 2.27 and 1.97 fold-change differences in gene expression levels, respectively, when comparing N0 and N+ samples (Additional file 6).

Six miRNAs were found to be up regulated in metastatic OSCC with statistic significance (*p* < 0.05). The most expressed molecule in N+ samples, miR-181, has been previously associated with metastasis and, in fact, it has been considered as a marker for lymph-node metastasis in OSCC [35]. Another miRNA involved in metastatic processes is miR-296, possibly through targeting ICAM-1 [36]. Corroborating the sequencing results, in the additional set of 15 tumor samples miR-296 was over-expressed in N+ samples (3.3 fold change, Additional file 6).

Besides the above-mentioned topics, little is known about the involvement of the miRNAs identified here and the metastatic cascade. Despite important results when miRNAs are studied individually, considering the complexity of miRNA regulatory networks, numerous additional studies are still needed for a better understanding of the whole system.

**Fig. 4:**
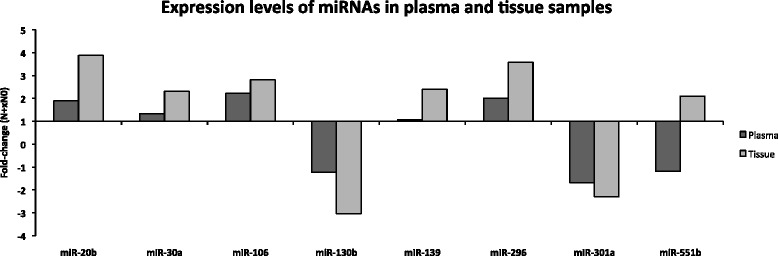
Expression levels in plasma of miRNAs differentially expressed between N+ and N0 tissue samples according to sequencing results. MiRNA selected for this evaluation were those that had been previously detected in human plasma and showed differential expression between metastatic and non-metastatic OSCC samples

However, for clinical application, a biomarker may become useful without a full comprehension of its mechanistic role in the pathophysiology of a disease, in particular if the biomarker can be readily assessed in body fluids such as blood or saliva. The stability of miRNAs in plasma has been demonstrated, including for OSCC [37]. From our list of 18 miRNAs showing at least a 2-fold difference in expression levels between N+ and N0 samples and consistent expression in each of the two groups, 9 had been detected in human plasma during large scale screening studies [38, 39]. Thus, we chose to evaluate their expression levels in plasma using an additional set of 30 patients (Table 1). A comparison between read counts from the sequencing approach and real-time expression levels is not straight forward, but Fig. 4 shows that, except for miR-551b, up or down regulation considering N+ or N0 groups was consistent in tissue and plasma.

Due to the reported role of miR-31 in inhibiting metastasis, we addressed its expression in plasma despite the fact that it had not been previously detected in human plasma [38, 39]. As expected, we could not detect miR-31 in the additional set of 30 OSCC patients (data not shown).

Cell-free microRNAs detected in plasma are of clinical interest due to their possible application as non-invasive biomarkers and literature reports on this possible application is resonant (for a review see [40]). Despite the large number of articles addressing the issue, most results lack validation.

### Known small RNAs other than miRNA and their contribution to the metastatic phenotype

Small RNAs other that miRNAs may also be implicated in cancer progression/metastasis. Piwi-interacting RNA (piRNA) pathway, for instance, is known for its role in germ cell maintenance and is currently being studied in the context of cancer [41], and snoRNAs have also been shown to contribute to oncogenesis [42].

In order to search for small RNAs other than miRNAs in our dataset we used BLAST to match reads that did not correspond to sequences deposited in miRBase against a dataset containing sequences from public non-coding RNA databases. A total of 197 reads had good hits. Of these, 68 reads were associated with specific ncRNA categories, in particular: piRNA (Piwi-interacting RNA), snoRNA (small nucleolar RNA), snRNA (small nuclear ribonucleic acid), Y RNA, easRNA (exon-associated small RNA), rasRNA (repeat-associated small RNA) or pasRNA (promoter-associated small RNA) (Additional file 7). The remaining reads had hits associated with annotated sequences that did not specify the ncRNA type (most of the original reads were annotated using ab initio methods for detecting covariance) (Additional file 8).

**Fig. 5:**
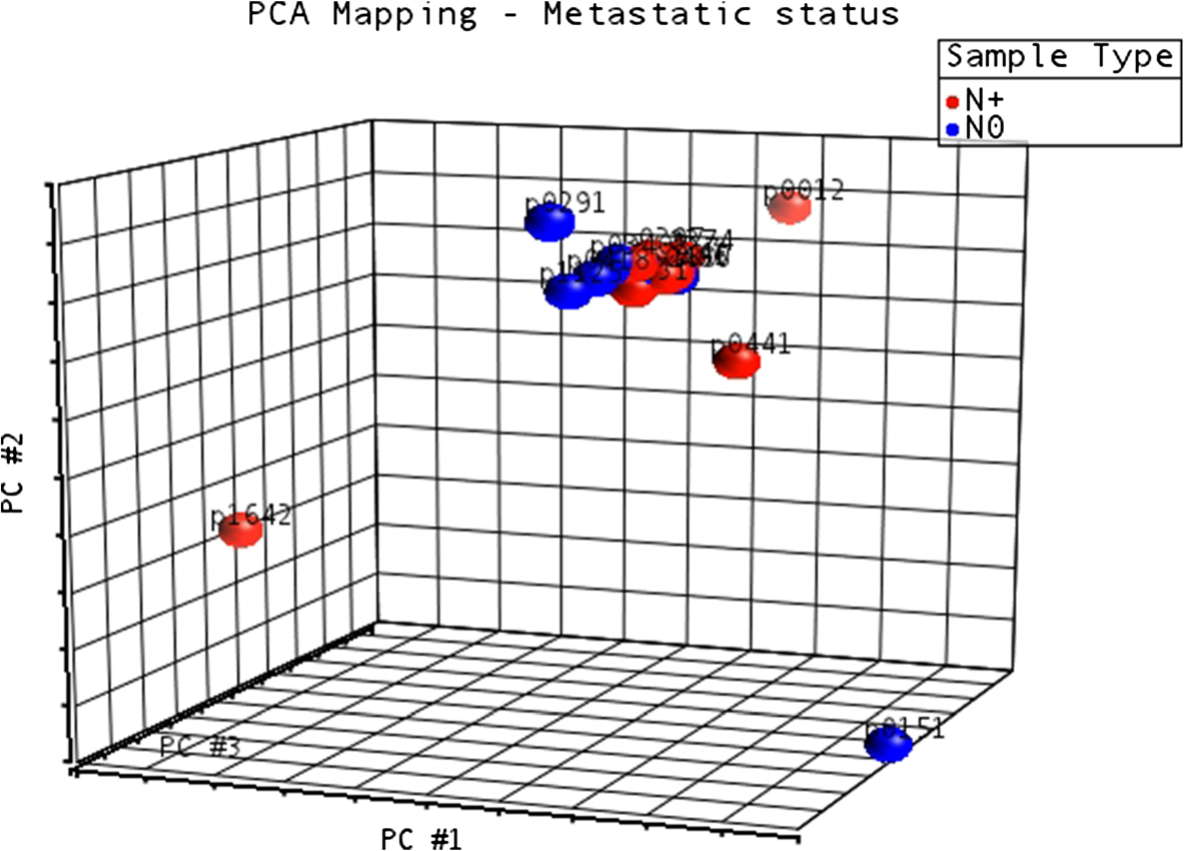
PCA plot depicting global small RNA expression in OSCC samples. For the clusterization of clinical samples based on the expression levels of small RNAs other that miRNAs we used Principal Components Analysis (PCA). All annotated molecules were included in this analysis. Results show that samples are very homogeneous concerning the expression of these small RNA molecules

The expression levels of these small RNAs are reported in Additional file 9. Figure 5 shows that their expression was mostly homogeneous in our dataset, with the exception of the non-metastatic sample p0151 and the metastatic sample p1642, both tongue-derived samples.

To our knowledge there is no published data on the expression of small RNAs other than miRNAs and HNSCC. We show that despite homogeneity in their global expression, small RNAs other than miRNAs are expressed and should exert a regulatory function associated with the cancer phenotype.

### Identification of a putative miRNA molecule

The utilization of new sequencing technologies allows, besides traditional analysis of gene expression analysis, the possibility of identifying new molecules possibly linked with a certain disease phenotype. In order to investigate new molecules in our dataset we selected reads that showed no positive match with miRNAs and other small RNAs but that mapped to the human genome and that presented differential expression between N0 and N+. In brief, we performed ab initio and structural search based on putative precursors sequences constructed from our sequenced reads, following the workflow depicted in Fig. 1.

Table 3 shows the 7 best candidates considering ab initio and structural search. There was variation in the prediction depending on the position of the read in the putative precursor since structural and ab initio classification searches are influenced by nucleotide sequence and the by the length of the sequence. Three reads had positive classification only for C/D snoRNA. Considering the reported specificity of snoReport (0.91 for the classification of C/D snoRNAs) and the high scores obtained by the candidates, these sequences were annotated as snoRNAs and were not further analysed.

**Table 3:** MiRNA-like molecules identified using structural search and ab initio prediction

Candidate number	Ext	Chr	Start Position	End Position	Pred	Length	SnoReport score	HMMiR likelihood ratio	Fold-change (N0/N+)
356	Both	chr4	102,540,761	102,541,036	miRNA	276	0	0.69	13.58
1731	Both	chr4	112,606,383	112,606,658	miRNA	276	0	0.7	56.18
1731	5'	chr4	112,606,478	112,606,658	miRNA	181	0	0.7	56.18
6039	Both	chr3	155,125,921	155,126,196	snoRNA C/D	276	0.99 (−)	0	48.87
6039	3'	chr3	155,125,921	155,126,101	snoRNA C/D	181	0.99 (−)	0	48.87
8442	Both	chr21	19,709,655	19,709,930	snoRNA C/D	276	0.99 (−)	0	9.21
12,375	5'	chr19	35,649,281	35,649,461	miRNA	181	0	0.7	12.57
15,215	3'	chr17	51,543,077	51,543,257	miRNA	181	0	0.67	12.94
16,666	Both	chr3	608,518	608,793	snoRNA C/D	276	0.96 (+)	0	−16.14
16,666	3'	chr3	608,518	608,698	snoRNA C/D	181	0.96 (+)	0	−16.14
19,713	Both	chr3	7,941,925	7,942,200	miRNA	276	0	0.69	−21.66
19,713	5'	chr3	7,942,020	7,942,200	miRNA	181	0	0.69	−21.66

Candidate number indicates the number of the read in our dataset. Extension (Ext) indicates which of the three genomic extractions the classification refers to the location of the 120 nucleotide extension performed on the original read (3’- 25 nt upstream, 120 nt downstream, 5’ – 120 nt usptream, 25 nt downstream, Both – 120 nt upstream, 120 nt downstream). Prediction (Pred) indicates the annotation attibuted to the candidate. Positive Fold Change: higher expression in non-metastasized OSCC; negative Fold Change: higher expression in metastasized OSCC

**Fig. 6:**
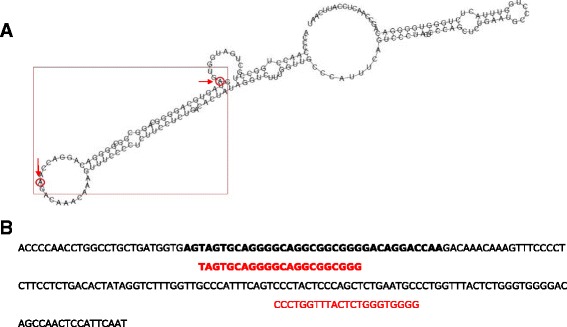
MiRNA-candidate structure and mature sequence prediction. **a**: The structure predicted using RNAfold contains the sequence read within the precursor stem (indicated by the arrows). This position suggests that the read could be processed into a mature miRNA; **b**: Prediction of a mature miRNA molecule within our predicted structure according to MatureBayes. The sequenced read is in black bold and the mature sequence predicted by MatureBayes is in bold red

**Fig. 7:**
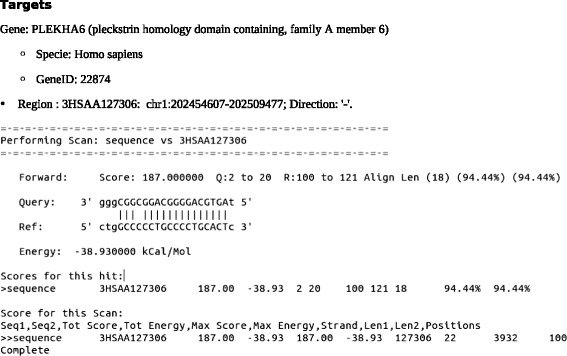
Putative miRNA-candidate mRNA target. Alignment between the predicted mature region of the miRNA-candidate and the 3’ UTR region of *PLEKHA6* (pleckstrin homology domain containing, family A member 6)

If any of the remaining reads corresponded to a mature miRNA, it would most likely map within the stem of the predicted structure. The only candidate that fulfilled this criterium was 12375 (Fig. 6a). Additionally, as a means to add to this structural prediction, we used MatureBayes to find a putative mature sequence within this structure, and the algorithm found one that mapped exactly within our sequenced read (Fig. 6b). A similarity search against 3’UTR sequences of the human genome allowed us to identify a possible target for this predicted mature sequence, *PLEKHA6* (pleckstrin homology domain containing, family A member 6) (Fig. 7). Candidate 12375 was mostly expressed in N0 samples, and could possibly target *PLEKHA6*. Although there are no current studies available for the role of PLEKHA6 protein, the pleckstrin homology domain occurs is a variety of protein involved in intracellular signaling [43].

The identification of new miRNA molecules is not obvious and several studies have published lists of novel molecules based solely on structural prediction. Despite the manually curated evidences shown here, we believe functional studies are necessary in order to confirm this assumption.

Noteworthy, the expression levels of the sequenced read belonging to this molecule as well as to the other predicted ones suggest a possible role for them in OSCC, regardless of the RNA class they may belong to.

## Conclusions

The possibility to evaluate the metastatic potential of OSCC is relevant to the clinical and molecular oncologist due to the possible asymptomatic development of such cancer in its early stages. Here we report the identification of small RNAs linked to the metastatic status of a group of OSCC, both in tissue and plasma samples. Given the diversity of roles of individual miRNAs during the metastasis cascade, including both promoting and suppressive effects, numerous studies are still necessary for a broad comprehension of their responsibility in this scenario. For other small RNA classes, perhaps contributing to the regulatory network, information is still very scarce.

We corroborate results on two metastasis inhibitors studied in other cancer types, miR-31 and miR-130b, and on two metastasis enhancers, miR-181 and miR-296, in the context of OSCC. We also demonstrate that other small RNA classes are expressed and should, therefore, be involved and contribute to the mestastatic phenotype of this disease.

## Methods

### Patients and samples

Eighteen patients with OSCC were selected for small RNA sequencing and 30 patients for the identification of circulating miRNAs in plasma. Table 1 shows the clinical and pathological profile of patients. Tumors were staged according to the Union for International Cancer Control (UICC)/American Joint Committee on Cancer (AJCC) staging classification system for HNSCC (7^th^ edition). All patients were smokers at the time of cancer diagnosis and had a history of chronic alcohol use. Primary tumor tissue samples were collected from patients submitted to surgical resection of primary tumor at Hospital das Clinicas and Hospital Heliopolis, in Sao Paulo, Brazil, and plasma samples were collected from patients before surgery for tumor resection at Hospital das Clinicas and Hospital Heliopolis. All patients provided written informed consent, and the research protocol was approved by review boards of the institutions involved and by the National Committee of Ethics in Research (CONEP 1763/05). Tumor samples were snap-frozen in liquid nitrogen immediately after surgery and stored. Analysis of hematoxylin and eosin-stained sections by the study pathologists confirmed at least 70 % of tumor cells in all OSCC samples.

### Total RNA and small RNA fraction isolation from tumor samples

RNA was prepared from OSCC tissue samples using AllPrep DNA/RNA/Protein Mini Kit (Qiagen) in compliance with the manufacturer’s protocol. RNA integrity and miRNA population concentration were assessed using the RNA 6000 Nano Assay kit and the Small RNA Assay kit, respectively, with Agilent 2100 Bioanalyzer according to the manufacturer's instructions (Agilent Technologies, Palo Alto, CA).

### Small RNA library construction and sequencing

Eighteen small RNA libraries were constructed, one for each OSCC sample: 10 samples presented lymph node metastasis at the time of diagnosis and 8 samples did not present metastasis. Small RNA library construction followed the SOLiD Total RNA-Seq Kit for Small RNA Libraries protocol (Ambion Inc., USA) and the SOLiD RNA Barcoding System (Ambion Inc., USA) was used for library multiplexing. One μg of total RNA was used as template. The SOLiD 5500 Genetic Analyser (Applied Biosystems, CA, USA) was used to generate 35 bp-long reads. Default parameters were used at all instances during sequencing. Sequencing was performed at GENIAL (Genome Investigation and Analysis Laboratory), CEFAP-USP (Centro de Facilidades de Apoio à Pesquisa da Universidade de São Paulo).

### Sequencing data analysis

For miRNA annotation we used the Small RNA Analysis Tool (RNA2MAP, part of LifeScope™ Genomic Analysis Solutions, Life Technologies) (LifeScopeTM Genomic Analysis Software 2.5.1 Applied Biosystems. 2012) [44] with the following parameters: three color-space mismatches within the 'seed sequence' (first 18 bases of the reads), and six color-space mismatches on the following positions of the 35 bp reads. Sequences matching tRNA, rRNA, DNA repeats and adaptor molecules were filtered out. The remaining reads were matched against the miRBase database, release 20 (http://www.mirbase.org/).

Alignments were restricted to 5 hits and, aiming to identify molecules most likely to represent a miRNA, multiple hits were only considered when representing different positions within of a single miRNA family. For instance, reads could map identically to hsa-mir-24-1 and hsa-mir-24-2 but for further analysis both such hits were counted as “hsa-mir-24” and only reads matching the mature miRNA sequence were counted as known miRNAs.

To visualize better the differential expression in clinical samples we clustered the candidates based on the global expression patterns. This clustering was performed using the Principal Components Analysis (PCA) implemented within Partek Genomics Suite (v6.6).

Due to concerns regarding public sharing of patient sequence dataset and the anonymity of patients, raw sequencing results are available upon request but, depending on the scope of the study, it will have to be submitted to the Ethics Committee approval.

### Differential gene expression analysis

For the analysis of small RNA expression levels between N+ and N0 samples we used the EdgeR Bioconductor software package [45]. In EdgeR, a Poisson model is used to account for biological and technical variability, and empirical Bayes methods are used to assess the degree of over dispersion across transcripts. For this analysis we included only reads with at least 10 counts per sample, and which presented the same direction in expression levels (either up or down regulated) across at least half the samples belonging to each group. Molecules that presented at least a 2-fold difference in expression between the two groups and which were regulated in at least half the samples from each group were considered differentially expressed. Additionally, differential expression was considered statistically significant when FDR (False Discovery Rate) corrected p value was < 0.05.

### Detection of circulating miRNAs in plasma

Plasma was separated from 5 ml of EDTA whole blood using a centrifugation step of 3600 rpm for 10 min. The miRNA population was isolated from 200 μl of plasma using the miRCURY RNA Isolation Kit – Biofluids (Exiqon), following the instructions provided in the manual. For the identification of miRNAs we used the Locked Nucleic Acid (LNA™)-based miRNA qPCR platform from Exiqon. Briefly, 4 μl of RNA were used for cDNA synthesis using MiRCURY LNA™ Universal RT kit microRNA PCR. The cDNA was diluted following the manufacturer’s protocol and real time PCR was carried out using specific, pre-defined microRNA primer pairs and the ExiLENT SYBR Green Master Mix (Exiqon), following the manufacturer’s protocol, using a ABI7500 instrument (Lifetechnologies). MiRNAs evaluated in plasma were those identified as differentially expressed between N+ and N0 tumors following small RNA sequencing in this study but which had been previously detected in plasma by a large scale study [38]: miR-20b (Exiqon 204755), miR-30a (Exiqon 205695), miR-31 (Exiqon 204236), miR-106a (Exiqon 204563), miR-130b (Exiqon 204317), miR-139 (Exiqon 205874), miR-296 (Exiqon 204436), miR-301 (Exiqon 204687), miR-335 (Exiqon 204151), miR-551b (Exiqon 204067). As a reference gene for data normalization we used miR-93 (Exiqon 204715), as suggested by the manufacturer and validated in our samples as stably expressed.

### Relative quantification of miRNA expression levels in tissue samples using Real Time-PCR

To validate the sequencing data, 3 miRNAs were subjected to quantitative Real Time-PCR using the Locked Nucleic Acid (LNA™)-based miRNA qPCR platform from Exiqon. Briefly, 5 ng of RNA were used for cDNA synthesis using MiRCURY LNA™ Universal RT kit microRNA PCR. Real time PCR was carried out using specific, pre-defined miRNA primer pairs and the ExiLENT SYBR Green Master Mix (Exiqon), following the manufacturer’s protocol and an ABI7500 instrument (Life Technologies). The expression data was normalized to the small RNA SNORD48 expression levels (Exiqon 203903) and for relative quantification we used the comparative ∆Ct method [46].

### Identification of known small RNAs other than miRNAs

In order to search for small RNAs other than miRNAs we took the trimmed and filtered sequences that did not match miRBase (i.e., 35 nt long sequences) and matched them to sequences downloaded from public databases containing small RNA sequences: Cogemir [47]; DeepBase [48]; piRNABank [49]; smiRNADB [50]; RNAdb [51]; CONDOR [52]; fRNAdb [53], microRNA.org [54]; miRNAMap [55]; NONCODE [56] and UCSC Genome Browser human miRNAs/snoRNA [57].

Hits were restricted to those with 100 % similarity, 100 % coverage of our query or of the subject sequences of the databases, and with identical coordinates in the genome.

### Identification of novel differentially expressed small RNA-like molecules

Sequences that did not match known miRNAs or other small RNAs could be part of novel small RNA-like molecules. In order to evaluate this possibility we used a discovery process based on ab initio classification, similarity search and structural search.

**Fig. 8:**
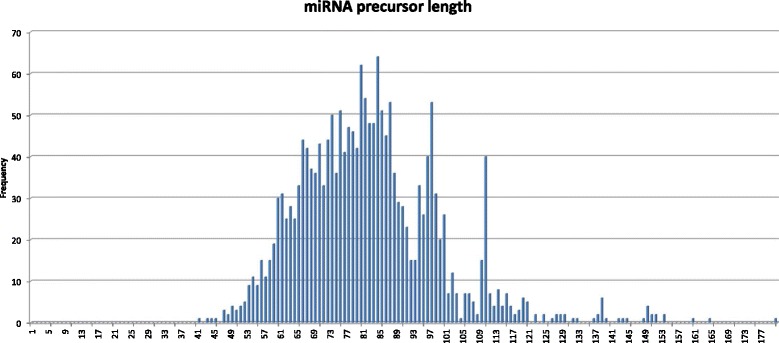
MicroRNA precursor length of sequences deposited in miRBase v20. The bar chart shows the frequency of lengths of human miRNA precursors deposited in miRBase v.20

**Fig. 9:**
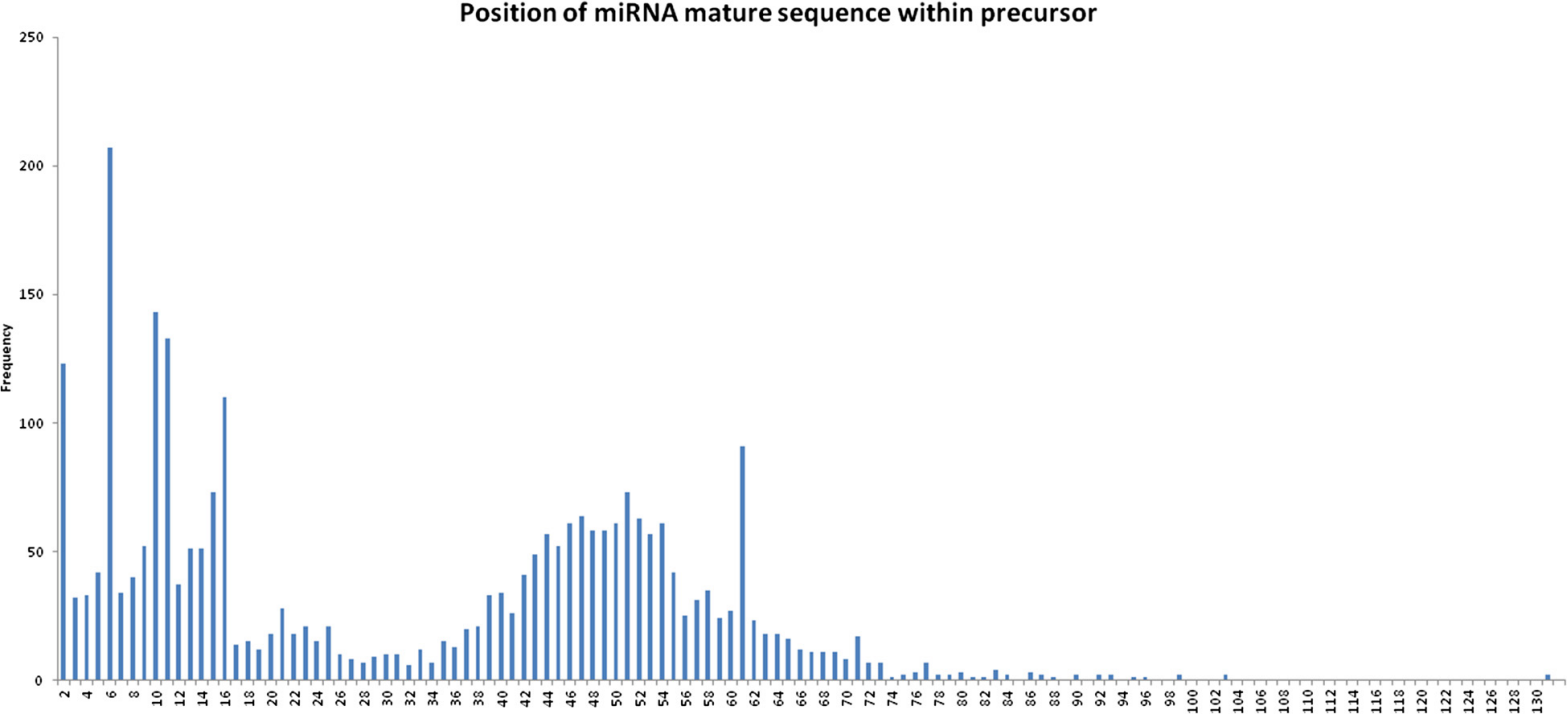
Position of mature miRNAs within their precursor molecules. The bar chart shows the frequency of first positions (first nucleotide) of a mature miRNA sequence within its precursor molecule. We used the miRBase v. 20 for this evaluation

If our differentially expressed reads corresponded to mature miRNAs, they should be part of a larger precursor miRNAs. Characterizing this putative precursor miRNA was the goal of our ab initio classification and structural search. At the time of this publication miRNA precursor sequences deposited in miRBase v.20 presented a maximum of 180 nucleotides in length (Fig. 8) and mature sequences mapped mostly from the 5’ end to the middle of the molecule (Fig. 9). Considering this information, we mapped our reads in the genome and extracted 3 candidate sequences for each read: one 180 nucleotide sequence that extended the original read an additional 25 nucleotides to the 3’ and 120 to the 5’ end in order to consider mature sequences matching to the 5’ of the precursor sequence; one 180 nucleotide sequence that extended the original read an additional 120 nucleotides to the 3’ and 25 to the 5’ end, accounting for mature sequences matching the 3’ end of the precursor sequence; and one that extended the original read 120 nucleotides at each side, accounting for mature sequences matching around the center of the precursor molecules. We named these extended sequences *miRNA-candidates.* We extracted the three candidates from each original read due to the negative impact extra nucleotides can have in ab initio prediction methods based on previous folding.

To detect candidates we applied a pipeline that included: (i) similarity search against the sequences from the 11 databases described in the previous section; (ii) structural search performed against the RFAM [58] using the Infernal for RNA alignment (INFERence of RNA Alignment), version 0.81 [59] with the default parameters and the recommended bitscore cutoff value of 25; (iii) ab initio classification using SnoScan [60] with the recommended cutoff value; SnoReport [61] with a cutoff value of 0.90 and HHMMIR [62] using the BaumWelch trained model and the recommended threshold of 0.71. To use HHMMIR we folded the candidates using RNAfold [63].

After excluding all candidates where the orginal read mapped to known ncRNAs of other families, we considered putative miRNAs those candidate sequences that were classified as miRNAs by RFAM, or those that were classified as miRNAs by HMMIR and did not present positive scores by SnoScan or SnoReport.

Additionally, we required that strong miRNA-candidates should have the original read mapped to the stem region of the predicted secondary structure. When this was the case we looked for a possible mature sequence within the sequenced read using default parameters from the algorithm MatureBayes [64]. Mature sequences were then aligned to the 3’ UTR region of human genes obtained from UTRDB [65] in order to find putative mRNA targets using the miRanda algorithm [66]. Different algorithms come up with a different selection of targets [67]. miRanda was chosen since it is the same algorithm used by mirRBase [68], our primary source of miRNA information in this work, and it has been shown to perform better than other algorithms such as TargetScan and RNAHybrid in terms of specificity in a review tackling this issue [69].

## Additional files

Additional file 1:
**Sequencing results from 8 non-metastatic tumor samples and 10 metastatic tumor samples.** We used miRBase v.20 and human genome hg19 reference sequences for mapping. Number of reads should be multiplied by 10^5^. Additional file 2:
**Complete set of detected mature miRNAs and correspondent read counts in non-metastatic tumor samples.** We used miRBase v.20 as reference for miRNA identification. Read counts are raw numbers (not normalized). Additional file3:
**Complete set of detected mature miRNAs and correspondent read counts in metastatic tumor samples.** We used miRBase v.20 as reference for miRNA identification. Read counts are raw numbers (not normalized). Additional file 4:
**Most expressed miRNAs in non-metastatic tumor samples.** The graph shows the most expressed miRNAs per sample (x-axis) and correspondent read counts (y-axis). Numbers on top of each bar correspond to the cumulative percentage considering the total number of read counts per sample. Additional file 5:
**Most expressed miRNAs in metastatic tumor samples.** The graph shows the most expressed miRNAs per sample (x-axis) and correspondent read counts (y-axis). Numbers on top of each bar correspond to the cumulative percentage considering the total number of read counts per sample. Additional file 6:
**Validation of miRNA expression levels in an additional set of tumor tissue samples.** Average expression levels were considered in this figure and fold-change compares N0 and N+ samples. Negative results indicate over-expression in N+ samples. Additional file 7:
**Complete set of small RNAs other than miRNA identified in tumor samples, with specific type report in non-coding RNA databanks.** For the annotation of small RNAs we used BLAST search against available databanks of non-coding RNA sequences. Additional file 8:
**Complete set of small RNAs other than miRNA identified in tumor samples with evidences from ab initio prediction reported in non-coding RNA databanks.** The annotation procedure of this set of small RNAs used BLAST search against available databanks of non-coding RNA sequences but reports showed only evidences from ab initio prediction. Additional file 9:
**Expression levels of all small RNAs other than miRNA identified in tumor samples.** The annotation procedure of this set of small RNAs used BLAST search against available databanks of non-coding RNA sequences but reports showed only evidences from ab initio prediction.

## References

[1] Moazed D (2009). Small RNAs in transcriptional gene silencing and genome defence. Nature.

[2] Farazi TA, Juranek SA, Tuschl T (2008). he growing catalog of small RNAs and their association with distinct Argonaute/Piwi family members. Development.

[3] Lee YS, Shibata Y, Malhotra A, Dutta A (2009). A novel class of small RNAs: tRNA-derived RNA fragments (tRFs). Genes Dev.

[4] Denli AM, Tops BB, Plasterk RH, Ketting RF, Hannon GJ (2004). Processing of primary microRNAs by the microprocessor complex. Nature.

[5] Kim VN, Han J, Siomi MC (2009). Biogenesis of small RNAs in animals. Nat Rev Mol Cell Biol.

[6] Filipowicz W, Jaskiewicz L, Kolb FA, Pillai RS (2005). Post-transcriptional gene silencing by siRNAs and miRNAs. Curr Opin Struct Biol.

[7] Lee RC, Feinbaum RL, Ambros V (1993). The C elegans heterochronic gene lin-4 encodes small RNAs with antisense complementarity to lin-14. Cel.

[8] Calin GA, Croce CM (2006). MicroRNA signatures in human cancers. Nat Rev Cancer.

[9] Lee YS, Dutta A (2009). MicroRNAs in cancer. Annu Rev Pathol.

[10] Dykxhoorn DM (2010). MicroRNAs and metastasis: little RNAs go a long way. Cancer Res.

[11] Ruf CG, Dinger D, Port M, Schmelz HU, Wagner W, Matthies C (2014). Small RNAs in the peripheral blood discriminate metastasized from non-metastasized seminoma. Mol Cancer.

[12] The Cancer Genome Atlas. http://cancergenome.nih.gov/.

[13] Zovoilis A, Mungall AJ, Moore R, Varhol R, Chu A, Wong TN (2014). he expression level of small non-coding RNAs derived from the first exon of protein-coding genes is predictive of cancer status. EMBO reports.

[14] Jemal A, Bray F, Center MM, Ferlay J, Ward E, Forman D (2011). Global cancer statistics. CA: a cancer journal for clinicians.

[15] Zhang ZF, Morgenstern H, Spitz MR, Tashkin DP, Yu GP, Hsu TC (2000). Environmental tobacco smoking, mutagen sensitivity, and head and neck squamous cell carcinoma. Cancer epidemiology, biomarkers & prevention : a publication of the American Association for Cancer Research, cosponsored by the American Society of Preventive Oncology.

[16] Leemans CR, Braakhuis BJ, Brakenhoff RH (2011). The molecular biology of head and neck cancer. Nat Rev Cancer.

[17] Warnakulasuriya S (2010). Living with oral cancer: epidemiology with particular reference to prevalence and life-style changes that influence survival. Oral Oncol.

[18] Genden EM, Ferlito A, Bradley PJ, Rinaldo A, Scully C (2003). Neck disease and distant metastases. Oral Oncol.

[19] Fletcher AM, Heaford AC, Trask DK (2008). Detection of metastatic head and neck squamous cell carcinoma using the relative expression of tissue-specific Mir-205. Transl Oncol..

[20] Connolly EC, Van Doorslaer K, Rogler LE, Rogler CE (2010). Overexpression of miR-21 promotes an in vitro metastatic phenotype by targeting the tumor suppressor RHOB. Molecular cancer research : MCR.

[21] Li J, Zhang Y, Zhang W, Jia S, Tian R, Kang Y (2013). Genetic heterogeneity of breast cancer metastasis may be related to miR-21 regulation of TIMP-3 in translation. International journal of surgical oncology.

[22] Bornachea O, Santos M, Martinez-Cruz AB, Garcia-Escudero R, Duenas M, Costa C (2012). EMT and induction of miR-21 mediate metastasis development in Trp53-deficient tumours. Scientific reports.

[23] Kawakita A, Yanamoto S, Yamada SI, Naruse T, Takahashi H, Kawasaki G et al. MicroRNA-21 Promotes Oral Cancer Invasion via the Wnt/beta-Catenin Pathway by Targeting DKK2. Pathology oncology research : POR. 2013. doi:10.1007/s12253-013-9689-y. 10.1007/s12253-013-9689-y 23999978

[24] Severino P, Oliveira LS, Torres N, Andreghetto FM, Klingbeil Mde F, Moyses R (2013). High-throughput sequencing of small RNA transcriptomes reveals critical biological features targeted by microRNAs in cell models used for squamous cell cancer research. BMC Genomics.

[25] Yi R, Poy MN, Stoffel M, Fuchs E (2008). A skin microRNA promotes differentiation by repressing 'stemness'. Nature.

[26] Sonkoly E, Wei T, Pavez Lorie E, Suzuki H, Kato M, Torma H (2010). Protein kinase C-dependent upregulation of miR-203 induces the differentiation of human keratinocytes. The Journal of investigative dermatology.

[27] Sonkoly E, Loven J, Xu N, Meisgen F, Wei T, Brodin P et al. MicroRNA-203 functions as a tumor suppressor in basal cell carcinoma. Oncogenesis. 2012;1:e3. doi:10.1038/oncsis.2012.3. 10.1038/oncsis.2012.3 PMC3412636 23552555

[28] Zhang Z, Zhang B, Li W, Fu L, Zhu Z, Dong JT (2011). Epigenetic silencing of miR-203 upregulates SNAI2 and contributes to the invasiveness of malignant breast cancer cells. Genes & cancer.

[29] O'Day E, Lal A (2010). MicroRNAs and their target gene networks in breast cancer. Breast cancer research : BCR.

[30] Valastyan S, Chang A, Benaich N, Reinhardt F, Weinberg RA (2011). Activation of miR-31 function in already-established metastases elicits metastatic regression. Genes Dev.

[31] Liu CJ, Tsai MM, Hung PS, Kao SY, Liu TY, Wu KJ (2010). miR-31 ablates expression of the HIF regulatory factor FIH to activate the HIF pathway in head and neck carcinoma. Cancer research.

[32] Hui AB, Lenarduzzi M, Krushel T, Waldron L, Pintilie M, Shi W (2010). Comprehensive MicroRNA profiling for head and neck squamous cell carcinomas. Clinical cancer research : an official journal of the American Association for Cancer Research.

[33] Hung PS, Tu HF, Kao SY, Yang CC, Liu CJ, Huang TY (2014). miR-31 is upregulated in oral premalignant epithelium and contributes to the immortalization of normal oral keratinocytes. Carcinogenesis.

[34] Zhao Y, Miao G, Li Y, Isaji T, Gu J, Li J (2014). MicroRNA- 130b suppresses migration and invasion of colorectal cancer cells through downregulation of integrin beta1 [corrected]. PLoS One.

[35] Yang CC, Hung PS, Wang PW, Liu CJ, Chu TH, Cheng HW (2011). miR-181 as a putative biomarker for lymph-node metastasis of oral squamous cell carcinoma. Journal of oral pathology & medicine : official publication of the International. Association of Oral Pathologists and the American Academy of Oral Pathology.

[36] Liu X, Chen Q, Yan J, Wang Y, Zhu C, Chen C (2013). MiRNA-296-3p-ICAM-1 axis promotes metastasis of prostate cancer by possible enhancing survival of natural killer cell-resistant circulating tumour cells. Cell death & disease.

[37] Wong TS, Liu XB, Wong BY, Ng RW, Yuen AP, Wei WI (2008). Mature miR-184 as potential oncogenic microRNA of squamous cell carcinoma of tongue. Clinical cancer research : an official journal of the American Association for Cancer Research.

[38] Blondal T, Jensby Nielsen S, Baker A, Andreasen D, Mouritzen P, Wrang Teilum M (2013). Assessing sample and miRNA profile quality in serum and plasma or other biofluids. Methods.

[39] Russo F, Di Bella S, Nigita G, Macca V, Lagana A, Giugno R (2012). miRandola: extracellular circulating microRNAs database. PloS one.

[40] Schwarzenbach H, Nishida N, Calin GA, Pantel K (2014). Clinical relevance of circulating cell-free microRNAs in cancer. Nature reviews Clinical oncology.

[41] Lim SL, Ricciardelli C, Oehler MK, Tan IM, Russell D, Grutzner F (2014). Overexpression of piRNA pathway genes in epithelial ovarian cancer. PLoS One.

[42] Williams GT, Farzaneh F (2012). Are snoRNAs and snoRNA host genes new players in cancer?. Nat Rev Cancer.

[43] Mayer BJ, Ren R, Clark KL, Baltimore D (1993). A putative modular domain present in diverse signaling proteins. Cell.

[44] LifeTechnologies. LifeScope™ Genomic Analysis Software 2.5.1. 2012.

[45] Robinson MD, McCarthy DJ, Smyth GK (2010). edgeR: a Bioconductor package for differential expression analysis of digital gene expression data. Bioinformatics.

[46] Livak KJ, Schmittgen TD (2001). Analysis of relative gene expression data using real-time quantitative PCR and the 2(−Delta Delta C(T)) Method. Methods.

[47] Maselli V, Di Bernardo D, Banfi S (2008). CoGemiR: a comparative genomics microRNA database. BMC Genomics.

[48] Yang JH, Shao P, Zhou H, Chen YQ, Qu LH (2010). deepBase: a database for deeply annotating and mining deep sequencing data. Nucleic acids research.

[49] Sai Lakshmi S, Agrawal S. piRNABank: a web resource on classified and clustered Piwi-interacting RNAs. Nucleic acids research. 2008;36(Database issue):D173-7. doi:10.1093/nar/gkm696. 10.1093/nar/gkm696 PMC2238943 17881367

[50] Landgraf P, Rusu M, Sheridan R, Sewer A, Iovino N, Aravin A (2007). A mammalian microRNA expression atlas based on small RNA library sequencing. Cell.

[51] Pang KC, Stephen S, Dinger ME, Engstrom PG, Lenhard B, Mattick JS (2007). RNAdb 2.0–an expanded database of mammalian non-coding RNAs. Nucleic Acids Res.

[52] Woolfe A, Goode DK, Cooke J, Callaway H, Smith S, Snell P (2007). CONDOR: a database resource of developmentally associated conserved non-coding elements. BMC Dev Biol.

[53] Kin T, Yamada K, Terai G, Okida H, Yoshinari Y, Ono Y (2007). fRNAdb: a platform for mining/annotating functional RNA candidates from non-coding RNA sequences. Nucleic Acids Res.

[54] Betel D, Wilson M, Gabow A, Marks DS, Sander C (2008). The microRNA.org resource: targets and expression. Nucleic Acids Res.

[55] Hsu SD, Chu CH, Tsou AP, Chen SJ, Chen HC, Hsu PW (2008). miRNAMap 2.0: genomic maps of microRNAs in metazoan genomes. Nucleic Acids Res.

[56] Bu D, Yu K, Sun S, Xie C, Skogerbo G, Miao R (2012). NONCODE v3.0: integrative annotation of long noncoding RNAs. Nucleic Acids Res.

[57] Fujita PA, Rhead B, Zweig AS, Hinrichs AS, Karolchik D, Cline MS (2011). The UCSC Genome Browser database: update 2011. Nucleic Acids Res.

[58] Griffiths-Jones S. Annotating non-coding RNAs with Rfam. Current protocols in bioinformatics / editoral board, Andreas D Baxevanis [et al.]. 2005;Chapter 12:Unit 12 5. doi:10.1002/0471250953.bi1205s9. 10.1002/0471250953.bi1205s9 18428745

[59] Eddy S. Infernal: inference of RNA alignments. http://infernal.janelia.org/. 10.1093/bioinformatics/btp157 PMC2732312 19307242

[60] Lowe TM, Eddy SR (1999). A computational screen for methylation guide snoRNAs in yeast. Science.

[61] Hertel J, Hofacker IL, Stadler PF (2008). SnoReport: computational identification of snoRNAs with unknown targets. Bioinformatics.

[62] Kadri S, Hinman V, Benos PV. HHMMiR: efficient de novo prediction of microRNAs using hierarchical hidden Markov models. BMC bioinformatics. 2009;10 Suppl 1:S35. doi:10.1186/1471-2105-10-S1-S35. 10.1186/1471-2105-10-S1-S35 PMC2648761 19208136

[63] RNAfold WebServer. http://rna.tbi.univie.ac.at/cgi-bin/RNAfold.cgi.

[64] Gkirtzou K, Tsamardinos I, Tsakalides P, Poirazi P (2010). MatureBayes: a probabilistic algorithm for identifying the mature miRNA within novel precursors. PLoS One.

[65] Grillo G, Turi A, Licciulli F, Mignone F, Liuni S, Banfi S (2010). UTRdb and UTRsite (RELEASE 2010): a collection of sequences and regulatory motifs of the untranslated regions of eukaryotic mRNAs. Nucleic Acids Res.

[66] Enright AJ, John B, Gaul U, Tuschl T, Sander C, Marks DS (2003). MicroRNA targets in Drosophila. Genome Biol.

[67] Liu B, Li J, Cairns MJ (2014). Identifying miRNAs, targets and functions. Brief Bioinform.

[68] Griffiths-Jones S, Grocock RJ, van Dongen S, Bateman A, Enright AJ. miRBase: microRNA sequences, targets and gene nomenclature. Nucleic acids research. 2006;34(Database issue):D140-4. doi:10.1093/nar/gkj112. 10.1093/nar/gkj112 PMC1347474 16381832

[69] Zhang Y, Verbeek FJ. Comparison and integration of target prediction algorithms for microRNA studies. Journal of integrative bioinformatics. 2010;7(3). doi:10.2390/biecoll-jib-2010-127. 10.2390/biecoll-jib-2010-127 20375447

