# Supplementary material for: Small RNAs in metastatic and non-metastatic oral squamous cell carcinoma
Source: BMC Med Genomics. 2015 Jun 24;8:31. doi: 10.1186/s12920-015-0102-4 (PMC4479233; doi:10.1186/s12920-015-0102-4)
Supplement: Additional file 1: — Sequencing results from 8 non-metastatic tumor samples and 10 metastatic tumor samples. We used miRBase v.20 and human genome hg19 reference sequences for mapping. Number of reads should be multiplied by 105. [file 12920_2015_102_MOESM1_ESM.pdf]

Additional File 1: Sequencing results from 8 non-metastatic tumor samples and 10 metastatic tumor samples.

Non-metastatic	p0040	%	p0151	%	p0291	%	p340	%	p0418	%	p0486	%	p1022	%	p1125	%				
Total of reads	52.6	100.0	92.5	100.0	69.1	100.0	39.7	100.0	55.9	100.0	55.5	100.0	42.1	100.0	59.3	100.0				
Reads matching miRBase	12.6	23.9	11.3	12.2	1.2	1.8	2.9	7.4	13.6	24.4	1.0	1.8	0.1	0.3	3.0	5.1				
Reads matching genome	12.8	24.4	36.2	39.1	27.6	39.9	8.7	22.0	14.9	26.7	9.7	17.5	10.1	24.1	25.3	42.6				
Filtered out reads	23.8	45.3	29.1	31.5	34.7	50.2	25.5	64.1	24.3	43.4	40.7	73.3	28.6	67.9	27.5	46.3				
Unmapped reads	3.4	6.4	15.9	17.2	5.6	8.1	2.6	6.6	3.1	5.6	4.1	7.4	3.2	7.6	3.6	6.0				
**Metastatic**	**p0012**	**%**	**p0280**	**%**	**p374**	**%**	**p397**	**%**	**p0441**	**%**	**p0652**	**%**	**p0677**	**%**	**p1231**	**%**	**p1381**	**%**	**p1642**	**%**
Total of reads	74.1	100.0	50.7	100.0	53.6	100.0	62.6	100.0	72.4	100.0	40.1	100.0	47.3	100.0	41.0	100.0	51.2	100.0	67.5	100.0
Reads matching miRBase	1.8	2.4	1.0	2.0	0.3	0.5	13.7	21.8	2.3	3.2	4.6	11.6	2.3	4.8	1.2	2.9	0.4	0.7	1.8	2.6
Reads matching genome	37.7	50.9	6.8	13.3	12.6	23.6	13.8	22.1	17.3	23.9	5.7	14.2	8.9	18.9	5.1	12.3	7.6	14.8	29.2	43.2
Filtered out reads	29.0	39.2	40.0	78.8	37.5	69.9	29.7	47.4	45.6	63.0	27.5	68.4	33.2	70.1	33.3	81.2	40.0	78.1	30.4	45.0
Unmapped reads	5.5	7.5	3.0	5.9	3.2	6.0	5.4	8.7	7.2	9.9	2.3	5.8	2.9	6.2	1.4	3.5	3.3	6.4	6.2	9.2
