# Supplementary material for: Small RNAs in metastatic and non-metastatic oral squamous cell carcinoma
Source: BMC Med Genomics. 2015 Jun 24;8:31. doi: 10.1186/s12920-015-0102-4 (PMC4479233; doi:10.1186/s12920-015-0102-4)
Supplement: Additional file 2: — Complete set of detected mature miRNAs and correspondent read counts in non-metastatic tumor samples. We used miRBase v.20 as reference for miRNA identification. Read counts are raw numbers (not normalized). [file 12920_2015_102_MOESM2_ESM.pdf]

p0040		p0151		p0291		p340		p0418		p0486		p1022		p1125	
**MiRNAs**	**Counts**	**MiRNAs**	**Counts**	**MiRNAs**	**Counts**	**MiRNAs**	**Counts**	**MiRNAs**	**Counts**	**MiRNAs**	**Counts**	**MiRNAs**	**Counts**	**MiRNAs**	**Counts**
let-7a	14754	let-7a	5337	let-7a	1068	let-7a	3574	let-7a	6609	let-7a	450	let-7a	101	let-7a	937
let-7b	11270	let-7b	3789	let-7b	1784	let-7f	3705	let-7b	6064	let-7b	746	let-7b	106	let-7b	3178
let-7c	3176	let-7c	265	let-7c	138	let-7i	1050	let-7c	1075	let-7c	84	let-7c	7	let-7c	266
let-7d	3842	let-7d	3890	let-7d	507	mir-1	145	let-7d	3652	let-7d	326	let-7d	74	let-7d	349
let-7e	1888	let-7e	968	let-7e	262	mir-101	17	let-7e	1566	let-7e	127	let-7e	4	let-7e	76
let-7f	7049	let-7f	5708	let-7f	533	mir-103a	16474	let-7f	9948	let-7f	475	let-7f	132	let-7f	585
let-7g	19834	let-7g	12658	let-7g	1988	mir-106b	459	let-7g	40827	let-7g	1302	let-7g	223	let-7g	1709
let-7i	6059	let-7i	2545	let-7i	303	mir-107	2898	let-7i	9034	let-7i	326	let-7i	17	let-7i	1657
mir-1	100	mir-1	1459	mir-1	254	mir-1182	2	mir-1	960	mir-1	16	mir-1	12	mir-1	130
mir-100	2475	mir-100	7200	mir-100	1285	mir-1185	16	mir-100	6808	mir-100	460	mir-100	66	mir-100	1907
mir-101	279	mir-101	107	mir-101	29	mir-1228	1	mir-101	241	mir-101	10	mir-101	6	mir-101	38
mir-103a	32162	mir-103a	31658	mir-103a	4480	mir-1245a	2	mir-103a	23180	mir-103a	2234	mir-103a	237	mir-103a	2903
mir-106a	25	mir-105	6	mir-105	2	mir-1247	7	mir-106a	25	mir-106a	15	mir-106a	1	mir-106a	3
mir-106b	1097	mir-106a	25	mir-106a	4	mir-1255a	1	mir-106b	1220	mir-106b	174	mir-106b	30	mir-106b	137
mir-107	5223	mir-106b	1404	mir-106b	83	mir-1256	2	mir-107	3120	mir-107	303	mir-107	117	mir-107	549
mir-10a	1088	mir-107	4975	mir-107	655	mir-125b	273	mir-10a	340	mir-10a	91	mir-10a	4	mir-10a	23
mir-10b	3613	mir-10a	288	mir-10a	67	mir-1260b	302	mir-10b	2321	mir-10b	150	mir-10b	38	mir-10b	431
mir-1180	10	mir-10b	1483	mir-10b	246	mir-1268a	3	mir-1179	3	mir-1180	6	mir-1180	1	mir-1180	8
mir-1185	17	mir-1179	2	mir-1185	8	mir-127	14	mir-1180	4	mir-1185	2	mir-1227	1	mir-1185	10
mir-1226	26	mir-1180	16	mir-122	16	mir-1273d	3	mir-1185	25	mir-122	4	mir-1247	2	mir-1199	1
mir-1227	10	mir-1185	34	mir-1226	10	mir-128	73	mir-1197	3	mir-1226	10	mir-1252	2	mir-1203	1
mir-1228	5	mir-1197	6	mir-1227	4	mir-1285	7	mir-122	6	mir-1227	2	mir-125a	36	mir-1226	14
mir-1236	8	mir-1199	1	mir-1247	16	mir-1287	2	mir-1226	15	mir-1228	1	mir-125b	42	mir-1227	4
mir-1237	1	mir-1207	1	mir-1250	1	mir-1289	2	mir-1227	2	mir-1229	4	mir-126	298	mir-1228	3
mir-1247	7	mir-122	2	mir-1252	6	mir-129	3	mir-1228	7	mir-1237	3	mir-1260b	28	mir-1229	2
mir-1252	2	mir-1225	2	mir-125a	2095	mir-1307	82	mir-1233	2	mir-1247	2	mir-1277	7	mir-1236	3
mir-1255a	2	mir-1226	36	mir-125b	995	mir-1323	2	mir-1237	4	mir-1252	2	mir-128	7	mir-1247	30
mir-1255b	2	mir-1227	13	mir-126	4761	mir-133a	51	mir-1247	148	mir-125a	648	mir-129	1	mir-1255b	1
mir-1256	8	mir-1228	13	mir-1260b	125	mir-133b	45	mir-1252	2	mir-125b	370	mir-1304	1	mir-1256	2
mir-125a	5583	mir-1229	2	mir-127	9	mir-1343	2	mir-1256	2	mir-126	1607	mir-1306	2	mir-125a	1046
mir-125b	2960	mir-1234	4	mir-1271	6	mir-135a	1	mir-125a	8714	mir-1260b	143	mir-1307	2	mir-125b	667

mir-126	46335	mir-1236	1	mir-1277	9	mir-135b	525	mir-125b	4679	mir-1268a	2	mir-130a	139	mir-126	4991
mir-1260b	363	mir-1237	5	mir-128	28	mir-138	46	mir-126	59499	mir-127	1	mir-130b	2452	mir-1260b	171
mir-1269b	2	mir-1247	34	mir-1287	2	mir-141	1107	mir-1260b	256	mir-1271	6	mir-135b	14	mir-1268a	3
mir-127	59	mir-1250	1	mir-129	4	mir-146b	143	mir-1269b	19	mir-1273g	1	mir-136	6	mir-127	26
mir-1271	70	mir-125a	11116	mir-1292	2	mir-147b	4	mir-127	107	mir-1277	14	mir-138	2	mir-1271	8
mir-1273g	1	mir-125b	3333	mir-1307	6	mir-148b	185	mir-1271	60	mir-128	34	mir-139	4	mir-1273d	1
mir-1277	12	mir-126	50676	mir-130a	685	mir-151b	10	mir-1273g	1	mir-1285	2	mir-140	30	mir-1273g	5
mir-128	121	mir-1260b	1249	mir-130b	540	mir-1537	10	mir-1273h	5	mir-1287	3	mir-141	15	mir-1273h	3
mir-1285	19	mir-127	113	mir-132	39	mir-15a	753	mir-1277	16	mir-129	1	mir-142	68	mir-1277	1
mir-1287	2	mir-1271	48	mir-133a	39	mir-16	11977	mir-128	178	mir-1301	3	mir-143	30	mir-128	36
mir-129	5	mir-1273d	2	mir-133b	10	mir-17	3619	mir-1285	5	mir-1304	2	mir-144	31	mir-1285	4
mir-1292	5	mir-1273g	3	mir-134	9	mir-181a	344	mir-1287	5	mir-1307	27	mir-145	92	mir-1287	2
mir-1295a	8	mir-1273h	2	mir-1343	6	mir-181b	75	mir-129	4	mir-130a	698	mir-146a	12	mir-129	2
mir-1296	12	mir-1277	43	mir-135a	22	mir-181c	45	mir-1292	6	mir-130b	4423	mir-146b	8	mir-1296	10
mir-1301	27	mir-128	529	mir-135b	90	mir-186	395	mir-1296	16	mir-132	6	mir-148a	172	mir-1301	17
mir-1304	2	mir-1285	11	mir-136	16	mir-190b	2	mir-1301	29	mir-133a	6	mir-148b	4	mir-1304	4
mir-1306	26	mir-1287	16	mir-138	21	mir-1915	1	mir-1304	3	mir-134	10	mir-149	8	mir-1306	6
mir-1307	197	mir-129	13	mir-139	72	mir-193b	1413	mir-1306	40	mir-1343	1	mir-150	182	mir-1307	158
mir-130a	12938	mir-1292	2	mir-140	616	mir-194	4	mir-1307	336	mir-135b	225	mir-151a	26	mir-130a	4397
mir-130b	2199	mir-1296	36	mir-141	67	mir-196a	18	mir-130a	9189	mir-136	12	mir-152	10	mir-130b	524
mir-132	440	mir-1301	47	mir-142	539	mir-196b	42	mir-130b	1472	mir-138	19	mir-155	18	mir-132	92
mir-133a	17	mir-1304	1	mir-143	828	mir-197	211	mir-132	311	mir-139	14	mir-15a	17	mir-133a	32
mir-133b	4	mir-1306	108	mir-144	68	mir-1972	3	mir-133a	123	mir-140	176	mir-15b	26	mir-133b	9
mir-134	10	mir-1307	215	mir-145	4143	mir-1976	1	mir-133b	80	mir-141	137	mir-16	104	mir-134	8
mir-1343	7	mir-130a	10022	mir-146a	162	mir-199a	387	mir-134	83	mir-142	196	mir-17	181	mir-1343	2
mir-135a	11	mir-130b	3802	mir-146b	68	mir-199b	1582	mir-1343	3	mir-143	293	mir-181a	46	mir-135a	1
mir-135b	898	mir-132	578	mir-147b	1	mir-19b	3163	mir-135a	28	mir-144	71	mir-181b	20	mir-135b	376
mir-136	106	mir-1323	2	mir-148a	1417	mir-200b	494	mir-135b	1894	mir-145	1096	mir-181c	2	mir-136	100
mir-138	920	mir-133a	442	mir-148b	76	mir-203a	14445	mir-136	294	mir-146a	50	mir-181d	2	mir-138	107
mir-139	136	mir-133b	207	mir-149	153	mir-205	88267	mir-137	12	mir-146b	67	mir-182	12	mir-139	12
mir-140	864	mir-134	67	mir-150	2474	mir-2054	3	mir-138	612	mir-147b	2	mir-183	2	mir-140	262
mir-141	5584	mir-1343	10	mir-151a	164	mir-2110	8	mir-139	460	mir-148a	894	mir-185	19	mir-141	1695
mir-142	6039	mir-135a	1	mir-151b	11	mir-214	214	mir-140	1678	mir-148b	62	mir-186	21	mir-142	291

mir-143	6253	mir-135b	1549	mir-152	188	mir-218	26	mir-141	2260	mir-149	267	mir-187	2	mir-143	1852
mir-144	330	mir-136	207	mir-1537	1	mir-219a	11	mir-142	9397	mir-150	568	mir-188	3	mir-144	182
mir-145	11206	mir-138	143	mir-154	5	mir-219b	4	mir-143	8642	mir-151a	152	mir-18a	28	mir-145	4680
mir-1468	1	mir-139	186	mir-155	33	mir-22	2032	mir-144	80	mir-151b	7	mir-18b	5	mir-1469	2
mir-146a	736	mir-140	3724	mir-15a	147	mir-221	4210	mir-145	16798	mir-152	94	mir-190a	1	mir-146a	107
mir-146b	396	mir-141	1992	mir-15b	498	mir-2276	1	mir-1469	2	mir-154	16	mir-191	126	mir-146b	63
mir-147b	27	mir-142	5415	mir-16	1632	mir-2355	24	mir-146a	695	mir-155	42	mir-1914	2	mir-147b	2
mir-148a	13937	mir-143	6543	mir-17	594	mir-23a	18605	mir-146b	1233	mir-15a	131	mir-192	12	mir-148a	1307
mir-148b	1702	mir-144	129	mir-181a	772	mir-24	12757	mir-147b	51	mir-15b	234	mir-193a	10	mir-148b	153
mir-149	646	mir-145	15208	mir-181b	55	mir-2682	2	mir-148a	15454	mir-16	1021	mir-193b	65	mir-149	116
mir-150	1529	mir-1468	2	mir-181c	30	mir-26a	4218	mir-148b	992	mir-17	781	mir-195	7	mir-150	52
mir-151a	775	mir-146a	535	mir-181d	9	mir-26b	458	mir-149	1434	mir-181a	172	mir-197	3	mir-151a	203
mir-151b	24	mir-146b	1164	mir-182	133	mir-27a	15707	mir-150	14410	mir-181b	82	mir-199a	66	mir-151b	3
mir-152	1132	mir-147b	36	mir-183	29	mir-29b	2172	mir-151a	813	mir-181c	24	mir-199b	211	mir-152	261
mir-153	2	mir-148a	6606	mir-185	167	mir-29c	3554	mir-151b	20	mir-181d	11	mir-19a	28	mir-1537	9
mir-1537	8	mir-148b	395	mir-186	97	mir-301a	777	mir-152	1225	mir-182	96	mir-19b	57	mir-154	69
mir-154	58	mir-149	2226	mir-187	50	mir-3074	3	mir-153	3	mir-1827	2	mir-200a	32	mir-155	6
mir-155	92	mir-150	7052	mir-188	5	mir-30c	74	mir-1537	10	mir-183	46	mir-200b	48	mir-15a	850
mir-15a	2535	mir-151a	1705	mir-18a	69	mir-30e	1212	mir-154	205	mir-184	2	mir-200c	26	mir-15b	951
mir-15b	5290	mir-151b	47	mir-18b	5	mir-31	60737	mir-155	152	mir-185	181	mir-203a	974	mir-16	4590
mir-16	17151	mir-152	2802	mir-1909	4	mir-3135a	2	mir-15a	2112	mir-186	83	mir-204	1	mir-17	1494
mir-17	10403	mir-1537	7	mir-190a	2	mir-3140	1	mir-15b	3794	mir-187	40	mir-205	1090	mir-181a	110
mir-181a	963	mir-154	131	mir-190b	1	mir-3170	8	mir-16	29462	mir-188	2	mir-206	26	mir-181b	36
mir-181b	52	mir-155	132	mir-191	1401	mir-3180	1	mir-17	6372	mir-18a	245	mir-20a	68	mir-181c	58
mir-181c	293	mir-15a	2445	mir-1914	4	mir-3181	5	mir-181a	1946	mir-18b	3	mir-20b	4	mir-181d	4
mir-181d	19	mir-15b	2768	mir-1915	3	mir-3199	1	mir-181b	134	mir-1909	2	mir-21	860	mir-182	269
mir-182	1043	mir-16	23550	mir-192	52	mir-320b	6	mir-181c	217	mir-190a	8	mir-210	40	mir-183	250
mir-183	460	mir-17	5846	mir-193a	94	mir-320c	27	mir-181d	16	mir-190b	4	mir-212	2	mir-184	2
mir-185	1181	mir-181a	1379	mir-193b	947	mir-320d	1	mir-182	752	mir-191	891	mir-214	38	mir-185	240
mir-186	607	mir-181b	158	mir-195	254	mir-323a	11	mir-183	296	mir-1910	3	mir-215	2	mir-186	234
mir-187	778	mir-181c	380	mir-196a	2	mir-326	70	mir-184	1	mir-1914	1	mir-22	30	mir-187	77
mir-188	158	mir-181d	43	mir-196b	2	mir-329	6	mir-185	1872	mir-192	61	mir-221	98	mir-188	33
mir-18a	1906	mir-182	2835	mir-197	46	mir-331	295	mir-186	1523	mir-193a	135	mir-222	9	mir-18a	184

mir-18b	59	mir-1827	6	mir-1976	1	mir-34a	1843	mir-187	321	mir-193b	986	mir-223	504	mir-18b	19
mir-1909	1	mir-183	843	mir-199a	1459	mir-3591	1	mir-188	200	mir-195	81	mir-224	21	mir-1908	1
mir-190a	57	mir-185	2180	mir-199b	4150	mir-3605	5	mir-18a	1228	mir-196a	4	mir-2277	2	mir-1909	4
mir-190b	2	mir-186	1421	mir-19a	171	mir-3620	6	mir-18b	53	mir-196b	6	mir-23a	563	mir-190a	4
mir-191	7386	mir-187	570	mir-19b	376	mir-3659	2	mir-1909	7	mir-197	54	mir-23b	276	mir-191	650
mir-1910	11	mir-188	244	mir-200a	184	mir-3685	3	mir-190a	18	mir-199a	681	mir-23c	46	mir-1910	8
mir-1911	1	mir-18a	608	mir-200b	199	mir-369	17	mir-190b	3	mir-199b	1735	mir-24	302	mir-1914	2
mir-192	199	mir-18b	56	mir-200c	220	mir-3691	4	mir-191	4178	mir-19a	370	mir-25	30	mir-1915	1
mir-193a	641	mir-1908	5	mir-203a	5165	mir-374b	91	mir-1910	6	mir-19b	398	mir-2682	2	mir-192	78
mir-193b	1494	mir-1909	24	mir-204	5	mir-376a	45	mir-192	232	mir-200a	282	mir-26a	306	mir-193a	412
mir-195	1304	mir-190a	59	mir-205	7137	mir-376c	396	mir-193a	245	mir-200b	198	mir-26b	47	mir-193b	887
mir-196a	45	mir-191	13193	mir-2054	2	mir-378f	2	mir-193b	3060	mir-200c	450	mir-27a	220	mir-194	6
mir-196b	55	mir-1910	12	mir-206	279	mir-3908	12	mir-194	16	mir-203a	3702	mir-27b	124	mir-195	237
mir-197	202	mir-1914	3	mir-208b	2	mir-3917	1	mir-195	1992	mir-204	23	mir-28	29	mir-196b	8
mir-199a	10953	mir-192	1039	mir-20a	223	mir-3924	1	mir-196a	37	mir-205	12878	mir-299	4	mir-197	140
mir-199b	38201	mir-193a	230	mir-20b	13	mir-3926	1	mir-196b	43	mir-206	30	mir-29a	167	mir-199a	3367
mir-19a	17017	mir-193b	3288	mir-21	13835	mir-3935	2	mir-197	774	mir-208a	2	mir-29b	36	mir-199b	5348
mir-19b	20158	mir-194	8	mir-210	281	mir-3960	80	mir-1976	2	mir-20a	449	mir-29c	164	mir-19a	1307
mir-200a	13902	mir-195	2231	mir-211	1	mir-3972	6	mir-199a	13617	mir-20b	20	mir-301a	18	mir-19b	2416
mir-200b	2751	mir-196a	10	mir-212	1	mir-409	72	mir-199b	31956	mir-21	17156	mir-301b	4	mir-200a	1648
mir-200c	1399	mir-196b	109	mir-214	1464	mir-411	5	mir-19a	7590	mir-210	275	mir-30a	14	mir-200b	403
mir-202	2	mir-197	717	mir-218	22	mir-4251	1	mir-19b	11241	mir-212	1	mir-30b	25	mir-200c	576
mir-203a	44834	mir-1976	1	mir-22	379	mir-4270	2	mir-200a	4497	mir-214	213	mir-30c	14	mir-203a	5949
mir-203b	14	mir-199a	14112	mir-221	830	mir-429	257	mir-200b	1382	mir-218	12	mir-30d	61	mir-203b	5
mir-204	143	mir-199b	29924	mir-222	123	mir-4293	61	mir-200c	498	mir-219a	2	mir-30e	25	mir-204	7
mir-205	164820	mir-19a	3026	mir-223	7402	mir-4300	1	mir-203a	15635	mir-22	195	mir-31	570	mir-205	95859
mir-206	27	mir-19b	4513	mir-224	255	mir-431	16	mir-203b	13	mir-221	1102	mir-3127	2	mir-2054	2
mir-20a	3599	mir-200a	3816	mir-2355	8	mir-4315	2	mir-204	161	mir-222	109	mir-3152	2	mir-206	198
mir-20b	26	mir-200b	1658	mir-23a	3380	mir-4320	7	mir-205	139971	mir-223	3024	mir-3158	1	mir-208b	4
mir-21	267393	mir-200c	1642	mir-23b	1772	mir-4417	5	mir-206	586	mir-224	125	mir-3160	2	mir-20a	614
mir-210	4775	mir-203a	48512	mir-23c	77	mir-4430	2	mir-208b	6	mir-2278	3	mir-323a	4	mir-20b	14
mir-211	76	mir-203b	44	mir-24	3746	mir-4433b	1	mir-20a	2582	mir-2355	4	mir-324	4	mir-21	49651
mir-2110	3	mir-204	41	mir-25	222	mir-4470	1	mir-20b	96	mir-23a	5676	mir-326	4	mir-210	813

mir-2116	8	mir-205	148774	mir-26a	2776	mir-4472	1	mir-21	336182	mir-23b	1000	mir-329	2	mir-211	1
mir-212	27	mir-206	2918	mir-26b	317	mir-4477a	4	mir-210	5159	mir-23c	152	mir-330	1	mir-2110	9
mir-214	1420	mir-208b	4	mir-27a	972	mir-4480	2	mir-211	63	mir-24	3744	mir-331	22	mir-2114	1
mir-216a	7	mir-20a	1657	mir-27b	274	mir-4500	260	mir-2110	13	mir-25	204	mir-335	4	mir-212	12
mir-217	3	mir-20b	64	mir-28	174	mir-4521	5	mir-2114	5	mir-26a	1764	mir-338	3	mir-214	1950
mir-218	42	mir-21	147552	mir-296	2	mir-4524b	1	mir-212	9	mir-26b	241	mir-339	14	mir-218	24
mir-219a	32	mir-210	1297	mir-299	10	mir-4536	2	mir-214	3967	mir-27a	1383	mir-33a	2	mir-219a	3
mir-219b	4	mir-211	2	mir-29a	6126	mir-454	293	mir-215	6	mir-27b	274	mir-33b	2	mir-22	1886
mir-22	5496	mir-2110	12	mir-29b	388	mir-4632	3	mir-217	4	mir-28	107	mir-340	5	mir-221	1277
mir-221	19204	mir-2116	2	mir-29c	2252	mir-4645	5	mir-218	140	mir-296	26	mir-342	33	mir-222	449
mir-222	3616	mir-212	28	mir-301a	45	mir-466	2	mir-219a	8	mir-29a	1622	mir-345	4	mir-223	3235
mir-223	24980	mir-214	5659	mir-301b	12	mir-4660	1	mir-219b	2	mir-29b	496	mir-34a	22	mir-224	931
mir-224	1924	mir-217	2	mir-3065	2	mir-4671	3	mir-22	4355	mir-29c	934	mir-34b	5	mir-2355	3
mir-2277	8	mir-218	114	mir-30a	124	mir-4689	1	mir-221	9087	mir-301a	216	mir-3609	2	mir-23a	10339
mir-2278	4	mir-219a	4	mir-30b	448	mir-4753	1	mir-222	1982	mir-301b	32	mir-361	12	mir-23b	1242
mir-2355	37	mir-219b	18	mir-30c	104	mir-4781	2	mir-223	55243	mir-3065	9	mir-3613	2	mir-23c	56
mir-23a	51465	mir-22	4926	mir-30d	341	mir-486	6	mir-224	859	mir-30a	38	mir-3616	2	mir-24	2933
mir-23b	20573	mir-221	9910	mir-30e	201	mir-487b	41	mir-2277	6	mir-30b	184	mir-362	10	mir-2467	6
mir-23c	242	mir-222	2497	mir-31	2130	mir-488	1	mir-2278	3	mir-30c	50	mir-365a	14	mir-25	596
mir-24	22554	mir-223	15576	mir-3117	1	mir-501	6	mir-2355	33	mir-30d	159	mir-365b	46	mir-2681	2
mir-25	3060	mir-224	3197	mir-3120	3	mir-5010	2	mir-23a	35225	mir-30e	223	mir-373	1	mir-2682	2
mir-2681	1	mir-2277	11	mir-3135a	1	mir-5047	3	mir-23b	9044	mir-31	2326	mir-374a	10	mir-26a	625
mir-26a	4775	mir-2278	7	mir-3157	2	mir-5095	9	mir-23c	162	mir-3117	4	mir-374b	12	mir-26b	192
mir-26b	1761	mir-2355	28	mir-3177	1	mir-5100	12	mir-24	29868	mir-3129	2	mir-376a	6	mir-27a	5433
mir-27a	27824	mir-2392	2	mir-32	14	mir-512	8	mir-25	2237	mir-3145	4	mir-376b	4	mir-27b	445
mir-27b	6133	mir-23a	60264	mir-320b	9	mir-515	6	mir-26a	16048	mir-3150b	1	mir-376c	61	mir-28	83
mir-28	284	mir-23b	18336	mir-323a	7	mir-516a	3	mir-26b	3145	mir-3152	4	mir-377	1	mir-296	5
mir-296	48	mir-23c	508	mir-323b	2	mir-517a	12	mir-27a	19472	mir-3177	2	mir-378a	13	mir-298	2
mir-299	20	mir-24	24652	mir-324	39	mir-518a	1	mir-27b	3684	mir-3186	1	mir-382	4	mir-299	8
mir-29a	32896	mir-25	3272	mir-326	13	mir-5191	1	mir-28	590	mir-32	4	mir-3972	1	mir-29a	12450
mir-29b	12764	mir-2681	1	mir-328	14	mir-519a	7	mir-296	105	mir-3200	2	mir-423	3	mir-29b	4179
mir-29c	23327	mir-2682	1	mir-329	2	mir-520e	2	mir-299	77	mir-320b	10	mir-424	91	mir-29c	5801
mir-300	1	mir-26a	14358	mir-331	73	mir-521	2	mir-29a	67517	mir-323a	7	mir-425	28	mir-301a	164

mir-301a	2835	mir-26b	1442	mir-335	41	mir-525	4	mir-29b	10331	mir-323b	4	mir-4270	2	mir-301b	8
mir-301b	265	mir-27a	19316	mir-337	14	mir-526a	1	mir-29c	37207	mir-324	37	mir-429	5	mir-3064	4
mir-3064	1	mir-27b	3441	mir-338	18	mir-539	2	mir-301a	2418	mir-326	20	mir-4423	1	mir-3065	11
mir-3065	95	mir-28	1211	mir-339	112	mir-543	43	mir-301b	80	mir-328	24	mir-4433b	2	mir-30a	100
mir-30a	573	mir-296	147	mir-340	22	mir-548a	1	mir-3065	31	mir-329	6	mir-450a	2	mir-30b	224
mir-30b	5830	mir-299	73	mir-342	813	mir-548aa	2	mir-30a	812	mir-330	7	mir-452	1	mir-30c	40
mir-30c	1027	mir-29a	61495	mir-345	33	mir-548ac	3	mir-30b	2741	mir-331	156	mir-4521	1	mir-30d	363
mir-30d	1497	mir-29b	8867	mir-34a	339	mir-548d	1	mir-30c	595	mir-335	14	mir-4524b	1	mir-30e	485
mir-30e	4138	mir-29c	24630	mir-34b	17	mir-548f	2	mir-30d	1581	mir-337	20	mir-454	7	mir-31	6155
mir-31	8754	mir-300	1	mir-34c	10	mir-548o	4	mir-30e	3781	mir-338	28	mir-455	9	mir-3115	2
mir-3117	10	mir-301a	851	mir-3591	1	mir-548q	2	mir-31	33834	mir-339	152	mir-4645	2	mir-3117	1
mir-3126	4	mir-301b	94	mir-3605	8	mir-548x	1	mir-3117	5	mir-33a	10	mir-4662a	2	mir-3121	1
mir-3129	2	mir-302a	1	mir-3607	122	mir-549a	2	mir-3129	11	mir-33b	16	mir-4690	1	mir-3127	2
mir-3144	2	mir-3064	2	mir-361	187	mir-550a	8	mir-3140	2	mir-340	24	mir-4728	2	mir-3129	2
mir-3150b	2	mir-3065	24	mir-3613	21	mir-551a	9	mir-3145	2	mir-342	158	mir-4753	2	mir-3131	2
mir-3151	5	mir-3074	4	mir-3614	2	mir-5684	3	mir-3150b	2	mir-345	35	mir-4763	2	mir-3136	2
mir-3157	8	mir-30a	592	mir-362	22	mir-5697	1	mir-3151	1	mir-34a	269	mir-4777	1	mir-3140	1
mir-3158	2	mir-30b	13983	mir-363	7	mir-570	10	mir-3160	2	mir-34b	43	mir-4799	1	mir-3141	1
mir-3170	2	mir-30c	780	mir-365a	123	mir-5700	2	mir-3170	1	mir-34c	78	mir-484	19	mir-3144	2
mir-3177	4	mir-30d	4170	mir-365b	273	mir-5787	3	mir-3173	8	mir-3607	1	mir-486	3	mir-3150a	2
mir-3180	6	mir-30e	1985	mir-369	18	mir-590	429	mir-3187	2	mir-361	132	mir-487a	2	mir-3157	2
mir-3187	4	mir-31	37730	mir-372	2	mir-603	1	mir-3194	4	mir-3613	39	mir-487b	2	mir-3160	2
mir-3194	4	mir-3117	10	mir-374a	22	mir-6068	1	mir-32	173	mir-3616	2	mir-495	2	mir-3173	4
mir-32	259	mir-3120	2	mir-374b	105	mir-6084	1	mir-3200	8	mir-362	18	mir-497	3	mir-3177	5
mir-3200	4	mir-3121	2	mir-375	15	mir-610	1	mir-320b	46	mir-363	4	mir-5007	1	mir-3186	2
mir-320b	45	mir-3127	3	mir-376a	72	mir-6127	9	mir-323a	31	mir-365a	352	mir-5008	2	mir-3189	1
mir-323a	11	mir-3129	1	mir-376b	25	mir-620	9	mir-323b	16	mir-365b	632	mir-501	1	mir-3192	4
mir-323b	8	mir-3130	2	mir-376c	333	mir-642a	2	mir-324	358	mir-3663	1	mir-502	5	mir-32	25
mir-324	570	mir-3140	2	mir-377	3	mir-644a	4	mir-326	159	mir-3675	2	mir-505	14	mir-3200	4
mir-326	84	mir-3145	8	mir-378a	77	mir-6511a	1	mir-328	116	mir-3677	1	mir-508	4	mir-320b	29
mir-328	18	mir-3152	2	mir-379	13	mir-6511b	2	mir-329	34	mir-3679	1	mir-5089	1	mir-323a	18
mir-329	8	mir-3157	2	mir-381	45	mir-654	46	mir-330	36	mir-3689c	1	mir-5100	1	mir-323b	14
mir-330	33	mir-3162	1	mir-382	17	mir-663b	7	mir-331	990	mir-369	4	mir-512	4	mir-324	341

mir-331	646	mir-3173	11	mir-383	1	mir-664a	45	mir-335	126	mir-370	2	mir-517a	8	mir-326	20
mir-335	78	mir-3177	2	mir-3922	3	mir-665	3	mir-337	509	mir-374a	10	mir-517b	18	mir-328	24
mir-337	157	mir-3189	1	mir-3928	1	mir-6715a	4	mir-338	1222	mir-374b	68	mir-517c	6	mir-329	14
mir-338	600	mir-3191	1	mir-3944	6	mir-6723	7	mir-339	2576	mir-376a	45	mir-518c	16	mir-331	131
mir-339	1538	mir-3194	3	mir-3972	1	mir-6730	1	mir-33a	215	mir-376b	16	mir-519a	4	mir-335	63
mir-33a	368	mir-32	132	mir-409	89	mir-675	35	mir-33b	35	mir-376c	155	mir-519b	2	mir-337	231
mir-33b	104	mir-3200	4	mir-411	3	mir-6780a	1	mir-340	108	mir-377	2	mir-519d	30	mir-338	123
mir-340	124	mir-320b	41	mir-423	117	mir-6781	3	mir-342	5424	mir-378a	76	mir-520g	8	mir-339	356
mir-342	2055	mir-323a	65	mir-424	637	mir-6790	2	mir-345	512	mir-379	18	mir-521	2	mir-33a	38
mir-345	321	mir-323b	32	mir-425	314	mir-6810	4	mir-34a	6702	mir-382	4	mir-524	2	mir-33b	18
mir-34a	5395	mir-324	759	mir-4270	4	mir-6830	1	mir-34b	896	mir-3928	2	mir-526b	1	mir-340	14
mir-34b	232	mir-326	146	mir-429	10	mir-6850	23	mir-34c	1138	mir-3940	2	mir-532	42	mir-342	161
mir-34c	206	mir-328	128	mir-4293	3	mir-6860	1	mir-3605	6	mir-3941	4	mir-548f	2	mir-345	143
mir-3591	2	mir-329	14	mir-431	6	mir-6880	5	mir-3607	44	mir-3960	2	mir-551a	1	mir-34a	869
mir-3605	6	mir-330	39	mir-432	4	mir-7	540	mir-3609	4	mir-409	20	mir-556	2	mir-34b	220
mir-3607	57	mir-331	1049	mir-4320	2	mir-708	179	mir-361	1078	mir-411	3	mir-5588	2	mir-34c	232
mir-361	640	mir-335	36	mir-433	3	mir-7106	2	mir-3613	973	mir-423	130	mir-5590	4	mir-3591	2
mir-3611	2	mir-337	232	mir-4417	8	mir-7112	2	mir-3616	2	mir-424	445	mir-561	2	mir-3609	2
mir-3613	1079	mir-338	171	mir-4423	4	mir-744	144	mir-3619	3	mir-425	254	mir-5684	2	mir-361	115
mir-362	425	mir-339	1789	mir-4433	4	mir-770	1	mir-362	321	mir-4270	1	mir-574	44	mir-3613	35
mir-3620	2	mir-33a	166	mir-4433b	6	mir-7703	1	mir-3620	8	mir-429	60	mir-582	6	mir-3616	1
mir-363	49	mir-33b	32	mir-4450	2	mir-7846	1	mir-363	38	mir-4300	2	mir-590	7	mir-3619	2
mir-365a	380	mir-340	172	mir-4500	8	mir-7974	26	mir-3659	2	mir-4310	1	mir-598	2	mir-362	41
mir-365b	720	mir-342	6796	mir-450a	4	mir-802	1	mir-365a	405	mir-432	4	mir-6084	2	mir-3620	2
mir-3667	1	mir-345	715	mir-450b	4	mir-8083	2	mir-365b	1077	mir-433	2	mir-625	3	mir-363	4
mir-369	46	mir-34a	3615	mir-452	19	mir-874	26	mir-3664	1	mir-4417	4	mir-627	1	mir-365a	86
mir-373	2	mir-34b	86	mir-454	41	mir-889	4	mir-3667	6	mir-4423	1	mir-628	1	mir-365b	171
mir-374a	233	mir-34c	51	mir-455	146	mir-890	4	mir-3678	2	mir-4433	1	mir-6506	2	mir-3667	2
mir-374b	388	mir-3591	2	mir-4668	6	mir-9	2	mir-369	68	mir-4445	1	mir-652	3	mir-3689b	1
mir-374c	6	mir-3605	22	mir-4676	6	mir-92a	1346	mir-370	27	mir-4477a	2	mir-654	2	mir-369	52
mir-375	9	mir-3607	12	mir-4677	2	mir-92b	188	mir-371b	4	mir-4480	2	mir-655	2	mir-370	4
mir-376a	232	mir-361	1008	mir-4685	4	mir-938	1	mir-373	2	mir-4500	29	mir-660	15	mir-373	5
mir-376b	90	mir-3613	601	mir-4687	4	mir-942	5	mir-374a	74	mir-452	57	mir-671	2	mir-374a	10

mir-376c	995	mir-3614	9	mir-4697	2	mir-95	70
mir-377	33	mir-3616	1	mir-4700	4	mir-99b	390
mir-378a	521	mir-362	340	mir-4707	6		
mir-378f	2	mir-3620	14	mir-4708	1		
mir-379	48	mir-363	31	mir-4713	2		
mir-381	41	mir-3649	2	mir-4720	1		
mir-382	28	mir-365a	330	mir-4726	2		
mir-3922	2	mir-365b	684	mir-4728	8		
mir-3925	2	mir-3667	7	mir-4731	1		
mir-3927	2	mir-3675	1	mir-4742	2		
mir-3928	4	mir-3677	1	mir-4750	6		
mir-3940	1	mir-369	100	mir-4753	6		
mir-3972	1	mir-370	5	mir-4762	2		
mir-409	85	mir-3713	1	mir-4783	2		
mir-410	1	mir-373	1	mir-483	4		
mir-411	5	mir-374a	187	mir-484	197		
mir-423	874	mir-374b	450	mir-486	5		
mir-424	3338	mir-374c	6	mir-487a	14		
mir-425	4394	mir-376a	396	mir-487b	58		
mir-4270	10	mir-376b	90	mir-489	10		
mir-429	1918	mir-376c	2067	mir-493	18		
mir-4293	13	mir-377	21	mir-494	38		
mir-4300	5	mir-378a	1439	mir-495	53		
mir-431	10	mir-378f	3	mir-497	23		
mir-4310	2	mir-379	110	mir-499a	2		
mir-432	2	mir-380	8	mir-5000	2		
mir-433	4	mir-381	44	mir-5003	1		
mir-4417	6	mir-382	84	mir-5006	1		
mir-4472	1	mir-3917	1	mir-5008	10		
mir-4500	174	mir-3922	1	mir-501	15		
mir-452	1724	mir-3927	2	mir-502	3		
mir-4521	39	mir-3928	1	mir-503	11		
mir-4536	4	mir-3940	13	mir-505	12		

mir-374b	489	mir-4521	3	mir-6715a	1	mir-374b	20
mir-375	12	mir-454	77	mir-6718	2	mir-375	1
mir-376a	518	mir-455	105	mir-6737	1	mir-376a	83
mir-376b	227	mir-4649	2	mir-675	1	mir-376b	165
mir-376c	1844	mir-4677	4	mir-6753	1	mir-376c	552
mir-377	15	mir-4687	1	mir-6788	2	mir-377	13
mir-378a	701	mir-4694	2	mir-6791	1	mir-378a	375
mir-378f	2	mir-4700	1	mir-6793	2	mir-379	16
mir-379	94	mir-4707	1	mir-6826	2	mir-380	4
mir-380	20	mir-4713	2	mir-6833	2	mir-381	21
mir-381	58	mir-4742	2	mir-6844	2	mir-382	39
mir-382	161	mir-4762	2	mir-6855	1	mir-3922	1
mir-3912	5	mir-4772	4	mir-6857	2	mir-3928	3
mir-3928	4	mir-4777	3	mir-6871	2	mir-3940	8
mir-3934	6	mir-4778	2	mir-7	16	mir-3944	4
mir-3940	9	mir-4789	2	mir-708	36	mir-3960	50
mir-3944	2	mir-483	8	mir-7157	4	mir-3972	1
mir-3960	1	mir-484	280	mir-744	2	mir-409	49
mir-409	257	mir-485	4	mir-767	1	mir-410	9
mir-410	2	mir-486	14	mir-874	6	mir-411	4
mir-411	37	mir-487a	6	mir-889	1	mir-423	160
mir-412	2	mir-487b	12	mir-9	2	mir-424	2270
mir-423	839	mir-493	10	mir-92a	42	mir-425	656
mir-424	5096	mir-494	7	mir-92b	2	mir-4270	2
mir-425	2874	mir-495	12	mir-93	69	mir-429	345
mir-4251	1	mir-497	37	mir-942	2	mir-4300	1
mir-4270	23	mir-5000	1	mir-95	2	mir-431	9
mir-429	632	mir-5001	2	mir-96	1	mir-4310	1
mir-4293	39	mir-5008	6	mir-98	9	mir-432	15
mir-4300	3	mir-500a	2	mir-99a	107	mir-4320	2
mir-431	32	mir-501	8	mir-99b	106	mir-433	10
mir-432	8	mir-5011	2			mir-4417	29
mir-433	5	mir-502	2			mir-4430	5

mir-454	729	mir-3944	32	mir-508	2
mir-455	1357	mir-3960	4	mir-5089	1
mir-4635	1	mir-3972	1	mir-5100	4
mir-4645	2	mir-409	304	mir-511	4
mir-4652	2	mir-410	12	mir-512	12
mir-4653	3	mir-411	25	mir-515	4
mir-4659a	2	mir-422a	2	mir-516b	2
mir-4662a	14	mir-423	586	mir-517a	8
mir-4664	2	mir-424	4898	mir-517b	22
mir-4667	3	mir-425	5770	mir-517c	11
mir-4677	1	mir-4251	2	mir-5187	3
mir-4685	1	mir-4260	1	mir-518c	18
mir-4690	4	mir-4270	25	mir-518d	3
mir-4707	8	mir-429	458	mir-518e	1
mir-4708	2	mir-4300	4	mir-519a	4
mir-4720	2	mir-431	10	mir-519b	2
mir-4722	2	mir-4310	2	mir-519d	31
mir-4725	3	mir-432	8	mir-519e	6
mir-4727	1	mir-433	12	mir-520a	1
mir-4728	2	mir-4417	7	mir-520d	1
mir-4732	2	mir-4423	3	mir-520f	1
mir-4742	2	mir-4425	1	mir-520g	10
mir-4747	2	mir-4430	2	mir-521	4
mir-4749	4	mir-4433	6	mir-523	2
mir-4750	5	mir-4433b	18	mir-524	2
mir-4753	2	mir-4482	2	mir-525	6
mir-4758	2	mir-449b	1	mir-532	230
mir-4762	1	mir-4500	97	mir-539	3
mir-4772	10	mir-450a	6	mir-542	13
mir-4783	4	mir-450b	5	mir-543	19
mir-483	29	mir-452	454	mir-548c	4
mir-484	2156	mir-4520b	2	mir-548d	1
mir-485	3	mir-4521	4	mir-548f	2

mir-4330	2	mir-503	2
mir-4417	26	mir-504	4
mir-4423	6	mir-505	36
mir-4433	4	mir-508	2
mir-4433b	4	mir-5100	6
mir-4472	1	mir-512	4
mir-4477a	1	mir-515	6
mir-4500	451	mir-517a	8
mir-450a	24	mir-517b	9
mir-450b	12	mir-518c	8
mir-452	399	mir-519a	2
mir-4521	32	mir-519b	2
mir-4524a	8	mir-519d	17
mir-4536	2	mir-519e	6
mir-454	743	mir-520f	2
mir-455	2638	mir-525	4
mir-4632	2	mir-532	150
mir-4635	5	mir-542	14
mir-4641	4	mir-543	4
mir-4646	2	mir-545	2
mir-466	3	mir-548a	2
mir-4660	2	mir-548f	2
mir-4662a	4	mir-548h	2
mir-4664	6	mir-548q	1
mir-4666b	1	mir-548t	4
mir-4667	2	mir-550a	2
mir-4668	13	mir-551a	4
mir-4677	2	mir-551b	4
mir-4687	4	mir-552	1
mir-4691	1	mir-5582	2
mir-4695	1	mir-5583	1
mir-4700	2	mir-5585	2
mir-4707	19	mir-5591	4

mir-4433	4
mir-4433b	1
mir-4445	1
mir-4450	4
mir-449b	1
mir-449c	2
mir-4500	2
mir-450b	2
mir-452	253
mir-4521	1
mir-4524a	1
mir-4524b	1
mir-4529	1
mir-454	81
mir-455	161
mir-4635	2
mir-4638	2
mir-4640	1
mir-4662a	2
mir-4677	1
mir-4680	2
mir-4690	9
mir-4697	2
mir-4700	1
mir-4707	5
mir-4708	5
mir-4709	1
mir-4712	2
mir-4713	4
mir-4728	8
mir-4731	2
mir-4745	4
mir-4746	1

mir-486	15	mir-454	421	mir-548j	2
mir-487a	1	mir-455	1048	mir-548o	2
mir-487b	78	mir-4632	4	mir-548t	6
mir-489	26	mir-4635	3	mir-551a	2
mir-492	1	mir-4640	7	mir-551b	2
mir-493	10	mir-4649	2	mir-5584	2
mir-494	34	mir-4652	2	mir-5585	4
mir-495	76	mir-4653	1	mir-5590	5
mir-497	270	mir-4655	1	mir-5739	1
mir-4999	4	mir-4659a	1	mir-574	883
mir-499a	2	mir-466	10	mir-576	11
mir-5001	6	mir-4662a	2	mir-582	6
mir-5003	3	mir-4664	12	mir-589	6
mir-5008	4	mir-4665	2	mir-590	75
mir-500a	30	mir-4666b	2	mir-598	8
mir-500b	3	mir-4668	6	mir-615	2
mir-501	42	mir-4676	2	mir-619	6
mir-502	127	mir-4677	8	mir-625	27
mir-503	92	mir-4685	6	mir-627	4
mir-504	2	mir-4687	6	mir-628	22
mir-505	369	mir-4690	5	mir-629	2
mir-508	2	mir-4700	9	mir-6503	6
mir-5100	24	mir-4707	12	mir-6504	2
mir-511	2	mir-4714	2	mir-6505	1
mir-512	8	mir-4723	2	mir-6512	1
mir-514a	2	mir-4728	2	mir-652	36
mir-515	10	mir-4738	2	mir-654	28
mir-516a	2	mir-4742	4	mir-655	2
mir-517a	18	mir-4745	2	mir-656	12
mir-517b	41	mir-4746	4	mir-660	97
mir-517c	12	mir-4747	6	mir-664a	63
mir-5188	2	mir-4753	4	mir-664b	4
mir-518c	14	mir-4764	1	mir-671	31

mir-4726	10	mir-574	390
mir-4727	2	mir-576	4
mir-4728	4	mir-579	2
mir-4735	1	mir-582	13
mir-4741	2	mir-584	2
mir-4742	6	mir-589	1
mir-4745	4	mir-590	185
mir-4746	2	mir-597	2
mir-4747	2	mir-6079	2
mir-4750	2	mir-615	14
mir-4753	3	mir-616	4
mir-4761	2	mir-624	2
mir-4762	3	mir-625	34
mir-4772	8	mir-628	14
mir-4778	1	mir-629	4
mir-4783	2	mir-642a	6
mir-4786	2	mir-6507	1
mir-4801	1	mir-6512	1
mir-4802	2	mir-6516	1
mir-483	67	mir-652	53
mir-484	3353	mir-654	12
mir-485	106	mir-655	4
mir-486	3	mir-656	4
mir-487a	46	mir-660	93
mir-487b	374	mir-664a	55
mir-489	17	mir-664b	8
mir-491	5	mir-665	4
mir-492	1	mir-671	48
mir-493	189	mir-6722	2
mir-494	145	mir-6723	1
mir-495	166	mir-6727	1
mir-497	240	mir-6740	4
mir-4999	1	mir-675	23

mir-4747	6
mir-4750	5
mir-4753	2
mir-4757	2
mir-4768	2
mir-4772	2
mir-4778	4
mir-4783	2
mir-4787	4
mir-4804	2
mir-483	8
mir-484	778
mir-485	20
mir-486	2
mir-487a	4
mir-487b	30
mir-489	2
mir-492	5
mir-493	17
mir-494	30
mir-495	92
mir-497	154
mir-499a	2
mir-5004	2
mir-5007	1
mir-5008	4
mir-500a	7
mir-501	27
mir-502	18
mir-503	59
mir-505	134
mir-508	1
mir-5088	4

mir-518e	2	mir-4772	8	mir-6716	2
mir-5196	28	mir-4778	1	mir-6722	8
mir-519a	6	mir-4782	1	mir-6731	2
mir-519b	5	mir-4783	49	mir-6740	1
mir-519d	14	mir-4787	2	mir-6746	1
mir-519e	6	mir-4789	1	mir-6749	32
mir-520c	9	mir-4790	1	mir-675	4
mir-520d	2	mir-4800	1	mir-6755	2
mir-520e	2	mir-4804	2	mir-6756	2
mir-520f	3	mir-483	46	mir-676	3
mir-520g	2	mir-484	4258	mir-6769a	4
mir-523	2	mir-485	22	mir-6769b	2
mir-524	6	mir-486	23	mir-6779	1
mir-525	4	mir-487a	26	mir-6786	2
mir-532	1410	mir-487b	207	mir-6791	4
mir-539	3	mir-489	140	mir-6793	5
mir-542	148	mir-490	2	mir-6798	2
mir-543	13	mir-491	12	mir-6799	2
mir-545	124	mir-493	60	mir-6810	2
mir-548a	8	mir-494	74	mir-6812	6
mir-548ah	4	mir-495	71	mir-6813	1
mir-548ar	2	mir-497	217	mir-6814	2
mir-548at	4	mir-499a	24	mir-6824	1
mir-548ay	4	mir-5001	3	mir-6836	2
mir-548az	1	mir-5003	2	mir-6848	2
mir-548b	12	mir-5008	1	mir-6850	4
mir-548e	5	mir-500a	84	mir-6851	2
mir-548h	4	mir-500b	5	mir-6858	2
mir-548q	4	mir-501	87	mir-6875	2
mir-548t	4	mir-5010	11	mir-6880	8
mir-548x	2	mir-502	139	mir-6891	2
mir-550a	18	mir-503	130	mir-6895	14
mir-551a	34	mir-504	4	mir-7	29

mir-499a	24	mir-6752	2
mir-5001	2	mir-6762	2
mir-5003	2	mir-6766	4
mir-5004	1	mir-6767	2
mir-5006	1	mir-6781	2
mir-5008	4	mir-6782	2
mir-500a	61	mir-6793	2
mir-500b	1	mir-6798	2
mir-501	42	mir-6803	6
mir-502	128	mir-6812	2
mir-503	334	mir-6825	1
mir-504	3	mir-6826	2
mir-5047	2	mir-6837	2
mir-505	646	mir-6842	2
mir-5088	3	mir-6844	2
mir-5100	8	mir-6850	2
mir-511	2	mir-6867	2
mir-512	28	mir-6869	1
mir-514b	2	mir-6889	6
mir-515	20	mir-6891	1
mir-516a	6	mir-6895	4
mir-516b	3	mir-7	82
mir-517a	50	mir-708	546
mir-517b	60	mir-7158	2
mir-517c	14	mir-744	64
mir-5187	6	mir-758	1
mir-518a	6	mir-766	18
mir-518c	20	mir-767	5
mir-518d	1	mir-769	4
mir-518e	6	mir-874	5
mir-5196	2	mir-877	10
mir-519a	264	mir-887	5
mir-519b	12	mir-889	4

mir-5095	2
mir-5100	26
mir-512	4
mir-513c	1
mir-514b	1
mir-515	4
mir-517a	38
mir-517b	43
mir-517c	10
mir-5189	6
mir-518c	14
mir-518e	2
mir-519a	2
mir-519d	22
mir-519e	4
mir-520a	2
mir-520f	4
mir-520g	6
mir-521	6
mir-522	4
mir-525	2
mir-532	254
mir-539	15
mir-542	19
mir-543	33
mir-544a	2
mir-545	33
mir-548a	2
mir-548ap	1
mir-548av	2
mir-548c	2
mir-548d	2
mir-548e	4

mir-551b	102	mir-5047	2	mir-708	155
mir-5571	2	mir-505	505	mir-7114	2
mir-5579	2	mir-506	21	mir-7157	1
mir-5581	12	mir-507	3	mir-744	36
mir-5582	2	mir-508	57	mir-766	21
mir-5584	2	mir-5087	2	mir-767	10
mir-5585	4	mir-5089	1	mir-769	4
mir-561	15	mir-509	18	mir-874	34
mir-5699	3	mir-510	7	mir-877	1
mir-570	4	mir-5100	42	mir-887	7
mir-574	1068	mir-511	8	mir-889	4
mir-576	20	mir-512	22	mir-92a	207
mir-579	29	mir-513a	1	mir-92b	41
mir-582	127	mir-513b	5	mir-93	720
mir-584	4	mir-514a	10	mir-937	1
mir-590	902	mir-514b	4	mir-940	8
mir-597	2	mir-515	12	mir-942	2
mir-598	171	mir-517a	40	mir-95	49
mir-615	10	mir-517b	70	mir-96	24
mir-616	13	mir-517c	9	mir-98	62
mir-619	7	mir-5187	6	mir-99a	1797
mir-624	16	mir-518c	40	mir-99b	220
mir-625	118	mir-518e	4		
mir-627	29	mir-5195	2		
mir-628	16	mir-5197	1		
mir-629	39	mir-519a	7		
mir-642a	15	mir-519b	8		
mir-642b	2	mir-519c	6		
mir-6502	2	mir-519d	23		
mir-6503	8	mir-519e	6		
mir-6507	2	mir-520a	2		
mir-6512	6	mir-520c	10		
mir-6513	6	mir-520d	2		

mir-519c	4	mir-92a	393
mir-519d	35	mir-92b	46
mir-519e	6	mir-93	1069
mir-520a	1	mir-937	1
mir-520c	3	mir-940	10
mir-520d	3	mir-95	27
mir-520e	1	mir-96	22
mir-520f	19	mir-98	109
mir-520g	10	mir-99a	1496
mir-521	66	mir-99b	293
mir-522	26		
mir-523	18		
mir-524	4		
mir-525	12		
mir-526b	6		
mir-532	2127		
mir-539	19		
mir-542	167		
mir-543	174		
mir-545	108		
mir-548aj	1		
mir-548aq	8		
mir-548ay	1		
mir-548az	2		
mir-548b	8		
mir-548d	3		
mir-548f	4		
mir-548h	1		
mir-548o	4		
mir-548t	2		
mir-548x	1		
mir-549a	3		
mir-550a	34		

mir-548h	1
mir-548j	1
mir-549a	44
mir-551a	7
mir-551b	12
mir-5571	1
mir-5584	1
mir-5585	7
mir-5591	2
mir-561	5
mir-574	201
mir-576	10
mir-579	11
mir-582	10
mir-584	5
mir-585	1
mir-590	463
mir-605	2
mir-615	14
mir-616	2
mir-619	27
mir-624	12
mir-625	13
mir-627	27
mir-628	2
mir-629	9
mir-6507	2
mir-6508	2
mir-6509	2
mir-6512	2
mir-652	151
mir-654	36
mir-655	11

mir-6514	15	mir-520e	1
mir-6515	18	mir-520f	15
mir-6516	2	mir-520g	20
mir-652	595	mir-523	6
mir-654	48	mir-524	2
mir-655	12	mir-525	5
mir-656	3	mir-526b	4
mir-660	400	mir-532	4540
mir-664a	115	mir-539	8
mir-664b	4	mir-542	50
mir-665	11	mir-543	45
mir-671	165	mir-545	48
mir-6720	3	mir-548a	4
mir-6723	1	mir-548ao	1
mir-6732	16	mir-548ap	1
mir-6735	4	mir-548aq	2
mir-6736	1	mir-548ar	2
mir-6741	5	mir-548at	1
mir-6743	12	mir-548av	2
mir-6746	1	mir-548d	2
mir-6747	8	mir-548e	2
mir-6749	8	mir-548o	1
mir-675	13	mir-548q	6
mir-6755	6	mir-548t	2
mir-676	2	mir-549a	5
mir-6763	2	mir-550a	11
mir-6768	10	mir-551a	4
mir-6771	4	mir-551b	63
mir-6779	1	mir-5571	4
mir-6781	4	mir-5572	1
mir-6782	2	mir-5579	1
mir-6786	1	mir-5581	4
mir-6791	12	mir-5584	2

mir-551a	14
mir-551b	40
mir-556	3
mir-5586	1
mir-5588	2
mir-5589	2
mir-561	4
mir-562	2
mir-5684	1
mir-5697	1
mir-5699	12
mir-570	18
mir-571	1
mir-574	2698
mir-576	44
mir-579	46
mir-582	110
mir-584	8
mir-589	10
mir-590	850
mir-597	1
mir-598	138
mir-605	2
mir-6084	2
mir-6090	2
mir-615	4
mir-616	10
mir-619	2
mir-624	17
mir-625	261
mir-627	103
mir-628	23
mir-629	54

mir-656	6
mir-660	222
mir-663b	6
mir-664a	15
mir-664b	2
mir-665	17
mir-671	96
mir-6720	2
mir-6721	2
mir-6726	3
mir-6730	1
mir-6731	1
mir-6732	2
mir-6734	4
mir-6738	1
mir-6740	3
mir-6742	1
mir-6743	8
mir-6746	3
mir-6748	2
mir-6749	6
mir-675	17
mir-6751	2
mir-6752	1
mir-6756	5
mir-6763	2
mir-6764	2
mir-6769b	4
mir-6771	1
mir-6776	2
mir-6779	1
mir-6781	2
mir-6786	1

mir-6795	4	mir-5585	45
mir-6796	14	mir-561	8
mir-6797	3	mir-5697	2
mir-6798	1	mir-5699	2
mir-6803	6	mir-570	7
mir-6808	2	mir-574	1874
mir-6809	8	mir-576	22
mir-6812	8	mir-5787	1
mir-6814	2	mir-579	15
mir-6819	2	mir-580	2
mir-6820	5	mir-582	56
mir-6822	6	mir-584	6
mir-6827	4	mir-589	15
mir-6830	11	mir-590	1280
mir-6837	1	mir-597	8
mir-6840	2	mir-598	102
mir-6848	1	mir-6079	1
mir-6849	8	mir-6090	2
mir-6853	6	mir-615	28
mir-6854	6	mir-616	15
mir-6858	2	mir-619	16
mir-6861	2	mir-624	9
mir-6870	1	mir-625	295
mir-6877	3	mir-627	12
mir-6879	1	mir-628	14
mir-6880	2	mir-629	18
mir-6881	3	mir-642a	4
mir-6883	1	mir-6499	1
mir-6885	2	mir-6503	27
mir-6891	1	mir-6507	1
mir-7	482	mir-6508	4
mir-708	845	mir-6512	5
mir-7108	2	mir-6513	6

mir-642a	34
mir-6500	4
mir-6503	32
mir-651	2
mir-6510	1
mir-6511b	6
mir-6513	8
mir-6514	14
mir-6515	6
mir-6516	5
mir-652	477
mir-654	254
mir-655	10
mir-656	14
mir-659	1
mir-660	522
mir-664a	173
mir-664b	28
mir-665	25
mir-671	336
mir-6716	6
mir-6720	4
mir-6722	2
mir-6723	2
mir-6731	2
mir-6733	2
mir-6734	12
mir-6740	1
mir-6742	1
mir-6743	3
mir-6746	1
mir-6747	4
mir-6748	2

mir-6789	2
mir-6791	10
mir-6793	2
mir-6796	4
mir-6797	2
mir-6798	3
mir-6800	2
mir-6803	4
mir-6804	2
mir-6805	1
mir-6812	2
mir-6813	4
mir-6814	2
mir-6815	2
mir-6819	4
mir-6820	6
mir-6821	2
mir-6822	3
mir-6824	2
mir-6825	6
mir-6826	2
mir-6827	2
mir-6830	1
mir-6838	3
mir-6844	2
mir-6850	4
mir-6851	1
mir-6858	5
mir-6865	3
mir-6868	4
mir-6870	2
mir-6874	1
mir-6876	6

mir-7109	2
mir-7111	4
mir-744	116
mir-758	6
mir-766	33
mir-767	26
mir-769	46
mir-7854	3
mir-873	3
mir-874	33
mir-875	2
mir-877	40
mir-887	15
mir-889	2
mir-891a	2
mir-891b	2
mir-9	8
mir-92a	1156
mir-92b	137
mir-93	4404
mir-937	3
mir-940	67
mir-95	407
mir-96	1217
mir-98	1468
mir-99a	27870
mir-99b	505

mir-6514	4
mir-6515	2
mir-6516	2
mir-652	857
mir-654	87
mir-655	20
mir-656	8
mir-657	1
mir-660	745
mir-663b	2
mir-664a	199
mir-664b	12
mir-665	21
mir-668	2
mir-671	205
mir-6720	20
mir-6722	2
mir-6723	1
mir-6726	1
mir-6727	1
mir-6732	6
mir-6734	2
mir-6735	2
mir-6736	1
mir-6739	4
mir-6742	2
mir-6743	4
mir-6748	2
mir-6749	32
mir-675	55
mir-6751	3
mir-6753	6
mir-6756	4

mir-6749	14
mir-675	28
mir-6750	2
mir-6755	10
mir-6756	4
mir-676	1
mir-6761	2
mir-6764	2
mir-6767	4
mir-6769b	2
mir-6772	2
mir-6779	1
mir-6780b	2
mir-6785	4
mir-6791	3
mir-6793	5
mir-6798	2
mir-6802	1
mir-6803	16
mir-6806	3
mir-6809	2
mir-6810	10
mir-6812	8
mir-6813	2
mir-6820	5
mir-6822	6
mir-6830	1
mir-6831	3
mir-6833	2
mir-6838	1
mir-6839	8
mir-6844	2
mir-6848	1

mir-6877	2
mir-6878	1
mir-6879	1
mir-6881	4
mir-6884	1
mir-6886	4
mir-6889	1
mir-6891	2
mir-6895	3
mir-7	138
mir-708	1046
mir-7107	11
mir-7110	2
mir-7114	1
mir-744	28
mir-766	12
mir-767	1
mir-769	24
mir-7847	3
mir-7851	4
mir-874	30
mir-876	2
mir-877	33
mir-885	4
mir-887	3
mir-891a	2
mir-92a	626
mir-92b	90
mir-93	1006
mir-937	6
mir-940	30
mir-942	3
mir-95	24

mir-6757	1
mir-6762	4
mir-6763	1
mir-6766	2
mir-6767	3
mir-6769b	2
mir-6774	4
mir-6775	2
mir-6779	2
mir-6780a	2
mir-6780b	10
mir-6781	2
mir-6783	2
mir-6787	2
mir-6789	6
mir-6791	28
mir-6793	5
mir-6795	6
mir-6797	2
mir-6798	3
mir-6799	2
mir-6801	2
mir-6803	10
mir-6805	1
mir-6807	2
mir-6809	2
mir-6811	1
mir-6812	14
mir-6814	1
mir-6815	1
mir-6819	2
mir-6821	4
mir-6822	1
mir-6850	2
mir-6851	5
mir-6871	1
mir-6876	1
mir-6878	10
mir-6886	3
mir-6887	2
mir-6894	12
mir-7	1551
mir-708	1313
mir-7107	2
mir-7110	2
mir-7111	4
mir-7114	4
mir-7151	3
mir-7152	1
mir-7162	2
mir-744	210
mir-758	24
mir-766	162
mir-767	2
mir-769	58
mir-7848	1
mir-7849	2
mir-873	1
mir-874	124
mir-875	2
mir-876	4
mir-877	31
mir-887	94
mir-889	6
mir-9	4
mir-92a	2003
mir-96	182
mir-98	312
mir-99a	3648
mir-99b	194

mir-6824	2
mir-6825	2
mir-6830	3
mir-6831	2
mir-6836	4
mir-6838	2
mir-6844	2
mir-6848	2
mir-6852	2
mir-6854	8
mir-6855	2
mir-6858	2
mir-6861	2
mir-6867	2
mir-6880	6
mir-6881	4
mir-6883	2
mir-6885	1
mir-6886	2
mir-6887	22
mir-6891	8
mir-6892	3
mir-6894	6
mir-6895	2
mir-7	327
mir-708	3363
mir-7110	3
mir-7111	2
mir-7155	1
mir-7156	2
mir-744	253
mir-758	7
mir-761	1

mir-92b	256
mir-93	5676
mir-937	2
mir-939	2
mir-940	85
mir-942	9
mir-95	253
mir-96	736
mir-98	2461
mir-99a	15635
mir-99b	533

mir-766	154
mir-767	193
mir-769	133
mir-770	2
mir-7847	1
mir-7848	1
mir-802	1
mir-873	4
mir-874	271
mir-877	55
mir-885	6
mir-887	84
mir-9	42
mir-92a	1801
mir-92b	462
mir-93	7076
mir-938	4
mir-939	3
mir-940	162
mir-942	2
mir-95	210
mir-96	731
mir-98	676
mir-99a	10599
mir-99b	1674
