# Supplementary material for: Small RNAs in metastatic and non-metastatic oral squamous cell carcinoma
Source: BMC Med Genomics. 2015 Jun 24;8:31. doi: 10.1186/s12920-015-0102-4 (PMC4479233; doi:10.1186/s12920-015-0102-4)
Supplement: Additional file3: — Complete set of detected mature miRNAs and correspondent read counts in metastatic tumor samples. We used miRBase v.20 as reference for miRNA identification. Read counts are raw numbers (not normalized). [file 12920_2015_102_MOESM3_ESM.pdf]

p0012		p0280		p374		p397		p0441		p0652		p0677		p1231		p1381		p1642	
**miR**	**Count**	**miR**	**Count**	**miR**	**Count**	**miR**	**Count**	**miR**	**Count**	**miR**	**Count**	**miR**	**Count**	**miR**	**Count**	**miR**	**Count**	**miR**	**Count**
let-7a	2138	let-7a	917	let-7a	327	let-7a	7815	let-7a	1839	let-7a	8301	let-7a	1281	let-7a	794	let-7a	160	let-7a	886
let-7b	3447	let-7b	1881	let-7b	437	let-7b	2669	let-7b	4386	let-7b	6239	let-7b	3744	let-7b	1292	let-7b	597	let-7b	2348
let-7c	506	let-7c	261	let-7c	55	let-7c	2804	let-7c	272	let-7c	303	let-7c	872	let-7c	195	let-7c	39	let-7c	136
let-7d	672	let-7d	539	let-7d	118	let-7d	3980	let-7d	573	let-7d	3178	let-7d	1161	let-7d	403	let-7d	69	let-7d	873
let-7e	300	let-7e	426	let-7e	53	let-7e	733	let-7e	266	let-7e	312	let-7e	475	let-7e	135	let-7e	16	let-7e	78
let-7f	1131	let-7f	439	let-7f	161	let-7f	8751	let-7f	1075	let-7f	3511	let-7f	573	let-7f	633	let-7f	84	let-7f	1490
let-7g	3783	let-7g	1210	let-7g	490	let-7g	25229	let-7g	2936	let-7g	9598	let-7g	3241	let-7g	1764	let-7g	224	let-7g	2237
let-7i	718	let-7i	244	let-7i	170	let-7i	14198	let-7i	1365	let-7i	1529	let-7i	807	let-7i	364	let-7i	340	let-7i	710
mir-1	36	mir-1	3	mir-1	44	mir-1	4	mir-1	332	mir-1	7	mir-1	280	mir-1	16	mir-1	52	mir-1	53
mir-100	1004	mir-100	780	mir-100	347	mir-100	4863	mir-100	932	mir-100	1833	mir-100	2259	mir-100	1452	mir-100	261	mir-100	786
mir-101	54	mir-101	4	mir-103a	631	mir-101	180	mir-101	19	mir-101	26	mir-101	74	mir-101	97	mir-101	28	mir-101	46
mir-103a	3235	mir-103a	3457	mir-106a	1	mir-103a	62416	mir-103a	5911	mir-103a	13591	mir-103a	6554	mir-103a	2532	mir-103a	360	mir-103a	5306
mir-106a	22	mir-106a	38	mir-106b	32	mir-106a	26	mir-106a	24	mir-105	4	mir-106a	17	mir-106a	6	mir-103b	1	mir-106a	4
mir-106b	280	mir-106b	247	mir-107	220	mir-106b	4604	mir-106b	349	mir-106a	28	mir-106b	215	mir-106b	170	mir-106b	13	mir-106b	270
mir-107	931	mir-107	637	mir-10a	20	mir-107	12430	mir-107	731	mir-106b	565	mir-107	923	mir-107	374	mir-107	156	mir-107	1173
mir-10a	87	mir-10a	64	mir-10b	68	mir-10a	679	mir-10a	272	mir-107	2015	mir-10a	66	mir-10a	67	mir-10a	16	mir-10a	183
mir-10b	551	mir-10b	66	mir-1225	2	mir-10b	953	mir-10b	266	mir-10a	67	mir-10b	494	mir-10b	283	mir-10b	135	mir-10b	1048
mir-1207	1	mir-1180	21	mir-1228	3	mir-1180	60	mir-1180	4	mir-10b	1196	mir-1178	1	mir-1182	1	mir-1247	2	mir-1179	2
mir-122	4	mir-122	6	mir-1247	3	mir-1185	40	mir-1182	1	mir-1180	12	mir-1180	4	mir-1197	2	mir-1250	1	mir-1180	6
mir-1227	8	mir-1226	6	mir-1250	1	mir-1197	2	mir-1185	6	mir-1185	4	mir-1182	1	mir-122	2	mir-125a	212	mir-1199	2
mir-1237	1	mir-1247	4	mir-125a	372	mir-122	4	mir-1225	2	mir-122	2	mir-1185	33	mir-1225	2	mir-125b	193	mir-1203	1
mir-1238	2	mir-1250	1	mir-125b	145	mir-1224	2	mir-1226	32	mir-1224	6	mir-122	6	mir-1247	8	mir-126	1039	mir-1224	5
mir-1247	22	mir-125a	3060	mir-126	1707	mir-1225	2	mir-1228	3	mir-1226	4	mir-1226	8	mir-125a	860	mir-1260b	6	mir-1226	10

mir-1250	2	mir-125b	1258	mir-1260b	30	mir-1226	13	mir-1234	2	mir-1227	10	mir-1228	1	mir-125b	480	mir-127	3	mir-1227	2
mir-1252	4	mir-126	822	mir-127	1	mir-1227	20	mir-1236	1	mir-1228	1	mir-1233	2	mir-126	5402	mir-1271	4	mir-1228	1
mir-125a	1600	mir-1260b	129	mir-1271	2	mir-1228	23	mir-1238	2	mir-1229	1	mir-1236	2	mir-1260b	30	mir-1277	62	mir-1229	2
mir-125b	1307	mir-127	2	mir-1273h	2	mir-1237	9	mir-1247	14	mir-1250	5	mir-1237	4	mir-127	1	mir-128	10	mir-1236	5
mir-126	11592	mir-1271	8	mir-1277	12	mir-1247	13	mir-1250	1	mir-1252	2	mir-1238	2	mir-1271	4	mir-1285	2	mir-1237	1
mir-1260b	74	mir-1277	34	mir-128	18	mir-1255a	4	mir-1255b	2	mir-125a	2210	mir-1245a	2	mir-1273d	1	mir-129	4	mir-1238	2
mir-127	4	mir-128	39	mir-129	4	mir-1256	16	mir-125a	2552	mir-125b	957	mir-1247	59	mir-1277	8	mir-1301	2	mir-1247	7
mir-1271	10	mir-1287	1	mir-1296	2	mir-125a	13759	mir-125b	778	mir-126	21824	mir-1252	2	mir-1278	1	mir-1307	12	mir-1255a	2
mir-1277	9	mir-129	4	mir-1307	7	mir-125b	15548	mir-126	6052	mir-1260b	210	mir-1255a	2	mir-128	8	mir-130a	267	mir-1255b	6
mir-128	47	mir-1292	4	mir-130a	418	mir-126	36458	mir-1260b	77	mir-1269b	2	mir-1256	4	mir-129	2	mir-130b	1430	mir-1256	2
mir-1285	4	mir-1296	4	mir-130b	145	mir-1260b	1798	mir-1262	2	mir-127	32	mir-125a	4092	mir-1296	2	mir-132	18	mir-125a	1278
mir-129	3	mir-1301	10	mir-132	28	mir-1262	2	mir-127	14	mir-1271	24	mir-125b	2814	mir-1301	13	mir-134	4	mir-125b	338
mir-1292	4	mir-1304	1	mir-1323	2	mir-1268a	5	mir-1271	12	mir-1277	19	mir-126	8443	mir-1306	2	mir-135b	24	mir-126	4608
mir-1296	6	mir-1306	16	mir-133a	10	mir-127	50	mir-1277	28	mir-128	87	mir-1260b	75	mir-1307	34	mir-136	27	mir-1260b	65
mir-1301	7	mir-1307	26	mir-133b	4	mir-1271	98	mir-1278	2	mir-1285	6	mir-127	169	mir-130a	1037	mir-138	5	mir-1268a	2
mir-1304	2	mir-130a	840	mir-134	6	mir-1273d	2	mir-128	61	mir-1287	6	mir-1271	8	mir-130b	170	mir-139	22	mir-127	18
mir-1306	10	mir-130b	1160	mir-1343	1	mir-1273g	17	mir-1285	4	mir-129	3	mir-1273g	2	mir-132	32	mir-140	80	mir-1271	7
mir-1307	9	mir-132	38	mir-135a	2	mir-1273h	1	mir-1287	2	mir-1292	3	mir-1273h	2	mir-133a	2	mir-141	22	mir-1273d	1
mir-130a	1181	mir-133a	2	mir-135b	13	mir-1277	46	mir-129	1	mir-1295a	2	mir-1277	19	mir-134	2	mir-142	169	mir-1273g	14
mir-130b	1146	mir-134	18	mir-136	13	mir-1278	2	mir-1292	8	mir-1296	8	mir-128	72	mir-135a	11	mir-143	223	mir-1273h	4
mir-132	36	mir-135a	2	mir-139	22	mir-128	241	mir-1295a	2	mir-1301	10	mir-1285	3	mir-135b	207	mir-144	39	mir-1277	9
mir-	2	mir-	359	mir-140	118	mir-	38	mir-	2	mir-	26	mir-129	6	mir-	58	mir-	383	mir-	104

133a		135b				1285		1296		1306				136		145		128	
mir-134	10	mir-136	8	mir-141	31	mir-1287	14	mir-1301	25	mir-1307	47	mir-1296	12	mir-138	39	mir-146a	135	mir-1285	32
mir-1343	1	mir-138	7	mir-142	142	mir-1288	1	mir-1304	8	mir-130a	5642	mir-1301	27	mir-139	14	mir-146b	20	mir-1287	2
mir-135a	6	mir-139	29	mir-143	152	mir-129	9	mir-1306	17	mir-130b	917	mir-1304	1	mir-140	203	mir-147b	1	mir-1288	1
mir-135b	200	mir-140	171	mir-144	26	mir-1292	8	mir-1307	28	mir-132	87	mir-1306	19	mir-141	331	mir-148a	1645	mir-129	2
mir-136	46	mir-141	163	mir-145	431	mir-1295a	19	mir-130a	2875	mir-1323	4	mir-1307	140	mir-142	984	mir-148b	18	mir-1290	6
mir-138	38	mir-142	89	mir-146a	10	mir-1296	19	mir-130b	1538	mir-133a	2	mir-130a	1532	mir-143	674	mir-149	8	mir-1292	3
mir-139	147	mir-143	180	mir-146b	20	mir-1301	69	mir-132	162	mir-134	4	mir-130b	765	mir-144	31	mir-150	148	mir-1296	9
mir-140	730	mir-144	18	mir-147b	2	mir-1304	13	mir-1323	2	mir-135b	846	mir-132	137	mir-145	886	mir-151a	45	mir-1301	7
mir-141	159	mir-145	2282	mir-148a	109	mir-1306	133	mir-133a	40	mir-136	16	mir-133a	190	mir-146a	356	mir-151b	2	mir-1304	4
mir-142	1165	mir-146a	14	mir-148b	12	mir-1307	300	mir-133b	15	mir-137	1	mir-133b	120	mir-146b	89	mir-152	27	mir-1306	4
mir-143	1357	mir-146b	32	mir-149	70	mir-130a	31126	mir-134	35	mir-138	179	mir-134	51	mir-147b	8	mir-1537	5	mir-1307	37
mir-144	362	mir-147b	2	mir-150	410	mir-130b	7419	mir-1343	2	mir-139	155	mir-1343	6	mir-148a	1657	mir-154	18	mir-130a	2863
mir-145	3505	mir-148a	212	mir-151a	47	mir-132	254	mir-135b	186	mir-140	675	mir-135a	5	mir-148b	100	mir-155	61	mir-130b	1018
mir-146a	174	mir-148b	22	mir-151b	4	mir-1323	8	mir-136	18	mir-141	569	mir-135b	60	mir-149	84	mir-15a	180	mir-132	16
mir-146b	120	mir-149	445	mir-152	23	mir-133a	2	mir-138	51	mir-142	883	mir-136	201	mir-150	460	mir-15b	106	mir-1323	2
mir-148a	2909	mir-150	310	mir-154	6	mir-134	14	mir-139	40	mir-143	1185	mir-138	25	mir-151a	151	mir-16	388	mir-133b	4
mir-148b	138	mir-151a	253	mir-155	10	mir-1343	13	mir-140	504	mir-144	68	mir-139	323	mir-151b	3	mir-17	366	mir-134	6
mir-149	151	mir-151b	10	mir-15a	65	mir-135a	19	mir-141	469	mir-145	5110	mir-140	1592	mir-152	128	mir-181a	96	mir-1343	3
mir-150	2009	mir-152	63	mir-15b	100	mir-135b	13887	mir-142	823	mir-146a	189	mir-141	47	mir-153	5	mir-181b	100	mir-135a	2
mir-151a	387	mir-154	10	mir-16	348	mir-136	159	mir-143	955	mir-146b	221	mir-142	1014	mir-1537	5	mir-181c	23	mir-135b	422
mir-151b	8	mir-155	16	mir-17	309	mir-138	580	mir-144	268	mir-147b	16	mir-143	2246	mir-154	8	mir-181d	36	mir-136	42

mir-152	210	mir-15a	80	mir-181a	120	mir-139	62	mir-145	3039	mir-148a	1952	mir-144	326	mir-155	61	mir-182	24	mir-137	2
mir-154	18	mir-15b	1277	mir-181b	106	mir-140	3001	mir-146a	488	mir-148b	118	mir-145	8085	mir-15a	357	mir-183	6	mir-138	32
mir-155	137	mir-16	1500	mir-181c	9	mir-141	2672	mir-146b	158	mir-149	537	mir-146a	138	mir-15b	481	mir-185	52	mir-139	15
mir-15a	465	mir-17	420	mir-181d	14	mir-142	4818	mir-147b	2	mir-150	1265	mir-146b	558	mir-16	2535	mir-186	54	mir-140	323
mir-15b	1284	mir-181a	508	mir-182	17	mir-143	3494	mir-148a	626	mir-151a	798	mir-147b	1	mir-17	748	mir-187	7	mir-141	1245
mir-16	3046	mir-181b	102	mir-1827	2	mir-144	646	mir-148b	89	mir-151b	11	mir-148a	1384	mir-181a	122	mir-188	8	mir-142	378
mir-17	1435	mir-181c	31	mir-183	9	mir-145	10190	mir-149	255	mir-152	499	mir-148b	87	mir-181b	24	mir-18a	6	mir-143	585
mir-181a	506	mir-181d	37	mir-185	8	mir-1468	11	mir-150	1538	mir-1537	3	mir-149	54	mir-181c	94	mir-18b	1	mir-144	86
mir-181b	52	mir-182	582	mir-186	17	mir-1469	2	mir-151a	377	mir-154	16	mir-150	1390	mir-181d	17	mir-190a	1	mir-145	1894
mir-181c	56	mir-183	167	mir-187	8	mir-146a	936	mir-151b	2	mir-155	73	mir-151a	360	mir-182	169	mir-191	188	mir-1468	2
mir-181d	7	mir-184	2	mir-188	10	mir-146b	603	mir-152	223	mir-15a	844	mir-151b	1	mir-183	57	mir-192	15	mir-146a	107
mir-182	265	mir-185	120	mir-18a	18	mir-147b	68	mir-153	2	mir-15b	2508	mir-152	360	mir-185	171	mir-193a	3	mir-146b	60
mir-183	79	mir-186	52	mir-18b	6	mir-148a	8586	mir-154	37	mir-16	10919	mir-153	2	mir-186	168	mir-193b	238	mir-147b	4
mir-185	445	mir-187	39	mir-1908	2	mir-148b	751	mir-155	95	mir-17	7775	mir-1537	6	mir-187	22	mir-194	3	mir-148a	2902
mir-186	297	mir-188	2	mir-1909	2	mir-149	8104	mir-15a	413	mir-181a	660	mir-154	66	mir-188	16	mir-195	34	mir-148b	245
mir-187	22	mir-18a	44	mir-190a	2	mir-150	4276	mir-15b	1202	mir-181b	44	mir-155	89	mir-18a	106	mir-197	15	mir-149	464
mir-188	31	mir-18b	9	mir-191	304	mir-151a	2018	mir-16	4028	mir-181c	87	mir-15a	515	mir-18b	2	mir-199a	410	mir-150	131
mir-18a	109	mir-190a	2	mir-192	15	mir-151b	34	mir-17	1877	mir-181d	2	mir-15b	794	mir-190a	2	mir-199b	1366	mir-151a	346
mir-18b	14	mir-191	992	mir-193b	56	mir-152	315	mir-181a	1012	mir-182	1480	mir-16	6025	mir-191	761	mir-19a	172	mir-151b	4
mir-1909	18	mir-192	53	mir-195	59	mir-153	1	mir-181b	113	mir-183	291	mir-17	1002	mir-192	53	mir-19b	170	mir-152	374
mir-190a	33	mir-193a	60	mir-197	15	mir-1537	5	mir-181c	90	mir-184	4	mir-181a	841	mir-193a	64	mir-200a	65	mir-1537	1
mir-	1320	mir-	774	mir-	218	mir-154	62	mir-	64	mir-185	1175	mir-	71	mir-	178	mir-	51	mir-	3

191		193b		199a				181d				181b		193b		200b		154	
mir-1911	1	mir-194	4	mir-199b	854	mir-155	218	mir-182	221	mir-186	321	mir-181c	67	mir-194	4	mir-200c	51	mir-155	22
mir-1913	2	mir-195	64	mir-19a	10	mir-15a	2312	mir-183	67	mir-187	107	mir-181d	27	mir-195	204	mir-203a	711	mir-15a	473
mir-1914	1	mir-196a	8	mir-19b	56	mir-15b	8329	mir-185	452	mir-188	43	mir-182	32	mir-196a	4	mir-204	10	mir-15b	1396
mir-192	140	mir-196b	7	mir-200a	71	mir-16	77031	mir-186	227	mir-18a	752	mir-1827	2	mir-196b	4	mir-205	3170	mir-16	6707
mir-193a	79	mir-197	139	mir-200b	68	mir-17	7258	mir-187	16	mir-18b	12	mir-183	13	mir-197	61	mir-206	57	mir-17	1189
mir-193b	474	mir-1976	2	mir-200c	38	mir-181a	1247	mir-188	16	mir-190a	10	mir-185	1057	mir-199a	1627	mir-20a	76	mir-181a	250
mir-194	4	mir-199a	458	mir-203a	433	mir-181b	168	mir-18a	227	mir-191	2939	mir-186	393	mir-199b	4067	mir-20b	4	mir-181b	50
mir-195	317	mir-199b	2009	mir-204	7	mir-181c	908	mir-18b	14	mir-1910	8	mir-187	12	mir-19a	593	mir-21	7734	mir-181c	86
mir-196a	18	mir-19a	68	mir-205	2524	mir-181d	84	mir-1909	8	mir-192	231	mir-188	39	mir-19b	955	mir-210	89	mir-181d	10
mir-196b	2	mir-19b	172	mir-206	75	mir-182	5432	mir-190a	13	mir-193a	183	mir-18a	172	mir-200a	725	mir-212	2	mir-182	365
mir-197	98	mir-200a	377	mir-20a	76	mir-1827	2	mir-191	1432	mir-193b	780	mir-18b	9	mir-200b	358	mir-214	169	mir-183	160
mir-199a	1851	mir-200b	392	mir-20b	4	mir-183	1006	mir-1910	4	mir-195	757	mir-1908	2	mir-200c	194	mir-218	2	mir-184	4
mir-199b	7667	mir-200c	1400	mir-21	2690	mir-184	184	mir-192	86	mir-196a	8	mir-190a	2	mir-203a	4150	mir-22	189	mir-185	219
mir-19a	805	mir-203a	2232	mir-210	27	mir-185	1711	mir-193a	58	mir-196b	83	mir-190b	1	mir-203b	3	mir-221	490	mir-186	123
mir-19b	1211	mir-203b	2	mir-2110	4	mir-186	1380	mir-193b	1163	mir-197	231	mir-191	2798	mir-204	8	mir-222	45	mir-187	98
mir-200a	433	mir-204	345	mir-214	227	mir-187	161	mir-194	4	mir-199a	2580	mir-1911	1	mir-205	18853	mir-223	231	mir-188	10
mir-200b	398	mir-205	21857	mir-215	2	mir-188	199	mir-195	205	mir-199b	5980	mir-192	58	mir-206	12	mir-224	42	mir-18a	103
mir-200c	347	mir-20a	128	mir-218	8	mir-18a	791	mir-196a	62	mir-19a	1693	mir-193a	229	mir-20a	347	mir-2355	2	mir-18b	15
mir-202	5	mir-20b	124	mir-22	88	mir-18b	15	mir-196b	14	mir-19b	3511	mir-193b	496	mir-20b	8	mir-23a	1139	mir-1908	4
mir-203a	12687	mir-21	6002	mir-221	114	mir-1908	7	mir-197	123	mir-200a	1808	mir-194	2	mir-21	15270	mir-23b	267	mir-1909	10
mir-203b	13	mir-210	235	mir-222	17	mir-1909	2	mir-1976	2	mir-200b	1392	mir-195	470	mir-210	228	mir-23c	943	mir-190a	21

mir-204	42	mir-2110	2	mir-223	383	mir-190a	24	mir-199a	1956	mir-200c	512	mir-196a	2	mir-211	4	mir-24	384	mir-190b	1
mir-205	9311	mir-2116	2	mir-224	39	mir-191	27167	mir-199b	5569	mir-203a	57151	mir-196b	4	mir-2110	2	mir-25	72	mir-191	1417
mir-208a	3	mir-214	720	mir-2355	1	mir-1910	7	mir-19a	715	mir-203b	8	mir-197	364	mir-214	365	mir-26a	155	mir-1910	4
mir-20a	614	mir-215	2	mir-23a	870	mir-1914	1	mir-19b	1089	mir-204	38	mir-1976	2	mir-218	42	mir-26b	99	mir-1914	3
mir-20b	80	mir-217	2	mir-23b	301	mir-1915	1	mir-200a	250	mir-205	87204	mir-199a	4652	mir-22	477	mir-27a	478	mir-1915	3
mir-21	21763	mir-218	26	mir-23c	161	mir-192	14328	mir-200b	244	mir-206	4	mir-199b	9129	mir-221	867	mir-27b	154	mir-192	150
mir-210	181	mir-22	110	mir-24	698	mir-193a	676	mir-200c	1099	mir-20a	3495	mir-19a	415	mir-222	283	mir-28	36	mir-193a	37
mir-2110	2	mir-221	1242	mir-25	76	mir-193b	4439	mir-203a	6685	mir-20b	114	mir-19b	846	mir-223	1585	mir-299	5	mir-193b	310
mir-214	995	mir-222	192	mir-26a	486	mir-194	40	mir-204	10	mir-21	40306	mir-200a	117	mir-224	88	mir-29a	1127	mir-194	24
mir-215	1	mir-223	631	mir-26b	108	mir-195	3113	mir-205	30107	mir-210	2150	mir-200b	76	mir-2278	2	mir-29b	198	mir-195	112
mir-218	56	mir-224	137	mir-27a	385	mir-196a	111	mir-206	821	mir-2110	2	mir-200c	99	mir-23a	3391	mir-29c	898	mir-196a	8
mir-219a	2	mir-2277	8	mir-27b	148	mir-196b	97	mir-208a	3	mir-2116	8	mir-203a	583	mir-23b	1103	mir-301a	6	mir-196b	114
mir-22	594	mir-2278	2	mir-28	58	mir-197	1044	mir-20a	931	mir-214	1334	mir-203b	2	mir-23c	33	mir-30a	47	mir-197	65
mir-221	895	mir-2355	6	mir-299	2	mir-1976	7	mir-20b	112	mir-215	2	mir-204	8	mir-24	3231	mir-30b	82	mir-1976	4
mir-222	289	mir-23a	2410	mir-29a	896	mir-199a	5598	mir-21	39371	mir-216a	1	mir-205	2040	mir-25	314	mir-30c	14	mir-198	4
mir-223	3857	mir-23b	2398	mir-29b	98	mir-199b	11710	mir-210	1189	mir-218	22	mir-206	1095	mir-2682	1	mir-30d	96	mir-199a	1084
mir-224	333	mir-23c	232	mir-29c	474	mir-19a	3349	mir-2110	9	mir-219a	3	mir-208b	4	mir-26a	1540	mir-30e	106	mir-199b	2855
mir-2355	2	mir-24	3900	mir-301a	6	mir-19b	4619	mir-2116	6	mir-219b	2	mir-20a	219	mir-26b	572	mir-31	246	mir-19a	1546
mir-23a	5272	mir-25	429	mir-302a	1	mir-200a	6769	mir-212	14	mir-22	1658	mir-20b	35	mir-27a	2038	mir-3129	2	mir-19b	2539
mir-23b	2262	mir-26a	1891	mir-30a	44	mir-200b	2836	mir-214	1748	mir-221	1443	mir-21	41089	mir-27b	603	mir-3150b	1	mir-200a	1851
mir-23c	129	mir-26b	195	mir-30b	105	mir-200c	722	mir-218	20	mir-222	275	mir-210	319	mir-28	76	mir-3187	2	mir-200b	612
mir-24	5067	mir-27a	531	mir-30c	44	mir-202	2	mir-	2	mir-223	4169	mir-	2	mir-	3770	mir-	1	mir-	636

								219a				2110		29a		3194		200c	
mir-25	456	mir-27b	412	mir-30d	85	mir-203a	4013	mir-22	582	mir-224	2080	mir-2116	1	mir-29b	1266	mir-32	4	mir-202	1
mir-26a	4162	mir-28	301	mir-30e	39	mir-203b	12	mir-221	1505	mir-2277	5	mir-212	8	mir-29c	3692	mir-320b	2	mir-203a	2140
mir-26b	617	mir-296	117	mir-31	167	mir-204	6552	mir-222	274	mir-2278	5	mir-214	5681	mir-301a	106	mir-323a	7	mir-203b	6
mir-27a	2272	mir-29a	2653	mir-3186	2	mir-205	125620	mir-223	16394	mir-23a	12207	mir-218	113	mir-301b	1	mir-324	16	mir-204	171
mir-27b	817	mir-29b	121	mir-32	6	mir-206	8	mir-224	331	mir-23b	4666	mir-219b	2	mir-3064	1	mir-326	2	mir-205	29816
mir-28	209	mir-29c	765	mir-324	15	mir-20a	1515	mir-2277	2	mir-23c	78	mir-22	1769	mir-3065	11	mir-328	3	mir-2054	2
mir-296	22	mir-301a	87	mir-326	3	mir-20b	77	mir-2278	2	mir-24	17256	mir-221	1191	mir-30a	315	mir-331	10	mir-206	19
mir-299	25	mir-301b	8	mir-328	2	mir-21	67716	mir-2355	1	mir-25	1283	mir-222	314	mir-30b	322	mir-335	10	mir-208b	7
mir-29a	6854	mir-3065	4	mir-329	2	mir-210	2469	mir-23a	9969	mir-26a	5421	mir-223	5879	mir-30c	100	mir-337	8	mir-20a	279
mir-29b	1142	mir-3074	1	mir-331	16	mir-211	48	mir-23b	1052	mir-26b	715	mir-224	76	mir-30d	347	mir-338	28	mir-20b	12
mir-29c	4155	mir-30a	52	mir-335	4	mir-2110	2	mir-23c	315	mir-27a	5203	mir-2277	3	mir-30e	515	mir-339	34	mir-21	26166
mir-301a	163	mir-30b	236	mir-337	4	mir-2114	2	mir-24	7876	mir-27b	1331	mir-2278	2	mir-31	3243	mir-33a	6	mir-210	2788
mir-301b	6	mir-30c	76	mir-338	8	mir-2116	6	mir-25	567	mir-28	434	mir-2355	3	mir-3127	1	mir-340	4	mir-211	2
mir-3064	2	mir-30d	240	mir-339	25	mir-212	11	mir-26a	2522	mir-296	33	mir-23a	7233	mir-3150a	1	mir-342	129	mir-2110	6
mir-3065	11	mir-30e	115	mir-33a	6	mir-214	1920	mir-26b	571	mir-299	10	mir-23b	2098	mir-3152	2	mir-345	10	mir-2114	2
mir-30a	213	mir-31	3085	mir-33b	4	mir-215	15	mir-27a	3248	mir-29a	13761	mir-23c	61	mir-32	17	mir-34a	263	mir-2116	4
mir-30b	1085	mir-3120	2	mir-340	5	mir-217	8	mir-27b	385	mir-29b	1712	mir-24	7054	mir-320b	2	mir-34b	2	mir-212	5
mir-30c	300	mir-3126	2	mir-342	53	mir-218	1112	mir-28	314	mir-29c	4591	mir-25	434	mir-323a	5	mir-34c	9	mir-214	189
mir-30d	507	mir-3127	1	mir-345	18	mir-219a	1	mir-296	23	mir-301a	217	mir-26a	5394	mir-324	33	mir-3607	17	mir-215	87
mir-30e	554	mir-3145	6	mir-34a	88	mir-219b	10	mir-299	12	mir-301b	19	mir-26b	564	mir-326	14	mir-3609	1	mir-216a	2
mir-31	1471	mir-3150b	2	mir-34b	4	mir-22	3255	mir-29a	5394	mir-3065	74	mir-27a	3011	mir-328	14	mir-361	46	mir-216b	3

mir-3117	1	mir-3152	4	mir-34c	4	mir-221	6423	mir-29b	758	mir-30a	139	mir-27b	649	mir-330	5	mir-3613	8	mir-217	2
mir-3126	2	mir-3180	2	mir-3605	2	mir-222	3488	mir-29c	1607	mir-30b	3193	mir-28	113	mir-331	74	mir-3614	2	mir-218	37
mir-3140	2	mir-320b	8	mir-3607	33	mir-223	26470	mir-301a	99	mir-30c	257	mir-296	13	mir-335	166	mir-362	15	mir-219a	6
mir-3141	1	mir-323a	7	mir-361	31	mir-224	1186	mir-302a	2	mir-30d	1426	mir-299	75	mir-337	20	mir-365a	26	mir-219b	1
mir-3145	2	mir-324	49	mir-3613	23	mir-2276	2	mir-3065	7	mir-30e	916	mir-29a	9687	mir-338	48	mir-365b	44	mir-22	545
mir-3152	8	mir-326	12	mir-362	10	mir-2277	2	mir-30a	423	mir-31	4419	mir-29b	1742	mir-339	96	mir-369	24	mir-221	4154
mir-3157	1	mir-328	58	mir-365a	10	mir-2278	6	mir-30b	473	mir-3117	2	mir-29c	4534	mir-33a	10	mir-374a	10	mir-222	821
mir-3161	2	mir-329	2	mir-365b	31	mir-2355	14	mir-30c	106	mir-3120	2	mir-301a	88	mir-33b	10	mir-374b	16	mir-223	1819
mir-3191	1	mir-330	26	mir-3667	1	mir-23a	35647	mir-30d	505	mir-3126	1	mir-301b	7	mir-340	22	mir-375	1	mir-224	1306
mir-32	32	mir-331	146	mir-369	5	mir-23b	35250	mir-30e	430	mir-3136	3	mir-3065	1	mir-342	289	mir-376a	8	mir-2277	2
mir-320b	8	mir-335	44	mir-373	2	mir-23c	438	mir-31	3961	mir-3150b	2	mir-30a	236	mir-345	39	mir-376b	2	mir-2278	2
mir-323a	13	mir-337	8	mir-374a	5	mir-24	30615	mir-3129	1	mir-3158	2	mir-30b	1397	mir-34a	573	mir-376c	133	mir-2355	3
mir-324	32	mir-338	28	mir-374b	16	mir-2467	2	mir-3173	6	mir-3173	4	mir-30c	196	mir-34b	176	mir-377	2	mir-23a	5307
mir-326	16	mir-339	170	mir-376a	6	mir-25	13266	mir-3186	3	mir-32	32	mir-30d	857	mir-34c	168	mir-378a	17	mir-23b	1637
mir-328	18	mir-33a	10	mir-376b	8	mir-26a	18552	mir-32	12	mir-320b	4	mir-30e	615	mir-3607	49	mir-379	5	mir-23c	297
mir-329	4	mir-33b	2	mir-376c	207	mir-26b	2171	mir-320b	14	mir-323a	9	mir-31	1321	mir-361	104	mir-381	10	mir-24	3793
mir-330	5	mir-340	18	mir-378a	25	mir-27a	16735	mir-323a	29	mir-323b	8	mir-3115	2	mir-3613	88	mir-382	5	mir-2467	34
mir-331	139	mir-342	347	mir-379	10	mir-27b	8014	mir-323b	4	mir-324	286	mir-3117	3	mir-362	50	mir-3944	1	mir-25	1189
mir-335	91	mir-345	140	mir-3928	1	mir-28	951	mir-324	63	mir-326	60	mir-3129	1	mir-363	8	mir-409	9	mir-26a	969
mir-337	33	mir-34a	171	mir-3960	2	mir-296	1140	mir-326	37	mir-328	32	mir-3140	2	mir-3659	2	mir-410	2	mir-26b	514
mir-338	50	mir-34b	5	mir-409	9	mir-299	26	mir-328	68	mir-329	4	mir-3144	2	mir-365a	40	mir-423	28	mir-27a	2059
mir-	188	mir-34c	22	mir-410	2	mir-29a	59638	mir-	10	mir-330	4	mir-	2	mir-	63	mir-	348	mir-	598

339								329				3157		365b		424		27b	
mir-33a	32	mir-3607	33	mir-411	1	mir-29b	7020	mir-330	8	mir-331	132	mir-3160	4	mir-369	8	mir-425	99	mir-28	169
mir-33b	2	mir-361	102	mir-423	8	mir-29c	25481	mir-331	101	mir-335	106	mir-3177	2	mir-374a	32	mir-4270	4	mir-2861	1
mir-340	28	mir-3613	4	mir-424	271	mir-301a	1811	mir-335	53	mir-337	27	mir-3189	1	mir-374b	70	mir-429	12	mir-296	6
mir-342	550	mir-362	4	mir-425	58	mir-301b	287	mir-337	26	mir-338	28	mir-3194	4	mir-375	12	mir-4300	2	mir-298	4
mir-345	62	mir-363	41	mir-429	14	mir-3064	5	mir-338	22	mir-339	856	mir-32	9	mir-376a	20	mir-432	2	mir-299	2
mir-34a	446	mir-3659	2	mir-4300	4	mir-3065	39	mir-339	236	mir-33a	122	mir-320b	9	mir-376b	12	mir-4417	20	mir-29a	3417
mir-34b	17	mir-365a	76	mir-4433	2	mir-3074	16	mir-33a	34	mir-33b	9	mir-323a	18	mir-376c	162	mir-4433	2	mir-29b	974
mir-34c	38	mir-365b	141	mir-4500	6	mir-30a	2641	mir-340	49	mir-340	33	mir-323b	21	mir-378a	100	mir-4500	6	mir-29c	3401
mir-3605	6	mir-367	1	mir-452	2	mir-30b	4363	mir-342	684	mir-342	1674	mir-324	185	mir-379	2	mir-452	7	mir-301a	242
mir-3607	14	mir-3679	2	mir-4521	2	mir-30c	1259	mir-345	96	mir-345	326	mir-326	357	mir-381	5	mir-454	7	mir-301b	19
mir-3609	1	mir-369	2	mir-454	6	mir-30d	1394	mir-34a	1371	mir-34a	2106	mir-328	68	mir-382	10	mir-455	6	mir-302a	3
mir-361	182	mir-370	4	mir-455	21	mir-30e	3530	mir-34b	37	mir-34b	81	mir-329	8	mir-3934	1	mir-4738	2	mir-302d	1
mir-3613	47	mir-374a	12	mir-466	2	mir-31	15782	mir-34c	66	mir-34c	251	mir-330	11	mir-3960	10	mir-4758	1	mir-302e	3
mir-362	65	mir-374b	40	mir-4684	1	mir-3117	5	mir-3607	9	mir-3607	28	mir-331	270	mir-409	12	mir-4777	1	mir-3065	1
mir-363	32	mir-376a	6	mir-4745	2	mir-3120	4	mir-3609	7	mir-3609	1	mir-335	24	mir-423	47	mir-4779	1	mir-3074	1
mir-365a	76	mir-376b	16	mir-4747	1	mir-3124	2	mir-361	204	mir-361	413	mir-337	133	mir-424	711	mir-4783	2	mir-30a	178
mir-365b	129	mir-376c	97	mir-4753	4	mir-3135a	6	mir-3613	93	mir-3613	137	mir-338	78	mir-425	373	mir-483	3	mir-30b	839
mir-3665	1	mir-377	1	mir-4763	2	mir-3136	4	mir-3617	8	mir-3614	2	mir-339	388	mir-429	85	mir-484	35	mir-30c	102
mir-3667	1	mir-378a	114	mir-4778	2	mir-3140	2	mir-362	14	mir-3619	1	mir-33a	36	mir-4293	1	mir-487a	2	mir-30d	545
mir-3675	1	mir-379	2	mir-4783	8	mir-3144	8	mir-3620	6	mir-362	73	mir-33b	8	mir-4417	22	mir-487b	8	mir-30e	526
mir-3678	2	mir-381	15	mir-4800	1	mir-3145	15	mir-363	37	mir-3620	6	mir-340	115	mir-4423	2	mir-494	7	mir-31	1387

mir-3679	4	mir-382	12	mir-483	6	mir-3150a	3	mir-365a	134	mir-363	36	mir-342	1056	mir-449b	2	mir-495	20	mir-3120	2
mir-369	26	mir-3940	2	mir-484	94	mir-3158	2	mir-365b	273	mir-365a	142	mir-345	129	mir-449c	2	mir-497	14	mir-3122	1
mir-370	6	mir-409	14	mir-485	4	mir-3161	2	mir-3667	1	mir-365b	274	mir-346	2	mir-4500	31	mir-499a	4	mir-3126	1
mir-371a	1	mir-411	4	mir-487a	2	mir-3162	4	mir-3675	1	mir-369	7	mir-34a	3236	mir-452	27	mir-5007	1	mir-3131	4
mir-371b	1	mir-412	2	mir-487b	5	mir-3173	11	mir-3679	2	mir-3691	1	mir-34b	19	mir-454	28	mir-5008	4	mir-3135a	4
mir-374a	87	mir-423	237	mir-489	2	mir-3177	3	mir-369	30	mir-370	1	mir-34c	30	mir-455	73	mir-500a	2	mir-3140	1
mir-374b	150	mir-424	292	mir-493	4	mir-3180	2	mir-370	6	mir-371a	2	mir-3591	2	mir-4646	2	mir-501	8	mir-3144	28
mir-374c	3	mir-425	298	mir-494	2	mir-3187	2	mir-371b	1	mir-371b	1	mir-3605	1	mir-4649	4	mir-502	8	mir-3150b	2
mir-375	5	mir-429	61	mir-495	15	mir-3194	14	mir-373	2	mir-374a	56	mir-3607	7	mir-4676	2	mir-504	2	mir-3155a	2
mir-376a	95	mir-4293	1	mir-497	17	mir-32	244	mir-374a	27	mir-374b	146	mir-361	275	mir-4685	1	mir-505	10	mir-3157	2
mir-376b	35	mir-4300	3	mir-499a	4	mir-3200	8	mir-374b	52	mir-374c	2	mir-3613	167	mir-4697	1	mir-5100	3	mir-3159	4
mir-376c	455	mir-431	2	mir-5008	4	mir-320b	4	mir-375	3	mir-376a	37	mir-3614	6	mir-4704	2	mir-512	8	mir-3160	8
mir-377	7	mir-432	1	mir-501	4	mir-323a	50	mir-376a	50	mir-376b	6	mir-3617	2	mir-4707	2	mir-515	4	mir-3161	1
mir-378a	134	mir-4417	2	mir-502	3	mir-323b	60	mir-376b	25	mir-376c	360	mir-362	155	mir-4726	2	mir-517a	8	mir-3162	4
mir-379	19	mir-4423	2	mir-505	15	mir-324	1282	mir-376c	464	mir-377	2	mir-3622a	4	mir-4727	1	mir-517b	21	mir-3173	2
mir-380	2	mir-4500	17	mir-5100	3	mir-326	611	mir-377	2	mir-378a	882	mir-363	20	mir-4733	1	mir-517c	8	mir-3186	2
mir-381	18	mir-452	15	mir-512	8	mir-328	179	mir-378a	122	mir-379	16	mir-365a	76	mir-4745	2	mir-518c	18	mir-3187	3
mir-382	40	mir-4521	2	mir-515	16	mir-329	14	mir-379	16	mir-380	2	mir-365b	162	mir-4758	10	mir-519a	2	mir-3190	2
mir-3912	1	mir-4524a	1	mir-517	1	mir-330	50	mir-380	2	mir-381	2	mir-3667	3	mir-4802	1	mir-519b	6	mir-3191	1
mir-3928	1	mir-454	54	mir-517a	10	mir-331	1750	mir-381	7	mir-382	15	mir-3679	4	mir-483	5	mir-519d	13	mir-3192	2
mir-3940	2	mir-455	135	mir-517b	9	mir-335	1859	mir-382	27	mir-3912	2	mir-3680	2	mir-484	192	mir-520c	2	mir-3194	2
mir-	4	mir-	2	mir-	6	mir-337	180	mir-	8	mir-	2	mir-369	23	mir-	8	mir-	1	mir-32	60

3944		4639		517c				3928		3925				487b		520d			
mir-3972	1	mir-4640	3	mir-518c	22	mir-338	111	mir-3940	24	mir-3934	2	mir-370	35	mir-491	3	mir-520g	6	mir-320b	14
mir-409	55	mir-4676	8	mir-518d	1	mir-339	4267	mir-3941	2	mir-3960	2	mir-371a	1	mir-493	16	mir-521	2	mir-323a	8
mir-411	10	mir-4689	1	mir-518e	1	mir-33a	435	mir-3944	4	mir-409	68	mir-374a	22	mir-494	3	mir-523	2	mir-323b	5
mir-423	124	mir-4713	1	mir-518f	1	mir-33b	70	mir-3972	2	mir-411	9	mir-374b	88	mir-495	14	mir-525	2	mir-324	73
mir-424	959	mir-4742	2	mir-5195	1	mir-340	549	mir-409	86	mir-423	541	mir-374c	2	mir-497	54	mir-532	48	mir-326	13
mir-425	484	mir-4749	2	mir-519a	3	mir-342	6456	mir-411	6	mir-424	1499	mir-375	3	mir-5008	6	mir-539	2	mir-328	9
mir-4270	1	mir-4753	2	mir-519b	14	mir-345	2866	mir-423	269	mir-425	1286	mir-376a	285	mir-501	8	mir-542	4	mir-331	100
mir-429	96	mir-4777	1	mir-519c	2	mir-34a	2703	mir-424	1254	mir-4260	1	mir-376b	56	mir-502	13	mir-543	2	mir-335	59
mir-4300	1	mir-4781	2	mir-519d	43	mir-34b	430	mir-425	794	mir-429	224	mir-376c	1643	mir-503	6	mir-545	1	mir-337	17
mir-432	6	mir-4787	2	mir-519e	2	mir-34c	837	mir-4270	4	mir-4300	1	mir-377	14	mir-505	43	mir-548q	2	mir-338	40
mir-4330	2	mir-483	4	mir-520a	2	mir-3605	13	mir-429	44	mir-432	2	mir-378a	736	mir-5100	1	mir-551b	8	mir-339	154
mir-4433	2	mir-484	301	mir-520c	4	mir-3607	24	mir-4300	3	mir-433	2	mir-379	97	mir-515	2	mir-5571	1	mir-33a	52
mir-4433b	4	mir-485	3	mir-520e	1	mir-3609	6	mir-4310	1	mir-4423	2	mir-380	4	mir-517a	8	mir-574	84	mir-33b	16
mir-4477a	2	mir-486	4	mir-520f	2	mir-361	749	mir-432	6	mir-4446	1	mir-381	104	mir-517b	22	mir-576	2	mir-340	12
mir-4500	7	mir-487a	3	mir-520g	2	mir-3611	8	mir-433	2	mir-4500	126	mir-382	94	mir-517c	7	mir-582	6	mir-342	200
mir-450a	8	mir-487b	8	mir-521	2	mir-3613	240	mir-4423	1	mir-452	208	mir-3924	2	mir-518c	11	mir-589	2	mir-345	222
mir-452	51	mir-489	2	mir-523	2	mir-3614	3	mir-4433	8	mir-4520b	2	mir-3941	1	mir-518e	5	mir-590	45	mir-34a	1116
mir-4521	5	mir-493	16	mir-524	2	mir-3619	2	mir-4433b	8	mir-4521	13	mir-3944	2	mir-519a	2	mir-598	2	mir-34b	112
mir-4536	2	mir-494	7	mir-525	3	mir-362	230	mir-4480	2	mir-4524a	1	mir-3960	1	mir-519b	1	mir-615	2	mir-34c	121
mir-454	28	mir-495	7	mir-532	42	mir-3620	11	mir-449b	3	mir-4539	2	mir-409	222	mir-519d	10	mir-616	2	mir-3607	53
mir-455	121	mir-497	29	mir-539	2	mir-3622a	2	mir-4500	35	mir-454	170	mir-410	6	mir-520f	1	mir-619	2	mir-3609	25

mir-4646	4	mir-499a	4	mir-542	8	mir-363	24	mir-450a	2	mir-455	243	mir-411	35	mir-520g	4	mir-625	8	mir-361	111
mir-4649	1	mir-5006	1	mir-543	10	mir-3659	2	mir-450b	2	mir-4638	2	mir-423	472	mir-522	8	mir-627	1	mir-3611	1
mir-4652	2	mir-500a	6	mir-545	1	mir-365a	688	mir-452	41	mir-4639	2	mir-424	879	mir-532	124	mir-628	1	mir-3613	46
mir-466	8	mir-501	7	mir-548ba	1	mir-365b	1340	mir-4521	1	mir-4640	1	mir-425	532	mir-542	8	mir-629	3	mir-3614	3
mir-4664	4	mir-502	11	mir-556	2	mir-3663	1	mir-454	74	mir-4652	2	mir-429	19	mir-543	1	mir-6503	1	mir-362	86
mir-4668	8	mir-503	6	mir-5582	1	mir-3664	17	mir-455	226	mir-466	6	mir-4300	1	mir-545	1	mir-651	2	mir-363	20
mir-4685	20	mir-505	61	mir-5591	4	mir-3665	1	mir-4646	2	mir-4662a	2	mir-431	2	mir-548at	10	mir-6514	2	mir-3649	2
mir-4687	7	mir-5089	2	mir-5699	2	mir-3667	6	mir-4652	1	mir-4664	2	mir-432	12	mir-548b	2	mir-652	13	mir-365a	56
mir-4697	2	mir-5100	7	mir-570	4	mir-3677	4	mir-4664	1	mir-4665	1	mir-433	9	mir-548e	4	mir-654	3	mir-365b	88
mir-4700	4	mir-512	2	mir-574	100	mir-3679	2	mir-4684	2	mir-4667	2	mir-4423	3	mir-548t	2	mir-656	2	mir-3664	2
mir-4707	3	mir-517a	4	mir-590	16	mir-3681	18	mir-4687	2	mir-4676	2	mir-4482	2	mir-551b	2	mir-660	54	mir-3667	2
mir-4716	1	mir-517b	8	mir-625	10	mir-3685	4	mir-4690	2	mir-4677	2	mir-449c	2	mir-561	2	mir-664a	6	mir-3675	2
mir-4725	2	mir-518c	2	mir-628	2	mir-369	45	mir-4697	2	mir-4687	2	mir-4500	56	mir-570	4	mir-671	13	mir-369	11
mir-4726	2	mir-519a	4	mir-642a	2	mir-3691	1	mir-4700	2	mir-4697	2	mir-452	34	mir-574	228	mir-6742	1	mir-3692	4
mir-4741	4	mir-519b	2	mir-652	14	mir-370	20	mir-4707	20	mir-4700	1	mir-4524a	4	mir-576	7	mir-676	2	mir-370	4
mir-4742	10	mir-519d	8	mir-654	7	mir-3713	2	mir-4716	3	mir-4709	2	mir-454	32	mir-579	4	mir-6763	1	mir-371a	2
mir-4745	4	mir-520g	10	mir-655	2	mir-371b	2	mir-4722	4	mir-4713	2	mir-455	115	mir-582	12	mir-6769b	2	mir-373	4
mir-4750	9	mir-524	4	mir-659	2	mir-373	5	mir-4731	1	mir-4726	2	mir-4649	8	mir-584	3	mir-6777	2	mir-374a	59
mir-4753	6	mir-532	134	mir-660	9	mir-374a	239	mir-4735	1	mir-4728	2	mir-466	2	mir-589	2	mir-6811	2	mir-374b	95
mir-4777	1	mir-542	11	mir-664a	6	mir-374b	738	mir-4741	4	mir-4742	2	mir-4664	2	mir-590	147	mir-6816	1	mir-376a	16
mir-4783	2	mir-543	8	mir-664b	2	mir-374c	6	mir-4742	4	mir-4749	2	mir-4677	6	mir-615	2	mir-6819	2	mir-376b	18
mir-	2	mir-545	4	mir-671	3	mir-	210	mir-	2	mir-	4	mir-	8	mir-	3	mir-	2	mir-	73

4786						376a		4743		4758		4685		616		6823		376c	
mir-4794	1	mir-548a	4	mir-6715b	2	mir-376b	116	mir-4745	2	mir-4778	4	mir-4700	1	mir-619	4	mir-6824	1	mir-377	6
mir-4799	1	mir-548b	2	mir-6722	2	mir-376c	1534	mir-4750	8	mir-4798	2	mir-4708	1	mir-625	10	mir-6857	1	mir-378a	183
mir-4800	1	mir-548d	2	mir-6734	1	mir-377	8	mir-4753	2	mir-483	18	mir-4726	4	mir-627	2	mir-6858	1	mir-379	2
mir-4801	2	mir-548q	2	mir-675	1	mir-378a	2124	mir-4758	1	mir-484	822	mir-4731	2	mir-628	2	mir-6865	2	mir-381	3
mir-4802	2	mir-550a	4	mir-6786	2	mir-378f	4	mir-4764	1	mir-485	2	mir-4742	2	mir-629	2	mir-6881	2	mir-382	3
mir-483	15	mir-551a	20	mir-6791	3	mir-379	48	mir-4778	2	mir-486	4	mir-4745	1	mir-642a	1	mir-6887	2	mir-383	1
mir-484	165	mir-551b	4	mir-6814	1	mir-380	4	mir-4783	2	mir-487a	2	mir-4747	2	mir-6503	5	mir-7	18	mir-3917	2
mir-486	24	mir-574	543	mir-6844	4	mir-381	46	mir-4801	2	mir-487b	25	mir-4750	1	mir-6511b	4	mir-708	76	mir-3922	1
mir-487a	1	mir-576	5	mir-6853	2	mir-382	39	mir-483	9	mir-489	18	mir-4783	2	mir-652	35	mir-7109	2	mir-3925	2
mir-487b	38	mir-582	2	mir-6854	1	mir-383	105	mir-484	830	mir-491	1	mir-4795	1	mir-654	4	mir-744	4	mir-3928	1
mir-491	4	mir-585	3	mir-6870	2	mir-3912	11	mir-485	12	mir-493	14	mir-4802	1	mir-660	82	mir-767	2	mir-3934	2
mir-493	20	mir-590	43	mir-6875	1	mir-3922	1	mir-486	31	mir-494	12	mir-483	23	mir-664a	27	mir-769	2	mir-3940	2
mir-494	13	mir-598	4	mir-6878	2	mir-3928	6	mir-487a	1	mir-495	17	mir-484	639	mir-664b	2	mir-874	2	mir-3944	8
mir-495	23	mir-615	28	mir-6891	1	mir-3934	4	mir-487b	45	mir-497	72	mir-485	39	mir-671	22	mir-877	1	mir-3960	33
mir-497	105	mir-619	2	mir-7	5	mir-3940	39	mir-489	4	mir-499a	2	mir-486	26	mir-6749	2	mir-885	1	mir-3972	4
mir-499a	1	mir-624	2	mir-708	24	mir-3960	11	mir-493	42	mir-499b	1	mir-487a	18	mir-6752	2	mir-887	1	mir-409	13
mir-5008	16	mir-625	51	mir-7110	1	mir-3972	5	mir-494	31	mir-5000	2	mir-487b	178	mir-6756	2	mir-92a	102	mir-411	5
mir-500a	11	mir-627	1	mir-7157	1	mir-409	181	mir-495	44	mir-5008	2	mir-489	10	mir-6757	1	mir-92b	2	mir-423	107
mir-500b	1	mir-628	5	mir-7162	1	mir-410	3	mir-497	41	mir-500a	11	mir-491	3	mir-6767	2	mir-93	71	mir-424	1907
mir-501	7	mir-629	13	mir-744	7	mir-411	13	mir-499a	8	mir-500b	2	mir-493	77	mir-6777	1	mir-95	8	mir-425	735
mir-502	41	mir-6502	3	mir-766	6	mir-412	2	mir-5002	8	mir-501	21	mir-494	103	mir-6781	2	mir-98	30	mir-4251	2

mir-503	3	mir-6503	1	mir-769	4	mir-422a	2	mir-5003	1	mir-502	31	mir-495	58	mir-6786	1	mir-99a	327	mir-429	94
mir-504	4	mir-651	2	mir-7854	2	mir-423	602	mir-5004	1	mir-503	28	mir-497	268	mir-6802	1	mir-99b	145	mir-4293	1
mir-505	48	mir-652	68	mir-874	4	mir-424	4127	mir-5006	1	mir-504	2	mir-499a	24	mir-6820	2			mir-4300	3
mir-506	2	mir-654	9	mir-885	1	mir-425	7221	mir-5008	2	mir-505	93	mir-5001	2	mir-6822	1			mir-4320	4
mir-5100	8	mir-656	2	mir-887	1	mir-4251	2	mir-500a	2	mir-5089	1	mir-5008	6	mir-6849	2			mir-433	8
mir-512	8	mir-659	1	mir-889	2	mir-4270	16	mir-501	11	mir-510	2	mir-500a	29	mir-6868	2			mir-4417	742
mir-513b	2	mir-660	44	mir-92a	43	mir-429	1393	mir-502	15	mir-5100	5	mir-500b	1	mir-6873	2			mir-4425	5
mir-515	4	mir-664a	111	mir-92b	13	mir-4300	10	mir-503	23	mir-511	2	mir-501	46	mir-7	59			mir-4430	20
mir-517a	6	mir-664b	4	mir-93	173	mir-431	24	mir-504	2	mir-512	2	mir-5010	4	mir-708	410			mir-4433	2
mir-517b	21	mir-665	1	mir-95	12	mir-4310	1	mir-505	86	mir-514b	2	mir-502	53	mir-744	24			mir-4433b	4
mir-5189	2	mir-671	36	mir-96	4	mir-4320	1	mir-508	1	mir-515	4	mir-503	24	mir-767	13			mir-4472	8
mir-518c	12	mir-6742	1	mir-98	28	mir-433	12	mir-5089	2	mir-517a	12	mir-505	60	mir-769	5			mir-4500	43
mir-518d	2	mir-675	4	mir-99a	670	mir-4330	1	mir-5100	13	mir-517b	34	mir-5100	26	mir-874	6			mir-450b	4
mir-518e	3	mir-6751	2	mir-99b	203	mir-4417	2	mir-512	8	mir-517c	2	mir-511	4	mir-877	7			mir-452	305
mir-518f	1	mir-6752	2			mir-4423	14	mir-515	6	mir-5187	2	mir-512	8	mir-887	8			mir-4520b	1
mir-519a	4	mir-676	1			mir-4430	6	mir-517a	12	mir-518c	8	mir-517a	12	mir-889	4			mir-4521	10
mir-519b	6	mir-6762	2			mir-4433	2	mir-517b	25	mir-519a	5	mir-517b	8	mir-92a	149			mir-4524a	1
mir-519d	22	mir-6767	2			mir-4480	4	mir-517c	10	mir-519b	6	mir-517c	1	mir-92b	11			mir-4529	13
mir-519e	2	mir-6777	2			mir-449b	2	mir-518c	10	mir-519c	2	mir-518c	8	mir-93	851			mir-4539	1
mir-520a	3	mir-6781	2			mir-4500	642	mir-519a	2	mir-519d	17	mir-518d	1	mir-938	1			mir-454	78
mir-520d	1	mir-6784	1			mir-450a	6	mir-519b	14	mir-519e	4	mir-519a	4	mir-940	6			mir-455	123
mir-	4	mir-	2			mir-	16	mir-	2	mir-	2	mir-	30	mir-	18			mir-	2

520g		6789	
mir-521	2	mir-6791	2
mir-525	6	mir-6795	2
mir-532	174	mir-6796	2
mir-539	6	mir-6797	2
mir-542	20	mir-6803	2
mir-543	6	mir-6807	1
mir-545	3	mir-6810	2
mir-548ac	2	mir-6816	2
mir-548ao	2	mir-6822	7
mir-548ap	2	mir-6830	1
mir-548aq	2	mir-6837	1
mir-548ar	2	mir-6850	1
mir-548b	4	mir-6855	2
mir-548d	4	mir-6869	1
mir-548t	5	mir-6873	2
mir-550a	2	mir-6882	2
mir-550b	2	mir-6887	2
mir-551a	2	mir-7	61
mir-551b	36	mir-708	127
mir-5579	2	mir-7108	2
mir-5584	2	mir-7158	1

450b		519c		520c		519d		95	
mir-452	340	mir-519d	9	mir-520e	1	mir-520g	10	mir-96	80
mir-4520b	8	mir-519e	3	mir-520f	1	mir-521	2	mir-98	118
mir-4521	17	mir-520a	2	mir-520g	18	mir-524	2	mir-99a	3249
mir-4524a	1	mir-520c	4	mir-521	2	mir-532	580	mir-99b	106
mir-454	1083	mir-520f	2	mir-524	2	mir-539	18		
mir-455	1355	mir-520g	24	mir-525	3	mir-542	28		
mir-4632	2	mir-521	2	mir-526b	6	mir-543	17		
mir-4635	1	mir-525	4	mir-532	496	mir-545	18		
mir-4638	1	mir-526b	2	mir-539	3	mir-548as	2		
mir-4640	3	mir-532	391	mir-542	21	mir-548b	5		
mir-4641	2	mir-539	2	mir-543	1	mir-548e	4		
mir-4646	8	mir-542	20	mir-545	9	mir-548f	2		
mir-4652	6	mir-543	19	mir-548a	4	mir-548h	4		
mir-4654	1	mir-544a	4	mir-548ah	1	mir-548q	4		
mir-4662a	2	mir-545	17	mir-548aq	2	mir-551a	4		
mir-4664	2	mir-548e	1	mir-548d	3	mir-551b	12		
mir-4665	2	mir-550a	4	mir-548e	1	mir-5571	1		
mir-4666b	4	mir-551b	34	mir-548f	4	mir-5579	2		
mir-4667	6	mir-553	1	mir-548q	1	mir-561	1		
mir-4677	8	mir-556	1	mir-548x	1	mir-574	1037		
mir-4690	28	mir-5584	2	mir-550a	6	mir-576	4		

4632	
mir-4635	1
mir-4645	4
mir-4652	2
mir-4655	1
mir-4659a	1
mir-4661	1
mir-4665	2
mir-4666a	1
mir-4667	3
mir-4668	10
mir-4670	2
mir-4676	4
mir-4677	2
mir-4680	8
mir-4685	1
mir-4687	2
mir-4689	2
mir-4700	10
mir-4701	1
mir-4705	1
mir-4707	1

mir-5585	24	mir-744	131
mir-5586	1	mir-761	2
mir-5588	1	mir-766	12
mir-561	4	mir-769	32
mir-5681a	1	mir-7845	2
mir-570	2	mir-7852	2
mir-574	680	mir-874	46
mir-576	13	mir-877	8
mir-579	4	mir-885	2
mir-582	49	mir-887	8
mir-584	2	mir-92a	364
mir-585	4	mir-92b	102
mir-589	6	mir-93	2353
mir-590	112	mir-939	1
mir-598	10	mir-940	17
mir-6068	1	mir-942	2
mir-6079	1	mir-95	10
mir-6084	2	mir-96	72
mir-615	2	mir-98	53
mir-616	2	mir-99a	2824
mir-619	2	mir-99b	355
mir-	49		

mir-4700	1	mir-5585	6	mir-551a	4	mir-579	2
mir-4707	52	mir-574	864	mir-551b	71	mir-582	6
mir-4708	1	mir-576	9	mir-561	1	mir-584	2
mir-4713	3	mir-579	8	mir-570	4	mir-585	2
mir-4714	5	mir-582	16	mir-574	512	mir-589	4
mir-4715	2	mir-584	2	mir-576	13	mir-590	207
mir-4723	6	mir-585	2	mir-579	10	mir-598	1
mir-4725	1	mir-589	8	mir-582	34	mir-610	2
mir-4726	2	mir-590	299	mir-584	4	mir-615	2
mir-4728	18	mir-605	2	mir-589	14	mir-616	4
mir-4731	2	mir-6079	1	mir-590	654	mir-619	18
mir-4741	1	mir-6127	1	mir-598	12	mir-624	8
mir-4742	4	mir-615	24	mir-6079	1	mir-625	51
mir-4745	2	mir-616	4	mir-615	5	mir-627	3
mir-4746	3	mir-619	2	mir-616	5	mir-628	6
mir-4747	2	mir-624	4	mir-619	3	mir-629	3
mir-4750	2	mir-625	83	mir-624	4	mir-642a	7
mir-4753	1	mir-627	2	mir-625	128	mir-6499	2
mir-4757	1	mir-628	10	mir-627	18	mir-6501	1
mir-4760	4	mir-629	4	mir-628	5	mir-6503	22
mir-4761	2	mir-642a	8	mir-629	10	mir-6504	1
mir-	1	mir-	2	mir-	5	mir-	2

mir-4708	16
mir-4709	1
mir-4713	4
mir-4714	2
mir-4716	1
mir-4722	2
mir-4725	2
mir-4726	1
mir-4735	1
mir-4742	2
mir-4745	3
mir-4746	2
mir-4747	2
mir-4750	2
mir-4753	2
mir-4756	1
mir-4758	2
mir-4762	2
mir-4763	1
mir-4768	1
mir-4777	2
mir-	6

625	
mir-627	9
mir-628	18
mir-629	5
mir-642b	5
mir-6499	2
mir-6500	1
mir-6502	2
mir-6503	3
mir-6506	2
mir-6511b	2
mir-6516	3
mir-652	23
mir-654	15
mir-655	6
mir-656	16
mir-659	1
mir-660	105
mir-664a	48
mir-664b	6
mir-668	1
mir-671	37

4762		6503		642a		6505	
mir-4772	7	mir-651	2	mir-6502	1	mir-6512	2
mir-4777	1	mir-6513	2	mir-6503	3	mir-6514	2
mir-4778	33	mir-652	77	mir-6510	2	mir-652	172
mir-4781	1	mir-654	36	mir-6514	2	mir-654	77
mir-4783	49	mir-655	12	mir-652	186	mir-655	18
mir-4787	2	mir-656	8	mir-654	9	mir-656	15
mir-4794	2	mir-659	3	mir-655	6	mir-660	237
mir-4798	2	mir-660	63	mir-656	4	mir-664a	36
mir-4802	2	mir-664a	18	mir-659	3	mir-664b	8
mir-4804	1	mir-664b	8	mir-660	111	mir-665	9
mir-483	22	mir-665	2	mir-664a	69	mir-668	1
mir-484	4877	mir-671	75	mir-664b	8	mir-671	76
mir-485	14	mir-6715a	1	mir-665	1	mir-6716	4
mir-486	91	mir-6715b	1	mir-671	141	mir-6720	2
mir-487a	22	mir-6716	9	mir-6716	1	mir-6721	2
mir-487b	208	mir-6721	2	mir-6720	6	mir-6726	2
mir-489	41	mir-6722	6	mir-6723	1	mir-6727	1
mir-491	10	mir-6729	6	mir-6726	3	mir-6731	2
mir-493	45	mir-6732	4	mir-6734	1	mir-6734	2
mir-494	46	mir-6743	3	mir-6740	2	mir-6737	2
mir-495	54	mir-6746	2	mir-6747	2	mir-6745	1

4778	
mir-4783	1
mir-4786	8
mir-4787	2
mir-4789	1
mir-4793	2
mir-4796	6
mir-4800	1
mir-4804	1
mir-483	25
mir-484	239
mir-485	5
mir-486	4
mir-487a	6
mir-487b	42
mir-489	4
mir-490	1
mir-491	4
mir-492	9
mir-494	2
mir-495	12
mir-497	61

mir-6716	1
mir-6721	2
mir-6722	2
mir-6723	1
mir-6731	2
mir-6734	2
mir-6743	7
mir-6749	47
mir-675	7
mir-6750	2
mir-6753	1
mir-6755	2
mir-676	2
mir-6766	2
mir-6771	2
mir-6779	2
mir-6780b	2
mir-6783	1
mir-6786	1
mir-6791	1
mir-6793	2
mir-	2

mir-497	106	mir-6749	22	mir-675	5	mir-675	21
mir-499a	5	mir-675	3	mir-6752	2	mir-6751	1
mir-5000	1	mir-676	1	mir-6756	2	mir-6752	4
mir-5001	2	mir-6766	2	mir-6759	1	mir-6755	2
mir-5006	2	mir-6768	3	mir-6767	2	mir-6756	2
mir-5008	3	mir-6779	3	mir-6769b	2	mir-6762	1
mir-500a	82	mir-6781	4	mir-6773	1	mir-6769b	4
mir-500b	2	mir-6782	3	mir-6777	2	mir-6779	1
mir-501	18	mir-6784	1	mir-6779	2	mir-6782	3
mir-5011	2	mir-6791	2	mir-6791	5	mir-6791	2
mir-502	92	mir-6793	4	mir-6796	1	mir-6793	2
mir-503	106	mir-6795	1	mir-6797	2	mir-6796	8
mir-504	1	mir-6802	2	mir-6803	6	mir-6803	4
mir-505	1119	mir-6803	10	mir-6807	2	mir-6810	2
mir-5095	1	mir-6804	1	mir-6813	2	mir-6814	2
mir-5100	79	mir-6805	1	mir-6822	1	mir-6815	1
mir-512	14	mir-6806	2	mir-6826	2	mir-6822	2
mir-513c	2	mir-6810	2	mir-6830	6	mir-6830	9
mir-514b	2	mir-6812	4	mir-6835	1	mir-6838	2
mir-515	10	mir-6820	4	mir-6838	1	mir-6850	1
mir-517a	16	mir-6821	1	mir-6845	6	mir-6855	2
mir-	36	mir-	8	mir-	1	mir-	3

mir-499a	4
mir-5001	16
mir-5008	7
mir-5009	1
mir-500a	7
mir-501	1
mir-5010	3
mir-502	20
mir-503	8
mir-504	8
mir-5047	2
mir-505	82
mir-508	1
mir-5088	8
mir-5095	9
mir-510	2
mir-5100	6
mir-512	26
mir-514b	7
mir-515	36
mir-517a	74
mir-	199

6798	
mir-6800	1
mir-6802	1
mir-6812	10
mir-6815	4
mir-6824	1
mir-6825	1
mir-6841	1
mir-6844	1
mir-6855	6
mir-6857	2
mir-6865	3
mir-6877	2
mir-6880	2
mir-6887	4
mir-6895	1
mir-7	73
mir-708	681
mir-7110	3
mir-7157	2
mir-744	33
mir-766	18

517b		6833		6850		6858	
mir-517c	8	mir-6848	1	mir-6851	2	mir-6870	2
mir-5187	8	mir-6849	2	mir-6854	8	mir-6872	3
mir-518c	26	mir-6851	2	mir-6855	2	mir-6875	2
mir-518e	4	mir-6852	1	mir-6856	1	mir-6878	1
mir-519a	8	mir-6854	6	mir-6858	4	mir-7	58
mir-519b	8	mir-6879	2	mir-6861	8	mir-708	479
mir-519c	2	mir-6887	10	mir-6870	2	mir-7109	4
mir-519d	84	mir-6889	2	mir-6879	4	mir-7111	2
mir-519e	8	mir-7	118	mir-6885	2	mir-744	64
mir-520a	2	mir-708	590	mir-6886	1	mir-758	2
mir-520c	10	mir-7106	4	mir-6887	10	mir-761	2
mir-520e	2	mir-7107	2	mir-6890	1	mir-766	43
mir-520f	5	mir-7111	2	mir-7	140	mir-767	2
mir-520g	6	mir-7152	1	mir-708	1310	mir-769	32
mir-521	14	mir-744	36	mir-7106	2	mir-874	151
mir-524	2	mir-758	4	mir-7108	4	mir-877	7
mir-525	12	mir-766	23	mir-7111	8	mir-887	51
mir-532	6209	mir-769	22	mir-744	156	mir-9	2
mir-539	2	mir-7845	3	mir-766	22	mir-92a	670
mir-542	97	mir-7854	4	mir-767	41	mir-92b	91
mir-543	29	mir-873	8	mir-769	34	mir-93	1053

517b	
mir-517c	41
mir-5189	2
mir-518a	2
mir-518c	51
mir-518d	1
mir-518e	19
mir-5195	1
mir-5196	2
mir-519a	4
mir-519b	4
mir-519d	103
mir-519e	11
mir-520a	3
mir-520c	1
mir-520d	4
mir-520f	4
mir-520g	18
mir-521	18
mir-522	13
mir-523	8
mir-524	13

mir-767	4
mir-769	6
mir-7847	2
mir-874	45
mir-877	8
mir-885	2
mir-887	3
mir-889	4
mir-92a	282
mir-92b	41
mir-93	1234
mir-938	1
mir-940	8
mir-95	18
mir-96	62
mir-98	143
mir-99a	2758
mir-99b	181

mir-545	74	mir-874	45	mir-874	25	mir-940	39
mir-548a	14	mir-876	6	mir-877	9	mir-95	16
mir-548ah	3	mir-877	18	mir-887	17	mir-96	16
mir-548ao	8	mir-887	10	mir-9	2	mir-98	72
mir-548ap	2	mir-889	6	mir-92a	4207	mir-99a	8079
mir-548aq	6	mir-92a	1016	mir-92b	212	mir-99b	460
mir-548ar	2	mir-92b	97	mir-93	4597		
mir-548at	1	mir-93	2737	mir-940	54		
mir-548ay	1	mir-940	20	mir-942	1		
mir-548b	4	mir-95	72	mir-95	67		
mir-548ba	2	mir-96	64	mir-96	196		
mir-548d	8	mir-98	243	mir-98	424		
mir-548e	4	mir-99a	1443	mir-99a	4487		
mir-548h	6	mir-99b	278	mir-99b	268		
mir-548o	14						
mir-548q	7						
mir-548t	26						
mir-548x	3						
mir-550a	60						
mir-551a	217						
mir-551b	129						
mir-552	2						

mir-525	16
mir-532	337
mir-539	6
mir-542	44
mir-543	7
mir-544a	2
mir-545	27
mir-548aq	4
mir-548at	2
mir-548b	4
mir-548d	3
mir-548e	2
mir-548f	2
mir-548o	2
mir-548q	6
mir-548t	4
mir-550a	10
mir-551a	10
mir-551b	13
mir-553	1
mir-556	3
mir-	2

mir-556	2
mir-5571	1
mir-5579	2
mir-5581	4
mir-5582	2
mir-5583	2
mir-5585	8
mir-5586	3
mir-5588	6
mir-5591	8
mir-561	11
mir-5699	30
mir-570	4
mir-574	3191
mir-576	48
mir-5787	2
mir-579	18
mir-581	1
mir-582	27
mir-584	18
mir-585	23

5579	
mir-5582	3
mir-5585	24
mir-5590	2
mir-561	9
mir-5684	2
mir-5697	2
mir-5699	4
mir-570	6
mir-571	1
mir-574	401
mir-576	6
mir-5787	4
mir-579	6
mir-582	33
mir-584	2
mir-589	2
mir-590	315
mir-591	1
mir-593	1
mir-598	6
mir-6079	1

mir-589	20
mir-590	3553
mir-597	8
mir-598	389
mir-603	3
mir-610	1
mir-615	272
mir-616	26
mir-619	22
mir-624	14
mir-625	1396
mir-627	45
mir-628	11
mir-629	65
mir-642a	11
mir-6500	16
mir-6501	1
mir-6503	28
mir-6505	3
mir-6508	2
mir-6509	4
mir-651	7

mir-615	4
mir-616	4
mir-619	102
mir-624	4
mir-625	41
mir-627	21
mir-628	4
mir-629	11
mir-642a	1
mir-642b	2
mir-6499	1
mir-6500	1
mir-6502	1
mir-6503	2
mir-6504	2
mir-6505	4
mir-6506	2
mir-6507	1
mir-651	4
mir-6510	9
mir-6511a	2
mir-	3

mir-6511b	8
mir-6512	1
mir-6514	10
mir-6515	14
mir-6516	1
mir-652	4759
mir-654	68
mir-655	20
mir-656	4
mir-660	398
mir-663b	2
mir-664a	253
mir-664b	40
mir-665	17
mir-668	3
mir-670	1
mir-671	545
mir-6723	10
mir-6726	2
mir-6730	1
mir-6731	6

6512	
mir-6516	8
mir-652	79
mir-654	1
mir-655	5
mir-656	2
mir-657	2
mir-659	1
mir-660	127
mir-663b	8
mir-664a	26
mir-664b	7
mir-665	3
mir-668	2
mir-671	59
mir-6722	2
mir-6727	1
mir-6728	2
mir-6732	4
mir-6733	1
mir-6734	4
mir-6735	4

mir-6732	1
mir-6734	8
mir-6735	2
mir-6737	2
mir-6742	4
mir-6743	2
mir-6745	1
mir-6747	4
mir-6749	4
mir-675	4
mir-6750	2
mir-6752	2
mir-6754	3
mir-6756	6
mir-6757	2
mir-6758	2
mir-6761	2
mir-6763	9
mir-6764	4
mir-6766	1
mir-6767	3
mir-	1

mir-6739	2
mir-6740	1
mir-6741	8
mir-6742	2
mir-6743	2
mir-6746	2
mir-6747	1
mir-6748	2
mir-6749	10
mir-675	4
mir-6750	2
mir-6751	10
mir-6752	2
mir-6754	2
mir-6755	2
mir-6756	4
mir-6757	2
mir-6759	4
mir-6762	1
mir-6765	4
mir-6766	1
mir-	2

6768	
mir-6769b	14
mir-6772	1
mir-6773	1
mir-6774	4
mir-6777	4
mir-6779	2
mir-6780b	2
mir-6783	3
mir-6789	8
mir-6791	213
mir-6793	5
mir-6795	2
mir-6796	4
mir-6797	19
mir-6798	1
mir-6799	2
mir-6801	5
mir-6802	2
mir-6803	18
mir-6804	2
mir-6805	2

6767	
mir-6769a	4
mir-6769b	6
mir-6771	3
mir-6772	2
mir-6773	1
mir-6774	6
mir-6775	4
mir-6777	1
mir-6779	4
mir-6780a	3
mir-6780b	3
mir-6781	2
mir-6783	6
mir-6784	1
mir-6786	2
mir-6788	2
mir-6789	2
mir-6790	4
mir-6791	41
mir-6793	4
mir-6794	1

mir-6806	2
mir-6809	2
mir-6810	8
mir-6812	6
mir-6813	2
mir-6815	4
mir-6816	1
mir-6818	2
mir-6819	2
mir-6820	2
mir-6822	2
mir-6824	4
mir-6825	6
mir-6827	2
mir-6829	1
mir-6830	4
mir-6831	2
mir-6835	1
mir-6836	4
mir-6837	2
mir-6839	6
mir-	3

mir-6795	16
mir-6796	2
mir-6797	3
mir-6798	2
mir-6801	2
mir-6803	3
mir-6806	1
mir-6807	2
mir-6808	1
mir-6809	12
mir-6810	2
mir-6811	2
mir-6812	7
mir-6816	2
mir-6818	2
mir-6821	4
mir-6822	3
mir-6825	12
mir-6830	5
mir-6831	4
mir-6832	2
mir-	4

6846	
mir-6847	3
mir-6848	1
mir-6850	7
mir-6851	3
mir-6854	4
mir-6855	4
mir-6856	2
mir-6858	8
mir-6866	4
mir-6867	2
mir-6869	1
mir-6870	2
mir-6873	2
mir-6879	2
mir-6880	14
mir-6882	1
mir-6883	4
mir-6884	1
mir-6886	8
mir-6887	38
mir-6890	2

6833	
mir-6834	1
mir-6835	1
mir-6836	4
mir-6838	7
mir-6839	2
mir-6840	3
mir-6842	2
mir-6846	5
mir-6848	1
mir-6849	4
mir-6850	8
mir-6851	3
mir-6852	2
mir-6857	1
mir-6858	3
mir-6862	2
mir-6864	1
mir-6866	8
mir-6867	2
mir-6869	2
mir-6870	2

mir-6895	2
mir-7	1343
mir-708	3843
mir-7106	2
mir-7108	6
mir-7110	4
mir-7111	10
mir-7112	2
mir-7152	3
mir-744	315
mir-758	8
mir-759	2
mir-766	171
mir-767	4
mir-769	406
mir-7843	4
mir-7844	1
mir-7847	2
mir-7851	2
mir-7854	1
mir-7855	2
mir-802	5

mir-6873	8
mir-6875	1
mir-6876	2
mir-6878	1
mir-6879	3
mir-6880	2
mir-6882	1
mir-6884	4
mir-6886	2
mir-6887	4
mir-6889	1
mir-6891	1
mir-6893	4
mir-6894	4
mir-7	59
mir-708	488
mir-7106	8
mir-7107	10
mir-7108	2
mir-7109	3
mir-711	2
mir-7110	1

mir-873	7
mir-874	103
mir-876	2
mir-877	58
mir-885	48
mir-887	32
mir-889	8
mir-9	136
mir-92a	2100
mir-92b	800
mir-93	12477
mir-939	6
mir-940	294
mir-942	15
mir-95	584
mir-96	2219
mir-98	1847
mir-99a	19652 2
mir-99b	1604

mir-7111	4
mir-7113	5
mir-7114	1
mir-7156	2
mir-7160	2
mir-744	30
mir-766	2
mir-769	20
mir-770	2
mir-7843	3
mir-7846	1
mir-7847	3
mir-7848	1
mir-7850	1
mir-7851	5
mir-873	3
mir-874	23
mir-876	4
mir-877	10
mir-885	1
mir-887	3
mir-	1

888	
mir-889	2
mir-891a	2
mir-9	2
mir-921	4
mir-92a	239
mir-92b	19
mir-93	1028
mir-939	3
mir-940	17
mir-942	1
mir-95	14
mir-96	219
mir-98	612
mir-99a	2621
mir-99b	196
