# Supplementary material for: Small RNAs in metastatic and non-metastatic oral squamous cell carcinoma
Source: BMC Med Genomics. 2015 Jun 24;8:31. doi: 10.1186/s12920-015-0102-4 (PMC4479233; doi:10.1186/s12920-015-0102-4)
Supplement: Additional file 4: — Most expressed miRNAs in non-metastatic tumor samples. The graph shows the most expressed miRNAs per sample (x-axis) and correspondent read counts (y-axis). Numbers on top of each bar correspond to the cumulative percentage considering the total number of read counts per sample. [file 12920_2015_102_MOESM4_ESM.pdf]

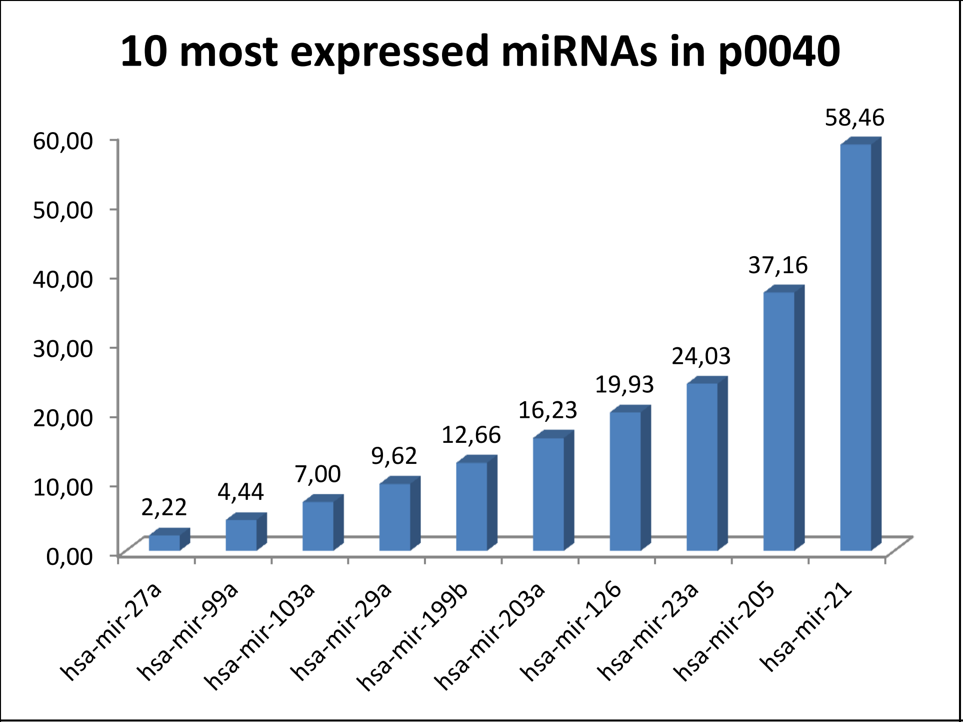
10 most expressed miRNAs in p0040
60,00
50,00
40,00
30,00
20,00
10,00
0,00
2,22
4,44
7,00
9,62
12,66
16,23
19,93
24,03
37,16
58,46
hsa-mir-27a
hsa-mir-99a
hsa-mir-103a
hsa-mir-29a
hsa-mir-199b
hsa-mir-203a
hsa-mir-126
hsa-mir-23a
hsa-mir-205
hsa-mir-21

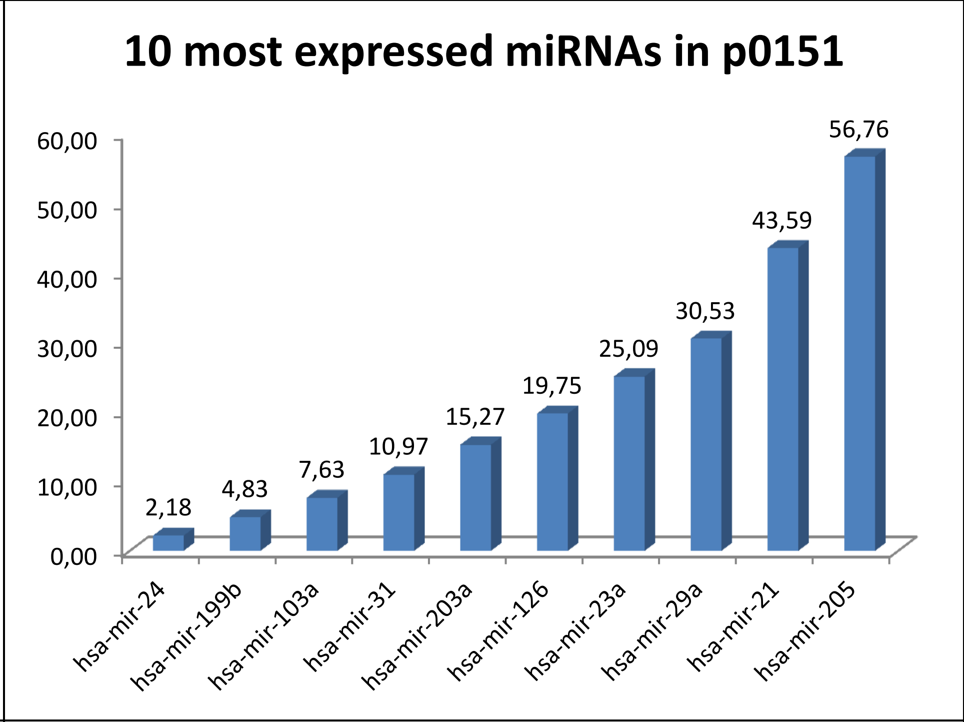
10 most expressed miRNAs in p0151
60,00
50,00
40,00
30,00
20,00
10,00
0,00
2,18
4,83
7,63
10,97
15,27
19,75
25,09
30,53
43,59
56,76
hsa-mir-24
hsa-mir-199b
hsa-mir-103a
hsa-mir-31
hsa-mir-203a
hsa-mir-126
hsa-mir-23a
hsa-mir-29a
hsa-mir-21
hsa-mir-205

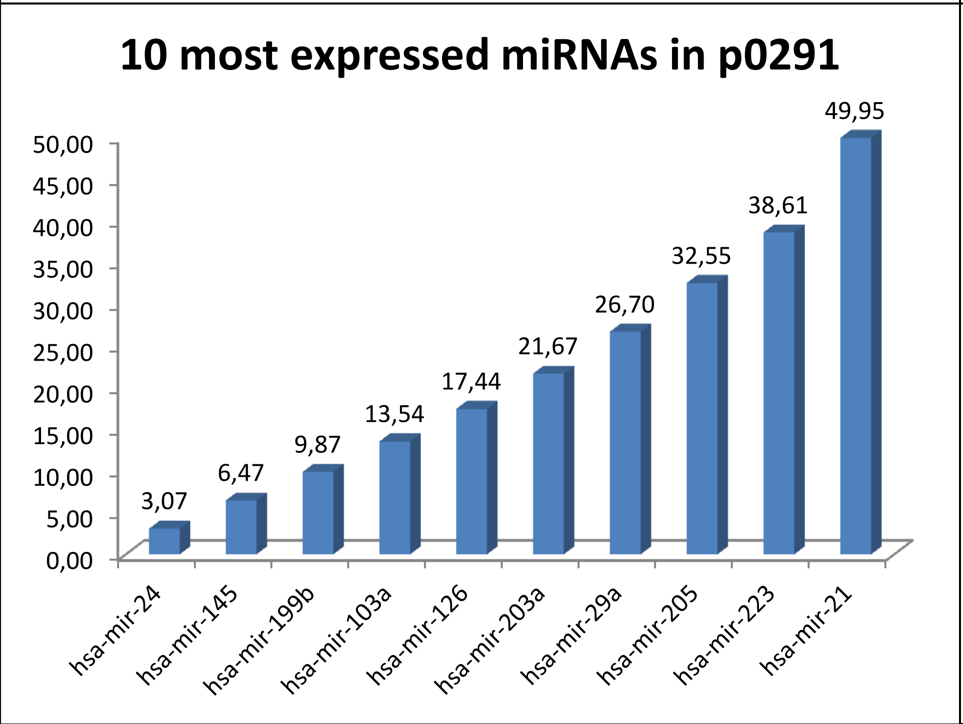
10 most expressed miRNAs in p0291
50,00
45,00
40,00
35,00
30,00
25,00
20,00
15,00
10,00
5,00
0,00
3,07
6,47
9,87
13,54
17,44
21,67
26,70
32,55
38,61
49,95
hsa-mir-24
hsa-mir-145
hsa-mir-199b
hsa-mir-103a
hsa-mir-126
hsa-mir-203a
hsa-mir-29a
hsa-mir-205
hsa-mir-223
hsa-mir-21

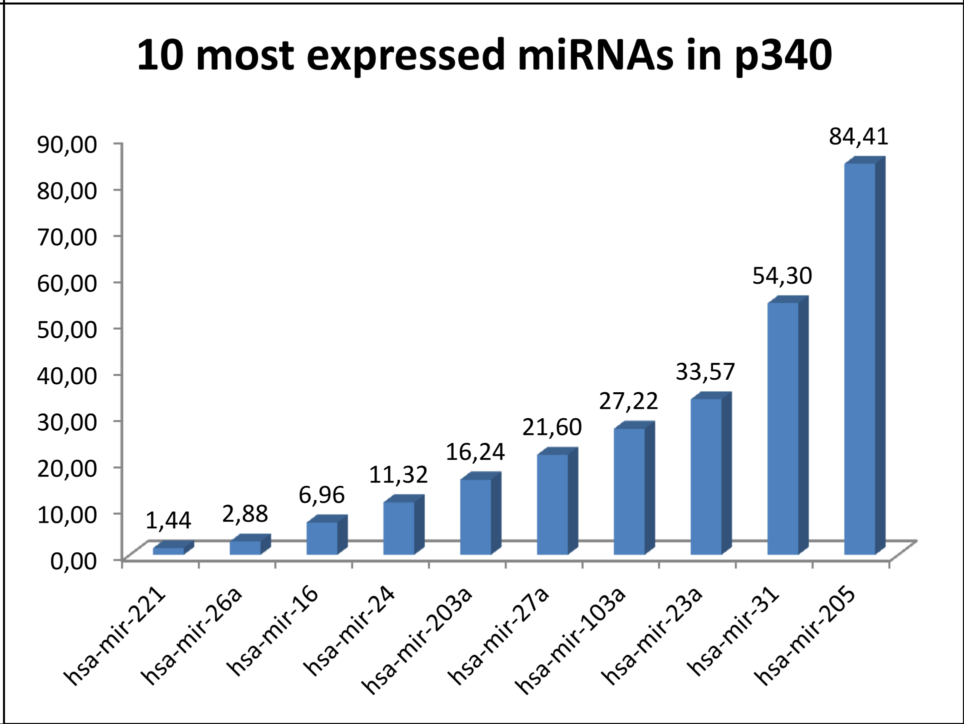
10 most expressed miRNAs in p340
90,00
80,00
70,00
60,00
50,00
40,00
30,00
20,00
10,00
0,00
1,44
2,88
6,96
11,32
16,24
21,60
27,22
33,57
54,30
84,41
hsa-mir-221
hsa-mir-26a
hsa-mir-16
hsa-mir-24
hsa-mir-203a
hsa-mir-27a
hsa-mir-103a
hsa-mir-23a
hsa-mir-31
hsa-mir-205

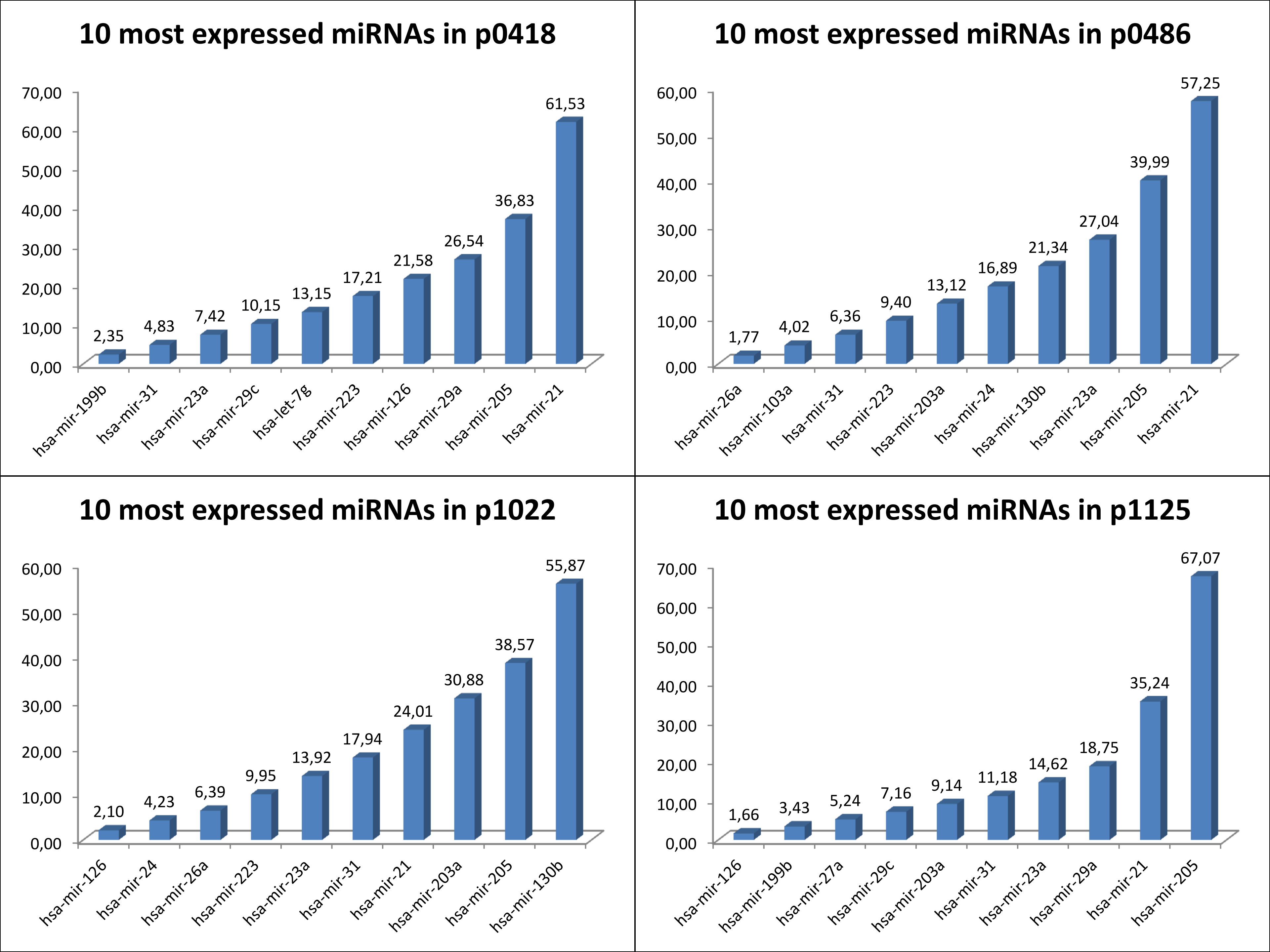
10 most expressed miRNAs in p0418
70,00
60,00
50,00
40,00
30,00
20,00
10,00
0,00
2,35
4,83
7,42
10,15
13,15
17,21
21,58
26,54
36,83
61,53
hsa-mir-199b
hsa-mir-31
hsa-mir-23a
hsa-mir-29c
hsa-let-7g
hsa-mir-223
hsa-mir-126
hsa-mir-29a
hsa-mir-205
hsa-mir-21
10 most expressed miRNAs in p0486
60,00
50,00
40,00
30,00
20,00
10,00
0,00
1,77
4,02
6,36
9,40
13,12
16,89
21,34
27,04
39,99
57,25
hsa-mir-26a
hsa-mir-103a
hsa-mir-31
hsa-mir-223
hsa-mir-203a
hsa-mir-24
hsa-mir-130b
hsa-mir-23a
hsa-mir-205
hsa-mir-21
10 most expressed miRNAs in p1022
60,00
50,00
40,00
30,00
20,00
10,00
0,00
2,10
4,23
6,39
9,95
13,92
17,94
24,01
30,88
38,57
55,87
hsa-mir-126
hsa-mir-24
hsa-mir-26a
hsa-mir-223
hsa-mir-23a
hsa-mir-31
hsa-mir-21
hsa-mir-203a
hsa-mir-205
hsa-mir-130b
10 most expressed miRNAs in p1125
70,00
60,00
50,00
40,00
30,00
20,00
10,00
0,00
1,66
3,43
5,24
7,16
9,14
11,18
14,62
18,75
35,24
67,07
hsa-mir-126
hsa-mir-199b
hsa-mir-27a
hsa-mir-29c
hsa-mir-203a
hsa-mir-31
hsa-mir-23a
hsa-mir-29a
hsa-mir-21
hsa-mir-205
