# Supplementary material for: Small RNAs in metastatic and non-metastatic oral squamous cell carcinoma
Source: BMC Med Genomics. 2015 Jun 24;8:31. doi: 10.1186/s12920-015-0102-4 (PMC4479233; doi:10.1186/s12920-015-0102-4)
Supplement: Additional file 5: — Most expressed miRNAs in metastatic tumor samples. The graph shows the most expressed miRNAs per sample (x-axis) and correspondent read counts (y-axis). Numbers on top of each bar correspond to the cumulative percentage considering the total number of read counts per sample. [file 12920_2015_102_MOESM5_ESM.pdf]

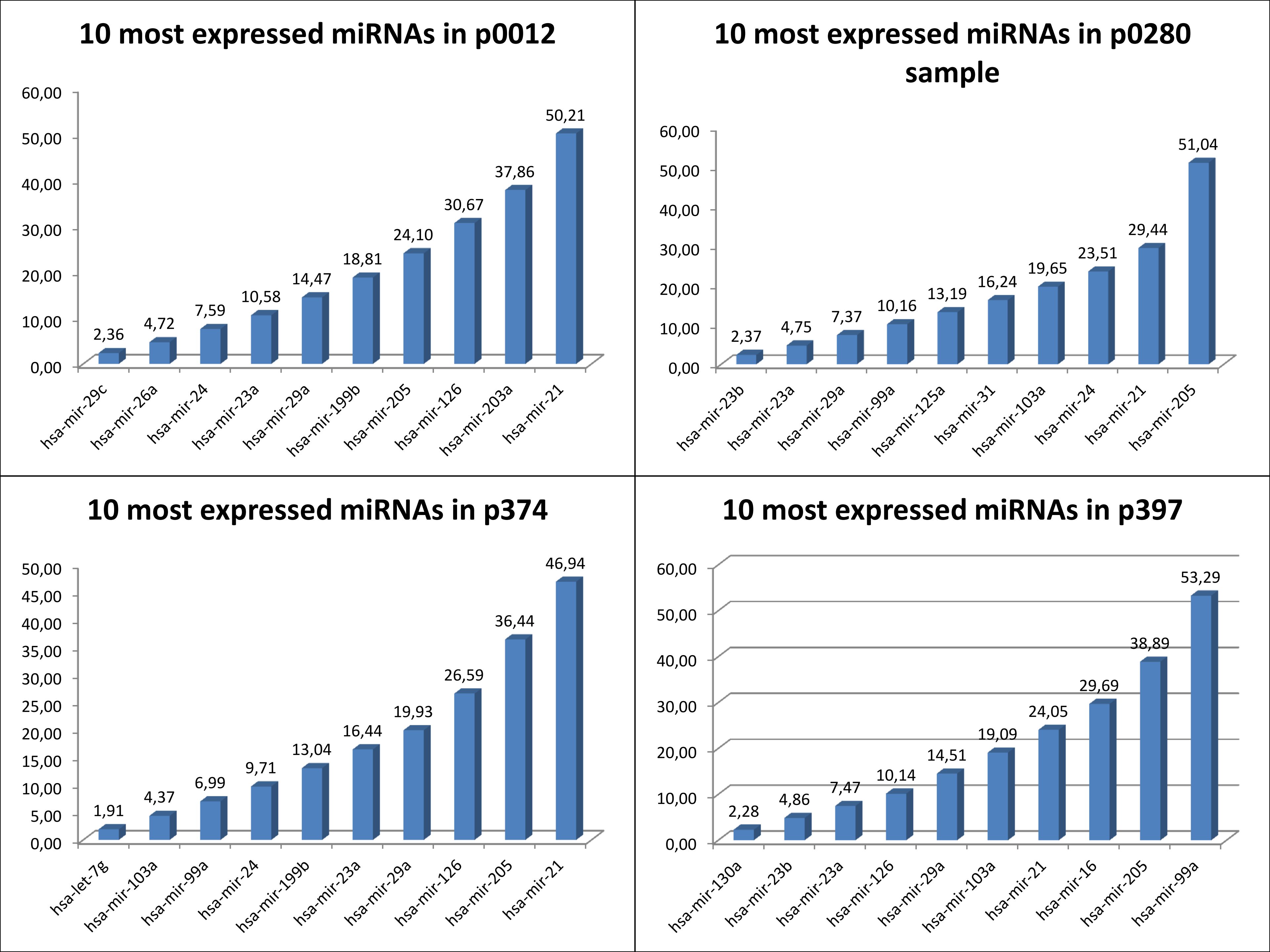
10 most expressed miRNAs in p0012
60,00
50,00
40,00
30,00
20,00
10,00
0,00
2,36
4,72
7,59
10,58
14,47
18,81
24,10
30,67
37,86
50,21
hsa-mir-29c
hsa-mir-26a
hsa-mir-24
hsa-mir-23a
hsa-mir-29a
hsa-mir-199b
hsa-mir-205
hsa-mir-126
hsa-mir-203a
hsa-mir-21
10 most expressed miRNAs in p0280 sample
60,00
50,00
40,00
30,00
20,00
10,00
0,00
2,37
4,75
7,37
10,16
13,19
16,24
19,65
23,51
29,44
51,04
hsa-mir-23b
hsa-mir-23a
hsa-mir-29a
hsa-mir-99a
hsa-mir-125a
hsa-mir-31
hsa-mir-103a
hsa-mir-24
hsa-mir-21
hsa-mir-205
10 most expressed miRNAs in p374
50,00
45,00
40,00
35,00
30,00
25,00
20,00
15,00
10,00
5,00
0,00
1,91
4,37
6,99
9,71
13,04
16,44
19,93
26,59
36,44
46,94
hsa-let-7g
hsa-mir-103a
hsa-mir-99a
hsa-mir-24
hsa-mir-199b
hsa-mir-23a
hsa-mir-29a
hsa-mir-126
hsa-mir-205
hsa-mir-21
10 most expressed miRNAs in p397
60,00
50,00
40,00
30,00
20,00
10,00
0,00
2,28
4,86
7,47
10,14
14,51
19,09
24,05
29,69
38,89
53,29
hsa-mir-130a
hsa-mir-23b
hsa-mir-23a
hsa-mir-126
hsa-mir-29a
hsa-mir-103a
hsa-mir-21
hsa-mir-16
hsa-mir-205
hsa-mir-99a

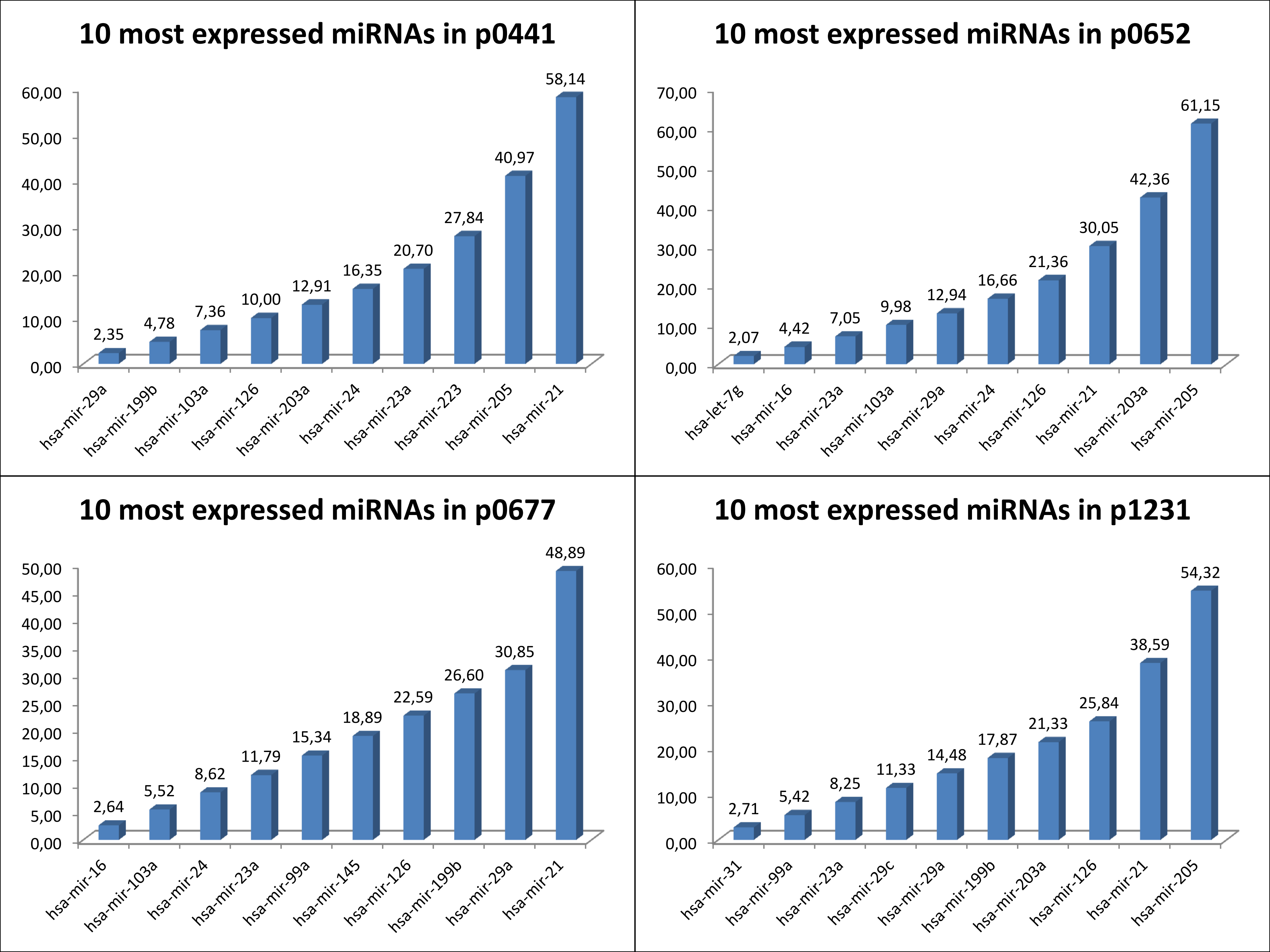
10 most expressed miRNAs in p0441
0,00
10,00
20,00
30,00
40,00
50,00
60,00
2,35
4,78
7,36
10,00
12,91
16,35
20,70
27,84
40,97
58,14
hsa-mir-29a
hsa-mir-199b
hsa-mir-103a
hsa-mir-126
hsa-mir-203a
hsa-mir-24
hsa-mir-23a
hsa-mir-223
hsa-mir-205
hsa-mir-21
10 most expressed miRNAs in p0652
0,00
10,00
20,00
30,00
40,00
50,00
60,00
70,00
2,07
4,42
7,05
9,98
12,94
16,66
21,36
30,05
42,36
61,15
hsa-let-7g
hsa-mir-16
hsa-mir-23a
hsa-mir-103a
hsa-mir-29a
hsa-mir-24
hsa-mir-126
hsa-mir-21
hsa-mir-203a
hsa-mir-205
10 most expressed miRNAs in p0677
0,00
5,00
10,00
15,00
20,00
25,00
30,00
35,00
40,00
45,00
50,00
2,64
5,52
8,62
11,79
15,34
18,89
22,59
26,60
30,85
48,89
hsa-mir-16
hsa-mir-103a
hsa-mir-24
hsa-mir-23a
hsa-mir-99a
hsa-mir-145
hsa-mir-126
hsa-mir-199b
hsa-mir-29a
hsa-mir-21
10 most expressed miRNAs in p1231
0,00
10,00
20,00
30,00
40,00
50,00
60,00
2,71
5,42
8,25
11,33
14,48
17,87
21,33
25,84
38,59
54,32
hsa-mir-31
hsa-mir-99a
hsa-mir-23a
hsa-mir-29c
hsa-mir-29a
hsa-mir-199b
hsa-mir-203a
hsa-mir-126
hsa-mir-21
hsa-mir-205

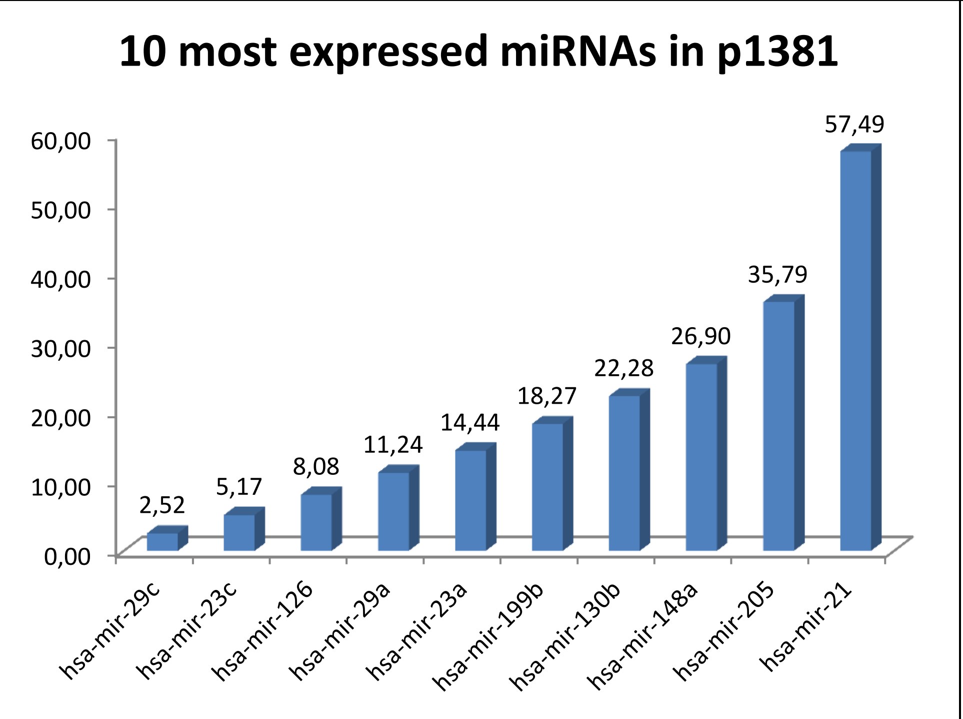
10 most expressed miRNAs in p1381
60,00
50,00
40,00
30,00
20,00
10,00
0,00
2,52
5,17
8,08
11,24
14,44
18,27
22,28
26,90
35,79
57,49
hsa-mir-29c
hsa-mir-23c
hsa-mir-126
hsa-mir-29a
hsa-mir-23a
hsa-mir-199b
hsa-mir-130b
hsa-mir-148a
hsa-mir-205
hsa-mir-21

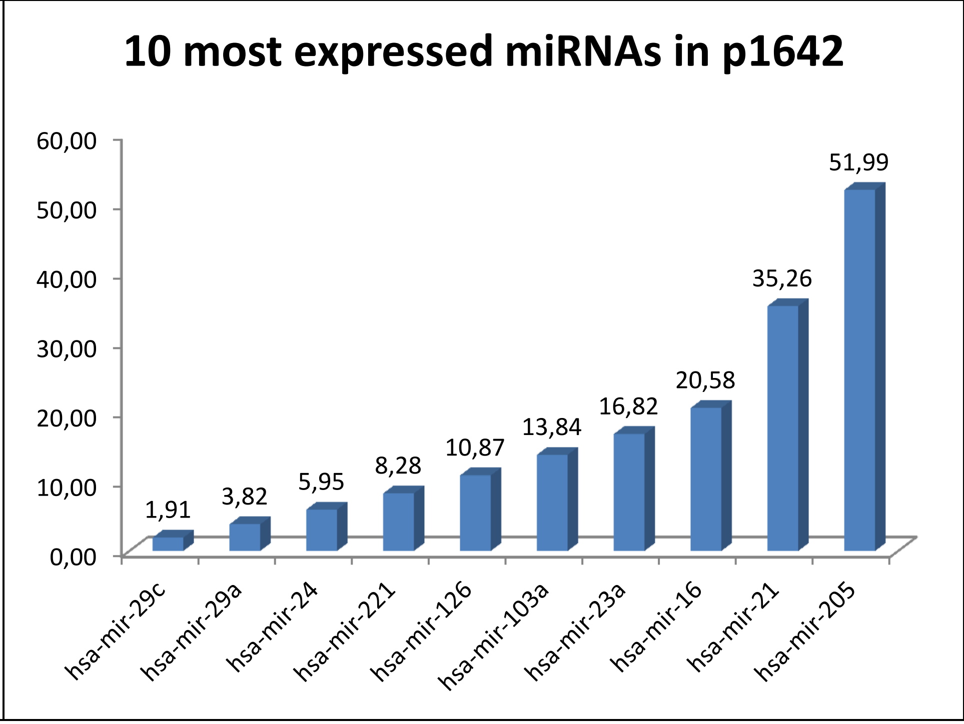
10 most expressed miRNAs in p1642
60,00
50,00
40,00
30,00
20,00
10,00
0,00
1,91
3,82
5,95
8,28
10,87
13,84
16,82
20,58
35,26
51,99
hsa-mir-29c
hsa-mir-29a
hsa-mir-24
hsa-mir-221
hsa-mir-126
hsa-mir-103a
hsa-mir-23a
hsa-mir-16
hsa-mir-21
hsa-mir-205
