# Supplementary figures and images for: Small RNAs in metastatic and non-metastatic oral squamous cell carcinoma
Source: BMC Med Genomics. 2015 Jun 24;8:31. doi: 10.1186/s12920-015-0102-4 (PMC4479233; doi:10.1186/s12920-015-0102-4)

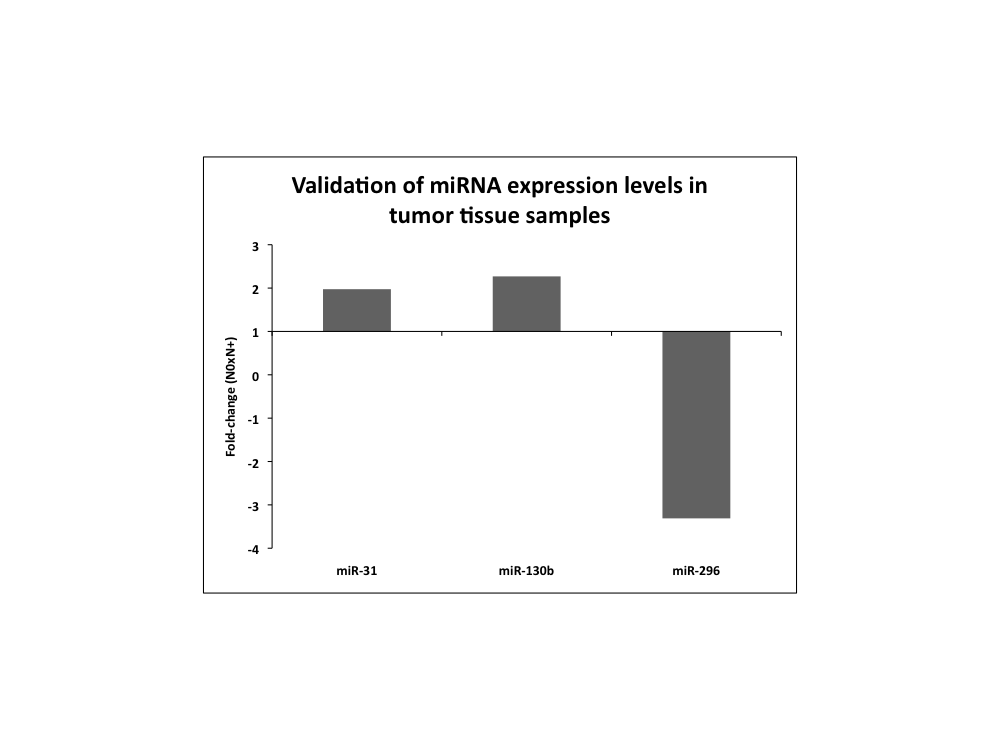

Supplement: Additional file 6: — Validation of miRNA expression levels in an additional set of tumor tissue samples. Average expression levels were considered in this figure and fold-change compares N0 and N+ samples. Negative results indicate over-expression in N+ samples. [file 12920_2015_102_MOESM6_ESM.tiff]
