# Supplementary material for: Small RNAs in metastatic and non-metastatic oral squamous cell carcinoma
Source: BMC Med Genomics. 2015 Jun 24;8:31. doi: 10.1186/s12920-015-0102-4 (PMC4479233; doi:10.1186/s12920-015-0102-4)
Supplement: Additional file 7: — Complete set of small RNAs other than miRNA identified in tumor samples, with specific type report in non-coding RNA databanks. For the annotation of small RNAs we used BLAST search against available databanks of non-coding RNA sequences. [file 12920_2015_102_MOESM7_ESM.pdf]

Additional File 7:

pasRNA						
**QryName**	**Database**	**Type**	**Evalue**	**Qry Cov**	**Sbj Cov**	**Identity**
11695 chromosome 10: 3214886 - 3214921	deepbase_v1.2	pasRNA	1.1e-07	75	100	100
11896 chromosome 20: 33292111 - 33292146	deepbase_v1.2	pasRNA	1.1e-05	66	100	100
17551 chromosome 16: 66586389 - 66586424	deepbase_v1.2	pasRNA	5.3e-07	72	100	100
	deepbase_v1.2	pasRNA	5.3e-07	72	100	100
22537 chromosome 12: 98910173 - 98910208	deepbase_v1.2	pasRNA	2.9e-13	97	100	100
2282 chromosome X: 117479814 - 117479849	deepbase_v1.2	pasRNA	2.4e-06	69	100	100
17170 chromosome 14: 64194739 - 64194774	deepbase_v1.2	pasRNA	5.1e-05	63	100	100
	deepbase_v1.2	pasRNA	2.4e-06	69	100	100
7580 chromosome 1: 180123773 - 180123808	deepbase_v1.2	pasRNA	5.1e-05	63	100	100
**easRNA**						
**QryName**	**Database**	**Type**	**Evalue**	**Qry Cov**	**Sbj Cov**	**Identity**
11719 chromosome 10: 32298282 - 32298317	deepbase_v1.2	easRNA	5.3e-07	72	100	100
15645 chromosome 17: 54672051 - 54672087	deepbase_v1.2	easRNA	1.1e-05	66	100	100
19996 chromosome 8: 81153691 - 81153726	deepbase_v1.2	easRNA	4.7e-13	100	70	100
4479 chromosome 9: 136629173 - 136629208	deepbase_v1.2	easRNA	1.1e-07	75	100	100
5159 chromosome 8: 144660317 - 144660352	deepbase_v1.2	easRNA	5.1e-05	63	100	100
570 chromosome 5: 10394191 - 10394227	deepbase_v1.2	easRNA	1.1e-07	75	100	100
5741 chromosome 7: 151163616 - 151163652	deepbase_v1.2	easRNA	2.4e-06	69	100	100
7421 chromosome 5: 177631825 - 177631860	deepbase_v1.2	easRNA	2.4e-06	69	100	100
7799 chromosome 19: 18418328 - 18418363	deepbase_v1.2	easRNA	5.3e-07	72	100	100
8117 chromosome 10: 18964393 - 18964428	deepbase_v1.2	easRNA	2.4e-06	69	100	100
**rasRNA**						

QryName	Database	Type	Evalue	Qry Cov	Sbj Cov	Identity
10073 chromosome 19: 24185779 - 24185814	deepbase_v1.2	rasRNA	2.4e-08	77	100	100
1157 chromosome X: 108297800 - 108297835	deepbase_v1.2	rasRNA	9.4e-10	83	100	100
11937 chromosome 17: 33478243 - 33478278	deepbase_v1.2	rasRNA	2.8e-11	88	100	100
14495 chromosome X: 47453337 - 47453372	deepbase_v1.2	rasRNA	5.1e-05	63	100	100
17878 chromosome 6: 68597690 - 68597725	deepbase_v1.2	rasRNA	1.1e-05	66	100	100
21781 chromosome 13: 93142439 - 93142474	deepbase_v1.2	rasRNA	5.1e-05	63	100	100
3483 chromosome 12: 127650892 - 127650927	deepbase_v1.2	rasRNA	7.2e-14	100	100	100
	deepbase_v1.2	rasRNA	3.5e-13	100	76	100
5520 chromosome 7: 148638632 - 148638675	deepbase_v1.2	rasRNA	1.7e-10	86	100	100
6196 chromosome 19: 1574499 - 1574534	deepbase_v1.2	rasRNA	2.4e-06	69	100	100
8094 chromosome 2: 189189558 - 189189593	deepbase_v1.2	rasRNA	2.4e-06	69	100	100
8493 chromosome 2: 198099548 - 198099583	deepbase_v1.2	rasRNA	4.8e-09	80	100	100
**nasRNA**						
**QryName**	**Database**	**Type**	**Evalue**	**Qry Cov**	**Sbj Cov**	**Identity**
13687 chromosome 22: 43011319 - 43011354	deepbase_v1.2	nasRNA	7.2e-14	100	100	100
19109 chromosome 14: 76070569 - 76070604	deepbase_v1.2	nasRNA	7.2e-14	100	100	100
	deepbase_v1.2	nasRNA	7.2e-14	100	100	100
	deepbase_v1.2	nasRNA	7.2e-14	100	100	100
	deepbase_v1.2	nasRNA	7.2e-14	100	100	100
769 chromosome 11: 10529817 - 10529860	deepbase_v1.2	nasRNA	4.2e-06	80	100	97
**SnoRNA**						
**QryName**	**Database**	**Type**	**Evalue**	**Qry Cov**	**Sbj Cov**	**Identity**
11629 chromosome 6: 31803072 - 31803107	deepbase_v1.2	snoRNA	1.1e-12	100	40	100
1332 chromosome 1: 109643203 - 109643238	deepbase_v1.2	snoRNA	1.1e-12	100	36	100
16047 chromosome 8: 56986428 - 56986463	deepbase_v1.2	snoRNA	1.0e-12	100	41	100

21116 chromosome 20: 8811963 - 8811998	fRNAdb_v3.4	H/ACA box snoRNA	1.5e-12	100	17	100
22007 chromosome 9: 95054743 - 95054778	deepbase_v1.2	snoRNA	1.3e-12	100	27	100
2942 chromosome 11: 122929679 - 122929714	smiRNAdb_Human_Tratado.fasta	snoRNA	1.1e-05	66	100	100
3542 chromosome 19: 12817263 - 12817298	fRNAdb_v3.4	C/D box snoRNA	8.4e-13	100	51	100
	deepbase_v1.2	snoRNA	1.1e-12	100	40	100
	RNAdb_ALL.fasta	snoRNA	8.3e-13	100	52	100
7203 chromosome 1: 173833966 - 173834001	fRNAdb_v3.4	C/D box snoRNA	7.1e-13	100	58	100
	fRNAdb_v3.4	C/D box snoRNA	9.9e-13	100	43	100
	fRNAdb_v3.4	C/D box snoRNA	9.5e-13	100	46	100
**U6 spliceosomal**						
**QryName**	**Database**	**Type**	**Evalue**	**Qry Cov**	**Sbj Cov**	**Identity**
15541 chromosome 2: 53797551 - 53797586	fRNAdb_v3.4	U6 spliceosomal	1.2e-12	100	33	100
20706 chromosome 15: 85481716 - 85481751	fRNAdb_v3.4	U6 spliceosomal	1.2e-12	100	33	100
5715 chromosome 1: 150995291 - 150995326	fRNAdb_v3.4	U6 spliceosomal	1.2e-12	100	33	100
	fRNAdb_v3.4	U6 spliceosomal	6.1e-10	100	33	97
9957 chromosome 2: 239321299 - 239321334	fRNAdb_v3.4	U6 spliceosomal	1.2e-12	100	35	100
6405 chromosome 17: 16041148 - 16041183	fRNAdb_v3.4	U6 spliceosomal	1.2e-12	100	34	100
**Y_RNA**						
**QryName**	**Database**	**Type**	**Evalue**	**Qry Cov**	**Sbj Cov**	**Identity**
17799 chromosome 16: 68123395 - 68123430	fRNAdb_v3.4	Y RNA	1.1e-12	100	38	100
**piRNA**						
**QryName**	**Database**	**Type**	**Evalue**	**Qry Cov**	**Sbj Cov**	**Identity**
4377 chromosome 18: 13543935 - 13543970	fRNAdb_v3.4	piRNA	1.1e-07	75	100	100
	piRNABank_ALL.fasta	piRNA	1.1e-07	75	100	100
9123 chromosome 18: 21603268 - 21603303	fRNAdb_v3.4	piRNA	4.8e-09	80	100	100
	piRNABank_ALL.fasta	piRNA	4.8e-09	80	100	100

easRNA, pasRNA, rasRNA, nasRNA						
**QryName**	**Database**	**Type**	**Evalue**	**Qry Cov**	**Sbj Cov**	**Identity**
10517 chromosome X: 26029858 - 26029893	deepbase_v1.2	easRNA	5.3e-07	72	100	100
	deepbase_v1.2	rasRNA	5.3e-07	72	100	100
10633 chromosome 17: 26662603 - 26662638	deepbase_v1.2	easRNA	1.1e-07	75	100	100
	deepbase_v1.2	pasRNA	1.1e-07	75	100	100
10701 chromosome 17: 27050704 - 27050739	deepbase_v1.2	easRNA	2.9e-13	100	80	100
11055 chromosome 1: 28833876 - 28833911	deepbase_v1.2	nasRNA	7.2e-14	100	100	100
	deepbase_v1.2	rasRNA	7.2e-14	100	100	100
11135 chromosome 17: 29298071 - 29298106	deepbase_v1.2	easRNA	5.3e-07	72	100	100
	deepbase_v1.2	pasRNA	5.3e-07	72	100	100
11198 chromosome 22: 29729165 - 29729200	deepbase_v1.2	easRNA	8.8e-13	100	49	100
	deepbase_v1.2	easRNA	9.0e-13	100	48	100
	deepbase_v1.2	nasRNA	8.8e-13	100	49	100
	deepbase_v1.2	pasRNA	8.8e-13	100	49	100
11199 chromosome 22: 29729187 - 29729222	deepbase_v1.2	easRNA	8.8e-13	100	49	100
	deepbase_v1.2	easRNA	9.0e-13	100	48	100
	deepbase_v1.2	nasRNA	8.8e-13	100	49	100
	deepbase_v1.2	pasRNA	8.8e-13	100	49	100
13101 chromosome 19: 3978023 - 3978058	deepbase_v1.2	easRNA	1.1e-05	66	100	100
	deepbase_v1.2	easRNA	2.4e-06	69	100	100
	deepbase_v1.2	easRNA	5.1e-05	63	100	100
	deepbase_v1.2	easRNA	1.1e-07	75	100	100
13733 chromosome 7: 43313857 - 43313892	deepbase_v1.2	easRNA	5.3e-07	72	100	100
	deepbase_v1.2	easRNA	5.3e-07	72	100	100
14288 chromosome 21: 46221693 - 46221728	deepbase_v1.2	easRNA	5.3e-07	72	100	100

	deepbase_v1.2	pasRNA	5.3e-07	72	100	100
14940 chromosome 19: 49994164 - 49994199	deepbase_v1.2	easRNA	7.2e-14	100	100	100
	deepbase_v1.2	pasRNA	7.2e-14	100	100	100
	deepbase_v1.2	pasRNA	7.2e-14	100	100	100
	deepbase_v1.2	pasRNA	7.2e-14	100	100	100
16801 chromosome 2: 61765303 - 61765338	deepbase_v1.2	easRNA	5.1e-05	63	100	100
	deepbase_v1.2	pasRNA	5.1e-05	63	100	100
16935 chromosome 11: 62621145 - 62621180	deepbase_v1.2	easRNA	7.2e-14	100	100	100
	deepbase_v1.2	nasRNA	7.2e-14	100	100	100
	deepbase_v1.2	pasRNA	7.2e-14	100	100	100
	deepbase_v1.2	pasRNA	7.2e-14	100	100	100
17156 chromosome 11: 64126641 - 64126676	deepbase_v1.2	easRNA	5.1e-05	63	100	100
	deepbase_v1.2	pasRNA	5.1e-05	63	100	100
5101 chromosome 11: 14380686 - 14380721	deepbase_v1.2	easRNA	5.3e-07	72	100	100
	deepbase_v1.2	pasRNA	5.3e-07	72	100	100
185 chromosome 3: 101280735 - 101280770	deepbase_v1.2	easRNA	1.1e-05	66	100	100
	deepbase_v1.2	pasRNA	1.1e-05	66	100	100
5930 chromosome X: 153657094 - 153657129	deepbase_v1.2	easRNA	1.1e-05	66	100	100
	deepbase_v1.2	pasRNA	1.1e-05	66	100	100
7177 chromosome 17: 1733301 - 1733336	deepbase_v1.2	easRNA	2.4e-08	77	100	100
	deepbase_v1.2	pasRNA	2.4e-08	77	100	100
	deepbase_v1.2	pasRNA	2.4e-08	77	100	100
7955 chromosome 3: 186504463 - 186504498	deepbase_v1.2	easRNA	2.5e-13	100	81	100
	deepbase_v1.2	easRNA	7.2e-14	100	100	100
	deepbase_v1.2	nasRNA	7.2e-14	100	100	100
	deepbase_v1.2	pasRNA	7.2e-14	100	100	100

7962 chromosome 3: 186505199 - 186505237	deepbase_v1.2	pasRNA	5.3e-07	72	100	100
	deepbase_v1.2	pasRNA	5.3e-07	72	100	100
8820 chromosome 2: 207026638 - 207026673	deepbase_v1.2	easRNA	7.2e-14	100	100	100
	deepbase_v1.2	nasRNA	7.2e-14	100	100	100
	deepbase_v1.2	pasRNA	7.2e-14	100	100	100
	deepbase_v1.2	pasRNA	7.2e-14	100	100	100
