# Supplementary material for: Small RNAs in metastatic and non-metastatic oral squamous cell carcinoma
Source: BMC Med Genomics. 2015 Jun 24;8:31. doi: 10.1186/s12920-015-0102-4 (PMC4479233; doi:10.1186/s12920-015-0102-4)
Supplement: Additional file 8: — Complete set of small RNAs other than miRNA identified in tumor samples with evidences from ab initio prediction reported in non-coding RNA databanks. The annotation procedure of this set of small RNAs used BLAST search against available databanks of non-coding RNA sequences but reports showed only evidences from ab initio prediction. [file 12920_2015_102_MOESM8_ESM.pdf]

Additional File 8:

QryName	IDncRNAdb	SbjName	SbjHeader	Database	Type	E value	Qry Cov	Sbj Cov	Identity
10074 chromosome 19: 24185867 - 24185902	13210173	IDncRNAdb13210173	>FR0314379|Putative conserved noncoding region (RNAz) Hsapiens	fRNAdb_v3.4	Ab initio prediction	1.4e-12	100	22	100
	8447736	IDncRNAdb8447736	>gnl|Rnaz|RNZ17242 159	RNAdb_ALL.fasta	Ab initio prediction	1.4e-12	100	22	100
10396 chromosome 2: 25454991 - 25455026	13201097	IDncRNAdb13201097	>FR0284974|Putative conserved noncoding region (RNAz) Hsapiens	fRNAdb_v3.4	Ab initio prediction	1.7e-12	100	8	100
	8448572	IDncRNAdb8448572	>gnl|Rnaz|RNZ18002 446	RNAdb_ALL.fasta	Ab initio prediction	1.7e-12	100	8	100
10567 chromosome 1: 26253801 - 26253836	13182208	IDncRNAdb13182208	>FR0223937|Putative conserved noncoding region (RNAz) Hsapiens	fRNAdb_v3.4	Ab initio prediction	1.4e-12	100	21	100
	8444103	IDncRNAdb8444103	>gnl|Rnaz|RNZ1394 167	RNAdb_ALL.fasta	Ab initio prediction	1.4e-12	100	21	100
10872 chromosome 1: 27929061 - 27929096	13138018	IDncRNAdb13138018	>FR0081001|Putative conserved noncoding region (RNAz) Hsapiens	fRNAdb_v3.4	Ab initio prediction	1.7e-12	100	5	100
	8445126	IDncRNAdb8445126	>gnl|Rnaz|RNZ1487 707	RNAdb_ALL.fasta	Ab initio prediction	1.7e-12	100	5	100
10983 chromosome 3: 2843082 - 2843117	13223817	IDncRNAdb13223817	>FR0358037|Putative conserved noncoding region (RNAz) Hsapiens	fRNAdb_v3.4	Ab initio prediction	1.4e-12	100	22	100
	8453348	IDncRNAdb8453348	>gnl|Rnaz|RNZ22344 160	RNAdb_ALL.fasta	Ab initio prediction	1.4e-12	100	22	100
11420 chromosome 19: 30832066 - 30832101	13215641	IDncRNAdb13215641	>FR0332043|Putative conserved noncoding region (RNAz) Hsapiens	fRNAdb_v3.4	Ab initio prediction	1.4e-12	100	22	100
	8447767	IDncRNAdb8447767	>gnl|Rnaz|RNZ17270 160	RNAdb_ALL.fasta	Ab initio prediction	1.4e-12	100	22	100
11425 chromosome 19: 30860748 - 30860783	13204761	IDncRNAdb13204761	>FR0296744|Putative conserved noncoding region (RNAz) Hsapiens	fRNAdb_v3.4	Ab initio prediction	1.3e-12	100	30	100
	8447770	IDncRNAdb8447770	>gnl|Rnaz|RNZ17273 120	RNAdb_ALL.fasta	Ab initio prediction	1.3e-12	100	30	100
11881 chromosome 6: 33175618 - 33175653	13115906	IDncRNAdb13115906	>FR0009883|Putative conserved noncoding region (EvoFold) Hsapiens	fRNAdb_v3.4	Ab initio prediction	1.0e-12	100	41	100
	8190499	IDncRNAdb8190499	>gnl|evofold|EVF19085 87	RNAdb_ALL.fasta	Ab initio prediction	1.0e-12	100	41	100
11923 chromosome 6: 33419940 - 33419975	13118727	IDncRNAdb13118727	>FR0018877|Putative conserved noncoding region (RNAz) Hsapiens	fRNAdb_v3.4	Ab initio prediction	1.8e-12	100	4	100
	8460741	IDncRNAdb8460741	>gnl|Rnaz|RNZ29065 782	RNAdb_ALL.fasta	Ab initio prediction	1.8e-12	100	4	100
12006 chromosome 17: 33884684 - 33884719	13125269	IDncRNAdb13125269	>FR0039648|Putative conserved noncoding region (RNAz) Hsapiens	fRNAdb_v3.4	Ab initio prediction	1.6e-12	100	15	100

<table>
<tr><td></td><td>8445415</td><td>IDncRNAdb8445415</td><td>>gnl|Rnaz|RNZ15132 240</td><td>RNAdb_ALL.fasta</td><td>Ab initio prediction</td><td>1.6e-12</td><td>100</td><td>15</td><td>100</td></tr>
<tr><td rowspan="2">12677 chromosome 15: 37388498 - 37388533</td><td>13238165</td><td>IDncRNAdb13238165</td><td>>FR0404424|Putative conserved noncoding region (EvoFold) Hsapiens</td><td>fRNAdb_v3.4</td><td>Ab initio prediction</td><td>1.2e-12</td><td>100</td><td>32</td><td>100</td></tr>
<tr><td>8212543</td><td>IDncRNAdb8212543</td><td>>gnl|evofold|EVF39125 111</td><td>RNAdb_ALL.fasta</td><td>Ab initio prediction</td><td>1.2e-12</td><td>100</td><td>32</td><td>100</td></tr>
<tr><td rowspan="2">12750 chromosome 6: 37693032 - 37693067</td><td>13224272</td><td>IDncRNAdb13224272</td><td>>FR0359449|Putative conserved noncoding region (RNAz) Hsapiens</td><td>fRNAdb_v3.4</td><td>Ab initio prediction</td><td>1.4e-12</td><td>100</td><td>25</td><td>100</td></tr>
<tr><td>8460808</td><td>IDncRNAdb8460808</td><td>>gnl|Rnaz|RNZ29126 143</td><td>RNAdb_ALL.fasta</td><td>Ab initio prediction</td><td>1.4e-12</td><td>100</td><td>25</td><td>100</td></tr>
<tr><td rowspan="2">1283 chromosome X: 109298576 - 109298611</td><td>13169680</td><td>IDncRNAdb13169680</td><td>>FR0183135|Putative conserved noncoding region (EvoFold) Hsapiens</td><td>fRNAdb_v3.4</td><td>Ab initio prediction</td><td>9.3e-13</td><td>100</td><td>46</td><td>100</td></tr>
<tr><td>8200854</td><td>IDncRNAdb8200854</td><td>>gnl|evofold|EVF28499 77</td><td>RNAdb_ALL.fasta</td><td>Ab initio prediction</td><td>9.2e-13</td><td>100</td><td>47</td><td>100</td></tr>
<tr><td rowspan="2">12947 chromosome 20: 38792900 - 38792935</td><td>13129535</td><td>IDncRNAdb13129535</td><td>>FR0053445|Putative conserved noncoding region (RNAz) Hsapiens</td><td>fRNAdb_v3.4</td><td>Ab initio prediction</td><td>1.6e-12</td><td>100</td><td>12</td><td>100</td></tr>
<tr><td>8452357</td><td>IDncRNAdb8452357</td><td>>gnl|Rnaz|RNZ21443 279</td><td>RNAdb_ALL.fasta</td><td>Ab initio prediction</td><td>1.6e-12</td><td>100</td><td>12</td><td>100</td></tr>
<tr><td rowspan="2">13390 chromosome 17: 41215797 - 41215832</td><td>13115650</td><td>IDncRNAdb13115650</td><td>>FR0009045|Putative conserved noncoding region (RNAz) Hsapiens</td><td>fRNAdb_v3.4</td><td>Ab initio prediction</td><td>1.1e-12</td><td>100</td><td>38</td><td>100</td></tr>
<tr><td>8445735</td><td>IDncRNAdb8445735</td><td>>gnl|Rnaz|RNZ15423 93</td><td>RNAdb_ALL.fasta</td><td>Ab initio prediction</td><td>1.1e-12</td><td>100</td><td>39</td><td>100</td></tr>
<tr><td rowspan="2">13566 chromosome 2: 42255151 - 42255186</td><td>13119579</td><td>IDncRNAdb13119579</td><td>>FR0021473|Putative conserved noncoding region (RNAz) Hsapiens</td><td>fRNAdb_v3.4</td><td>Ab initio prediction</td><td>1.1e-12</td><td>100</td><td>36</td><td>100</td></tr>
<tr><td>8448787</td><td>IDncRNAdb8448787</td><td>>gnl|Rnaz|RNZ18198 100</td><td>RNAdb_ALL.fasta</td><td>Ab initio prediction</td><td>1.1e-12</td><td>100</td><td>36</td><td>100</td></tr>
<tr><td rowspan="2">1402 chromosome X: 110187501 - 110187536</td><td>13200599</td><td>IDncRNAdb13200599</td><td>>FR0283330|Putative conserved noncoding region (RNAz) Hsapiens</td><td>fRNAdb_v3.4</td><td>Ab initio prediction</td><td>1.5e-12</td><td>100</td><td>18</td><td>100</td></tr>
<tr><td>8468695</td><td>IDncRNAdb8468695</td><td>>gnl|Rnaz|RNZ36296 197</td><td>RNAdb_ALL.fasta</td><td>Ab initio prediction</td><td>1.5e-12</td><td>100</td><td>18</td><td>100</td></tr>
<tr><td rowspan="2">14322 chromosome 19: 46440705 - 46440740</td><td>13164858</td><td>IDncRNAdb13164858</td><td>>FR0167298|Putative conserved noncoding region (RNAz) Hsapiens</td><td>fRNAdb_v3.4</td><td>Ab initio prediction</td><td>1.7e-12</td><td>100</td><td>9</td><td>100</td></tr>
<tr><td>8448049</td><td>IDncRNAdb8448049</td><td>>gnl|Rnaz|RNZ17527 388</td><td>RNAdb_ALL.fasta</td><td>Ab initio prediction</td><td>1.7e-12</td><td>100</td><td>9</td><td>100</td></tr>
<tr><td rowspan="2">14548 chromosome 1: 47697993 - 47698028</td><td>13197966</td><td>IDncRNAdb13197966</td><td>>FR0274749|Putative conserved noncoding region (RNAz) Hsapiens</td><td>fRNAdb_v3.4</td><td>Ab initio prediction</td><td>1.5e-12</td><td>100</td><td>19</td><td>100</td></tr>
<tr><td>8451495</td><td>IDncRNAdb8451495</td><td>>gnl|Rnaz|RNZ2066 186</td><td>RNAdb_ALL.fasta</td><td>Ab initio prediction</td><td>1.5e-12</td><td>100</td><td>19</td><td>100</td></tr>
<tr><td rowspan="2">14693 chromosome 18: 4847267 - 4847302</td><td>13225272</td><td>IDncRNAdb13225272</td><td>>FR0362817|Putative conserved noncoding region (RNAz) Hsapiens</td><td>fRNAdb_v3.4</td><td>Ab initio prediction</td><td>1.4e-12</td><td>100</td><td>22</td><td>100</td></tr>
<tr><td>8446665</td><td>IDncRNAdb8446665</td><td>>gnl|Rnaz|RNZ16269 157</td><td>RNAdb_ALL.fasta</td><td>Ab initio prediction</td><td>1.4e-12</td><td>100</td><td>23</td><td>100</td></tr>
<tr><td>14743 chromosome X: 48815192 -</td><td>13214756</td><td>IDncRNAdb132</td><td>>FR0329270|Putative conserved noncoding region</td><td>fRNAdb_v3.4</td><td>Ab initio</td><td>1.4e-12</td><td>100</td><td>24</td><td>100</td></tr>
</table>

<table>
<tr><td rowspan="2">48815227</td><td></td><td>14756</td><td>(RNAz) Hsapiens</td><td></td><td>prediction</td><td></td><td></td><td></td><td></td></tr>
<tr><td>8467972</td><td>IDncRNAdb8467972</td><td>>gnl|Rnaz|RNZ35639 149</td><td>RNAdb_ALL.fasta</td><td>Ab initio prediction</td><td>1.4e-12</td><td>100</td><td>24</td><td>100</td></tr>
<tr><td>15925 chromosome 17: 56408644 - 56408679</td><td>13183493</td><td>IDncRNAdb13183493</td><td>>FR0228241|Putative conserved noncoding region (RNAz) Hsapiens</td><td>fRNAdb_v3.4</td><td>Ab initio prediction</td><td>1.1e-12</td><td>100</td><td>36</td><td>100</td></tr>
<tr><td rowspan="2">16106 chromosome 14: 57321252 - 57321287</td><td>13133708</td><td>IDncRNAdb13133708</td><td>>FR0066893|Putative conserved noncoding region (RNAz) Hsapiens</td><td>fRNAdb_v3.4</td><td>Ab initio prediction</td><td>1.4e-12</td><td>100</td><td>22</td><td>100</td></tr>
<tr><td>8441195</td><td>IDncRNAdb8441195</td><td>>gnl|Rnaz|RNZ11296 159</td><td>RNAdb_ALL.fasta</td><td>Ab initio prediction</td><td>1.4e-12</td><td>100</td><td>22</td><td>100</td></tr>
<tr><td>16118 chromosome 11: 57408685 - 57408720</td><td>8205500</td><td>IDncRNAdb8205500</td><td>>gnl|evofold|EVF32722 85</td><td>RNAdb_ALL.fasta</td><td>Ab initio prediction</td><td>1.0e-12</td><td>100</td><td>42</td><td>100</td></tr>
<tr><td>16207 chromosome 17: 57918633 - 57918668</td><td>8217669</td><td>IDncRNAdb8217669</td><td>>gnl|evofold|EVF43785 72</td><td>RNAdb_ALL.fasta</td><td>Ab initio prediction</td><td>8.6e-13</td><td>100</td><td>50</td><td>100</td></tr>
<tr><td rowspan="2">1708 chromosome 8: 112453349 - 112453386</td><td>13221956</td><td>IDncRNAdb13221956</td><td>>FR0352105|Putative conserved noncoding region (RNAz) Hsapiens</td><td>fRNAdb_v3.4</td><td>Ab initio prediction</td><td>1.4e-12</td><td>100</td><td>24</td><td>100</td></tr>
<tr><td>8465166</td><td>IDncRNAdb8465166</td><td>>gnl|Rnaz|RNZ33088 150</td><td>RNAdb_ALL.fasta</td><td>Ab initio prediction</td><td>1.4e-12</td><td>100</td><td>24</td><td>100</td></tr>
<tr><td rowspan="2">17625 chromosome 11: 67134690 - 67134725</td><td>13181509</td><td>IDncRNAdb13181509</td><td>>FR0221692|Putative conserved noncoding region (RNAz) Hsapiens</td><td>fRNAdb_v3.4</td><td>Ab initio prediction</td><td>1.5e-12</td><td>100</td><td>15</td><td>100</td></tr>
<tr><td>8472866</td><td>IDncRNAdb8472866</td><td>>gnl|Rnaz|RNZ7113 233</td><td>RNAdb_ALL.fasta</td><td>Ab initio prediction</td><td>1.5e-12</td><td>100</td><td>15</td><td>100</td></tr>
<tr><td rowspan="3">18109 chromosome 7: 70038210 - 70038245</td><td>13237393</td><td>IDncRNAdb13237393</td><td>>FR0401934|Putative conserved noncoding region (EvoFold) Hsapiens</td><td>fRNAdb_v3.4</td><td>Ab initio prediction</td><td>1.1e-12</td><td>100</td><td>36</td><td>100</td></tr>
<tr><td>8193605</td><td>IDncRNAdb8193605</td><td>>gnl|evofold|EVF21909 98</td><td>RNAdb_ALL.fasta</td><td>Ab initio prediction</td><td>1.1e-12</td><td>100</td><td>37</td><td>100</td></tr>
<tr><td>239176</td><td>IDncRNAdb239176</td><td>>CRCNEAC00029107_Human</td><td>Condor_ALL_Org_Annotation.fasta</td><td>ncRNA transcript</td><td>1.4e-12</td><td>100</td><td>23</td><td>100</td></tr>
<tr><td rowspan="2">18122 chromosome 3: 70125795 - 70125831</td><td>13201103</td><td>IDncRNAdb13201103</td><td>>FR0284996|Putative conserved noncoding region (EvoFold) Hsapiens</td><td>fRNAdb_v3.4</td><td>Ab initio prediction</td><td>1.3e-12</td><td>100</td><td>27</td><td>100</td></tr>
<tr><td>8181763</td><td>IDncRNAdb8181763</td><td>>gnl|evofold|EVF11143 130</td><td>RNAdb_ALL.fasta</td><td>Ab initio prediction</td><td>1.3e-12</td><td>100</td><td>27</td><td>100</td></tr>
<tr><td>18157 chromosome 15: 70391562 - 70391597</td><td>8442889</td><td>IDncRNAdb8442889</td><td>>gnl|Rnaz|RNZ12836 461</td><td>RNAdb_ALL.fasta</td><td>Ab initio prediction</td><td>1.7e-12</td><td>100</td><td>7</td><td>100</td></tr>
<tr><td rowspan="2">18160 chromosome 17: 704270 - 704306</td><td>13119157</td><td>IDncRNAdb13119157</td><td>>FR0020206|Putative conserved noncoding region (EvoFold) Hsapiens</td><td>fRNAdb_v3.4</td><td>Ab initio prediction</td><td>2.4e-06</td><td>69</td><td>100</td><td>100</td></tr>
<tr><td>8216253</td><td>IDncRNAdb8216253</td><td>>gnl|evofold|EVF42498 25</td><td>RNAdb_ALL.fasta</td><td>Ab initio prediction</td><td>1.1e-05</td><td>66</td><td>100</td><td>100</td></tr>
<tr><td rowspan="2">18336 chromosome 3: 71591156 - 71591193</td><td>13141868</td><td>IDncRNAdb13141868</td><td>>FR0093293|Putative conserved noncoding region (RNAz) Hsapiens</td><td>fRNAdb_v3.4</td><td>Ab initio prediction</td><td>1.2e-12</td><td>100</td><td>32</td><td>100</td></tr>
<tr><td>8454353</td><td>IDncRNAdb8454353</td><td>>gnl|Rnaz|RNZ23258 110</td><td>RNAdb_ALL.fasta</td><td>Ab initio prediction</td><td>1.2e-12</td><td>100</td><td>33</td><td>100</td></tr>
<tr><td>18442 chromosome 3: 72221386 - 72221422</td><td>13134147</td><td>IDncRNAdb13134147</td><td>>FR0068415|Putative conserved noncoding region (RNAz) Hsapiens</td><td>fRNAdb_v3.4</td><td>Ab initio prediction</td><td>1.4e-12</td><td>100</td><td>20</td><td>100</td></tr>
</table>

	8454379	IDncRNAdb8454379	>gnl|Rnaz|RNZ23281 177	RNAdb_ALL.fasta	Ab initio prediction	1.4e-12	100	20	100
18526 chromosome 3: 72817283 - 72817318	13125634	IDncRNAdb13125634	>FR0040905|Putative conserved noncoding region (RNAz) Hsapiens	fRNAdb_v3.4	Ab initio prediction	1.6e-12	100	11	100
	8454392	IDncRNAdb8454392	>gnl|Rnaz|RNZ23293 307	RNAdb_ALL.fasta	Ab initio prediction	1.6e-12	100	11	100
18665 chromosome 2: 7350334 - 7350369	13115547	IDncRNAdb13115547	>FR0008727|Putative conserved noncoding region (RNAz) Hsapiens	fRNAdb_v3.4	Ab initio prediction	6.2e-13	100	63	100
	8444025	IDncRNAdb8444025	>gnl|Rnaz|RNZ13869 57	RNAdb_ALL.fasta	Ab initio prediction	5.9e-13	100	64	100
18716 chromosome X: 73834571 - 73834606	13203106	IDncRNAdb13203106	>FR0291402|Putative conserved noncoding region (RNAz) Hsapiens	fRNAdb_v3.4	Ab initio prediction	1.5e-12	100	16	100
	8468331	IDncRNAdb8468331	>gnl|Rnaz|RNZ35965 224	RNAdb_ALL.fasta	Ab initio prediction	1.5e-12	100	16	100
19479 chromosome 15: 78031473 - 78031508	13121632	IDncRNAdb13121632	>FR0027925|Putative conserved noncoding region (RNAz) Hsapiens	fRNAdb_v3.4	Ab initio prediction	1.4e-12	100	22	100
	8443050	IDncRNAdb8443050	>gnl|Rnaz|RNZ12982 159	RNAdb_ALL.fasta	Ab initio prediction	1.4e-12	100	22	100
20187 chromosome 18: 8218318 - 8218353	13132614	IDncRNAdb13132614	>FR0063232|Putative conserved noncoding region (EvoFold) Hsapiens	fRNAdb_v3.4	Ab initio prediction	1.4e-12	100	20	100
	8218302	IDncRNAdb8218302	>gnl|evofold|EVF44360 177	RNAdb_ALL.fasta	Ab initio prediction	1.4e-12	100	20	100
20303 chromosome 5: 82968713 - 82968748	13124042	IDncRNAdb13124042	>FR0035748|Putative conserved noncoding region (RNAz) Hsapiens	fRNAdb_v3.4	Ab initio prediction	1.4e-12	100	22	100
	8458544	IDncRNAdb8458544	>gnl|Rnaz|RNZ27068 163	RNAdb_ALL.fasta	Ab initio prediction	1.4e-12	100	22	100
20488 chromosome 7: 8418219 - 8418254	13126479	IDncRNAdb13126479	>FR0043658|Putative conserved noncoding region (RNAz) Hsapiens	fRNAdb_v3.4	Ab initio prediction	1.4e-12	100	22	100
	8462293	IDncRNAdb8462293	>gnl|Rnaz|RNZ30476 157	RNAdb_ALL.fasta	Ab initio prediction	1.4e-12	100	23	100
20805 chromosome 10: 86151459 - 86151494	13175023	IDncRNAdb13175023	>FR0200510|Putative conserved noncoding region (EvoFold) Hsapiens	fRNAdb_v3.4	Ab initio prediction	5.8e-07	100	14	94
	8187521	IDncRNAdb8187521	>gnl|evofold|EVF16378 253	RNAdb_ALL.fasta	Ab initio prediction	5.8e-07	100	14	94
210 chromosome 17: 1014140 - 1014175	13136161	IDncRNAdb13136161	>FR0075006|Putative conserved noncoding region (RNAz) Hsapiens	fRNAdb_v3.4	Ab initio prediction	1.3e-12	100	25	100
	8444875	IDncRNAdb8444875	>gnl|Rnaz|RNZ14641 139	RNAdb_ALL.fasta	Ab initio prediction	1.3e-12	100	26	100
2116 chromosome 5: 115962268 - 115962303	13181240	IDncRNAdb13181240	>FR0220825|Putative conserved noncoding region (RNAz) Hsapiens	fRNAdb_v3.4	Ab initio prediction	1.6e-12	100	14	100
	8458979	IDncRNAdb8458979	>gnl|Rnaz|RNZ27463 243	RNAdb_ALL.fasta	Ab initio prediction	1.6e-12	100	14	100
21163 chromosome 1: 88450683 -	13142763	IDncRNAdb131	>FR0096188|Putative conserved noncoding region	fRNAdb_v3.4	Ab initio	1.3e-12	100	26	100

88450718		42763	(RNAz) Hsapiens		prediction				
	8458447	IDncRNAdb8458447	>gnl|Rnaz|RNZ2698 135	RNAdb_ALL.fasta	Ab initio prediction	1.3e-12	100	26	100
21184 chromosome 5: 88586421 - 88586456	13117425	IDncRNAdb13117425	>FR0014671|Putative conserved noncoding region (RNAz) Hsapiens	fRNAdb_v3.4	Ab initio prediction	1.5e-12	100	18	100
	8458651	IDncRNAdb8458651	>gnl|Rnaz|RNZ27165 193	RNAdb_ALL.fasta	Ab initio prediction	1.5e-12	100	18	100
22243 chromosome 15: 96949661 - 96949696	13147662	IDncRNAdb13147662	>FR0112154|Putative conserved noncoding region (EvoFold) Hsapiens	fRNAdb_v3.4	Ab initio prediction	4.8e-09	80	100	100
	238781	IDncRNAdb238781	>CRCNEAC00027216_Human	Condor_ALL_Org_Annotation.fasta	ncRNA transcript	1.4e-12	100	20	100
22320 chromosome 12: 97503032 - 97503067	13112996	IDncRNAdb13112996	>FR0000618|Putative conserved noncoding region (RNAz) Hsapiens	fRNAdb_v3.4	Ab initio prediction	1.4e-12	100	23	100
	8475182	IDncRNAdb8475182	>gnl|Rnaz|RNZ9429 152	RNAdb_ALL.fasta	Ab initio prediction	1.4e-12	100	23	100
22595 chromosome 8: 99395031 - 99395066	13211075	IDncRNAdb13211075	>FR0317244|Putative conserved noncoding region (RNAz) Hsapiens	fRNAdb_v3.4	Ab initio prediction	1.3e-12	100	30	100
	8465015	IDncRNAdb8465015	>gnl|Rnaz|RNZ32950 119	RNAdb_ALL.fasta	Ab initio prediction	1.3e-12	100	30	100
2436 chromosome X: 118827625 - 118827660	13178503	IDncRNAdb13178503	>FR0211790| Hsapiens	fRNAdb_v3.4	Ab initio prediction	1.8e-12	100	1	100
2640 chromosome 1: 120396276 - 120396311	13160515	IDncRNAdb13160515	>FR0153307|Putative conserved noncoding region (RNAz) Hsapiens	fRNAdb_v3.4	Ab initio prediction	1.2e-12	100	31	100
	8463892	IDncRNAdb8463892	>gnl|Rnaz|RNZ3193 116	RNAdb_ALL.fasta	Ab initio prediction	1.2e-12	100	31	100
2796 chromosome 4: 121771413 - 121771448	13150244	IDncRNAdb13150244	>FR0120439|Putative conserved noncoding region (RNAz) Hsapiens	fRNAdb_v3.4	Ab initio prediction	1.3e-12	100	26	100
	8455805	IDncRNAdb8455805	>gnl|Rnaz|RNZ24578 134	RNAdb_ALL.fasta	Ab initio prediction	1.3e-12	100	27	100
2877 chromosome 5: 122435527 - 122435562	13128653	IDncRNAdb13128653	>FR0050701|Putative conserved noncoding region (EvoFold) Hsapiens	fRNAdb_v3.4	Ab initio prediction	7.7e-13	100	55	100
	8187331	IDncRNAdb8187331	>gnl|evofold|EVF16205 65	RNAdb_ALL.fasta	Ab initio prediction	7.5e-13	100	56	100
309 chromosome 10: 102194923 - 102194958	13191362	IDncRNAdb13191362	>FR0253700| Hsapiens	fRNAdb_v3.4	ncRNA	8.9e-10	100	1	97
3313 chromosome 11: 126148420 - 126148455	13170877	IDncRNAdb13170877	>FR0187049|Putative conserved noncoding region (RNAz) Hsapiens	fRNAdb_v3.4	Ab initio prediction	1.4e-12	100	22	100
	8473740	IDncRNAdb8473740	>gnl|Rnaz|RNZ7987 158	RNAdb_ALL.fasta	Ab initio prediction	1.4e-12	100	22	100
3737 chromosome 11: 130097531 - 130097566	13151363	IDncRNAdb13151363	>FR0124026|Putative conserved noncoding region (RNAz) Hsapiens	fRNAdb_v3.4	Ab initio prediction	1.2e-12	100	31	100
	8473844	IDncRNAdb8473844	>gnl|Rnaz|RNZ8091 114	RNAdb_ALL.fasta	Ab initio prediction	1.2e-12	100	31	100

3802 chromosome 7: 130561507 - 130561543	13132269	IDncRNAdb131 32269	>FR0062169|Putative conserved noncoding region (EvoFold) Hsapiens	fRNAdb_v3.4	Ab initio prediction	7.1e-13	100	58	100
	8193963	IDncRNAdb819 3963	>gnl|evofold|EVF22234 62	RNAdb_ALL.fasta	Ab initio prediction	7.0e-13	100	59	100
4316 chromosome 7: 134955336 - 134955371	13128874	IDncRNAdb131 28874	>FR0051410|Putative conserved noncoding region (RNAz) Hsapiens	fRNAdb_v3.4	Ab initio prediction	1.5e-12	100	18	100
	8463840	IDncRNAdb846 3840	>gnl|Rnaz|RNZ31882 196	RNAdb_ALL.fasta	Ab initio prediction	1.5e-12	100	18	100
4815 chromosome 9: 140066431 - 140066466	13138958	IDncRNAdb131 38958	>FR0084042|Putative conserved noncoding region (RNAz) Hsapiens	fRNAdb_v3.4	Ab initio prediction	1.4e-12	100	23	100
	8467285	IDncRNAdb846 7285	>gnl|Rnaz|RNZ35014 152	RNAdb_ALL.fasta	Ab initio prediction	1.4e-12	100	23	100
5186 chromosome 3: 14494716 - 14494751	13207339	IDncRNAdb132 07339	>FR0305089|Putative conserved noncoding region (RNAz) Hsapiens	fRNAdb_v3.4	Ab initio prediction	1.5e-12	100	18	100
	8453510	IDncRNAdb845 3510	>gnl|Rnaz|RNZ22491 192	RNAdb_ALL.fasta	Ab initio prediction	1.5e-12	100	18	100
5437 chromosome 4: 147574412 - 147574448	13237921	IDncRNAdb132 37921	>FR0403645|Putative conserved noncoding region (EvoFold) Hsapiens	fRNAdb_v3.4	Ab initio prediction	1.5e-12	100	18	100
	8185946	IDncRNAdb818 5946	>gnl|evofold|EVF14946 195	RNAdb_ALL.fasta	Ab initio prediction	1.5e-12	100	18	100
	234161	IDncRNAdb234 161	>CRCNEAC00004778_Human	Condor_ALL_Org_Annota tion.fasta	ncRNA	1.5e-12	100	15	100
5778 chromosome 17: 15166633 - 15166668	13128334	IDncRNAdb131 28334	>FR0049715|Putative conserved noncoding region (RNAz) Hsapiens	fRNAdb_v3.4	Ab initio prediction	1.2e-12	100	32	100
	8445161	IDncRNAdb844 5161	>gnl|Rnaz|RNZ14901 111	RNAdb_ALL.fasta	Ab initio prediction	1.2e-12	100	32	100
5780 chromosome 4: 151678609 - 151678644	13122716	IDncRNAdb131 22716	>FR0031470|Putative conserved noncoding region (RNAz) Hsapiens	fRNAdb_v3.4	Ab initio prediction	1.5e-12	100	19	100
	8457478	IDncRNAdb845 7478	>gnl|Rnaz|RNZ26099 184	RNAdb_ALL.fasta	Ab initio prediction	1.5e-12	100	19	100
654 chromosome 20: 10447092 - 10447127	13156249	IDncRNAdb131 56249	>FR0139412|Putative conserved noncoding region (RNAz) Hsapiens	fRNAdb_v3.4	Ab initio prediction	1.5e-12	100	15	100
	8451918	IDncRNAdb845 1918	>gnl|Rnaz|RNZ21044 236	RNAdb_ALL.fasta	Ab initio prediction	1.5e-12	100	15	100
6540 chromosome 9: 16262515 - 16262550	13232298	IDncRNAdb132 32298	>FR0385391|Putative conserved noncoding region (RNAz) Hsapiens	fRNAdb_v3.4	Ab initio prediction	1.4e-12	100	22	100
	8465790	IDncRNAdb846 5790	>gnl|Rnaz|RNZ33655 160	RNAdb_ALL.fasta	Ab initio prediction	1.4e-12	100	22	100
672 chromosome 14: 104583768 - 104583803	13176984	IDncRNAdb131 76984	>FR0206860|Putative conserved noncoding region (EvoFold) Hsapiens	fRNAdb_v3.4	Ab initio prediction	1.0e-12	100	42	100
	8212128	IDncRNAdb821 2128	>gnl|evofold|EVF38748 84	RNAdb_ALL.fasta	Ab initio prediction	1.0e-12	100	43	100
6962 chromosome 3: 169382202 -	13228695	IDncRNAdb132	>FR0373841|Putative conserved noncoding region	fRNAdb_v3.4	Ab initio	1.3e-12	100	30	100

169382237		28695	(RNAz) Hsapiens		prediction				
	8455591	IDncRNAdb8455591	>gnl|Rnaz|RNZ24383 120	RNAdb_ALL.fasta	Ab initio prediction	1.3e-12	100	30	100
7169 chromosome 9: 17323262 - 17323297	13114567	IDncRNAdb13114567	>FR0005602|Putative conserved noncoding region (EvoFold) Hsapiens	fRNAdb_v3.4	Ab initio prediction	1.5e-12	100	16	100
	13154110	IDncRNAdb13154110	>FR0132588|Putative conserved noncoding region (RNAz) Hsapiens	fRNAdb_v3.4	Ab initio prediction	1.4e-12	100	20	100
	8197844	IDncRNAdb8197844	>gnl|evofold|EVF25762 212	RNAdb_ALL.fasta	Ab initio prediction	1.5e-12	100	17	100
	8465855	IDncRNAdb8465855	>gnl|Rnaz|RNZ33714 172	RNAdb_ALL.fasta	Ab initio prediction	1.4e-12	100	21	100
7337 chromosome 5: 176307457 - 176307492	13171507	IDncRNAdb13171507	>FR0189183|Putative conserved noncoding region (RNAz) Hsapiens	fRNAdb_v3.4	Ab initio prediction	1.6e-12	100	12	100
	8460256	IDncRNAdb8460256	>gnl|Rnaz|RNZ28624 300	RNAdb_ALL.fasta	Ab initio prediction	1.6e-12	100	12	100
7352 chromosome 5: 176561551 - 176561586	13141969	IDncRNAdb13141969	>FR0093641|Putative conserved noncoding region (RNAz) Hsapiens	fRNAdb_v3.4	Ab initio prediction	1.5e-12	100	18	100
	8460260	IDncRNAdb8460260	>gnl|Rnaz|RNZ28628 194	RNAdb_ALL.fasta	Ab initio prediction	1.5e-12	100	18	100
7494 chromosome 3: 17878622 - 17878657	13190798	IDncRNAdb13190798	>FR0251870|Putative conserved noncoding region (EvoFold) Hsapiens	fRNAdb_v3.4	Ab initio prediction	1.4e-12	100	23	100
	8180821	IDncRNAdb8180821	>gnl|evofold|EVF10287 156	RNAdb_ALL.fasta	Ab initio prediction	1.4e-12	100	23	100
79 chromosome 14: 100576031 - 100576066	13206705	IDncRNAdb13206705	>FR0302991|Putative conserved noncoding region (EvoFold) Hsapiens	fRNAdb_v3.4	Ab initio prediction	9.6e-13	100	45	100
	8212030	IDncRNAdb8212030	>gnl|evofold|EVF38659 79	RNAdb_ALL.fasta	Ab initio prediction	9.5e-13	100	46	100
7956 chromosome 3: 186504488 - 186504529	13141670	IDncRNAdb13141670	>FR0092699|Putative conserved noncoding region (RNAz) Hsapiens	fRNAdb_v3.4	Ab initio prediction	1.2e-12	100	34	100
	8455947	IDncRNAdb8455947	>gnl|Rnaz|RNZ24707 105	RNAdb_ALL.fasta	Ab initio prediction	1.2e-12	100	34	100
8070 chromosome 18: 18880113 - 18880148	13175402	IDncRNAdb13175402	>FR0201765|Putative conserved noncoding region (RNAz) Hsapiens	fRNAdb_v3.4	Ab initio prediction	1.3e-12	100	30	100
	8446708	IDncRNAdb8446708	>gnl|Rnaz|RNZ16308 120	RNAdb_ALL.fasta	Ab initio prediction	1.3e-12	100	30	100
8252 chromosome 1: 19253769 - 19253804	13220740	IDncRNAdb13220740	>FR0348120|Putative conserved noncoding region (RNAz) Hsapiens	fRNAdb_v3.4	Ab initio prediction	1.3e-12	100	25	100
	8442838	IDncRNAdb8442838	>gnl|Rnaz|RNZ1279 141	RNAdb_ALL.fasta	Ab initio prediction	1.3e-12	100	25	100
8519 chromosome 1: 198828219 - 198828254	13137204	IDncRNAdb13137204	>FR0078348|Putative conserved noncoding region (EvoFold) Hsapiens	fRNAdb_v3.4	Ab initio prediction	6.4e-13	100	62	100
8519 chromosome 1: 198828219 - 198828254	8218499	IDncRNAdb8218499	>gnl|evofold|EVF4454 58	RNAdb_ALL.fasta	Ab initio prediction	6.2e-13	100	63	100

8975 chromosome 2: 21130849 - 21130884	13187661	IDncRNAdb13187661	>FR0241697|Putative conserved noncoding region (RNAz) Hsapiens	fRNAdb_v3.4	Ab initio prediction	1.6e-12	100	10	100
	8448461	IDncRNAdb8448461	>gnl|Rnaz|RNZ17901 338	RNAdb_ALL.fasta	Ab initio prediction	1.6e-12	100	10	100
9063 chromosome 1: 213898547 - 213898582	13162901	IDncRNAdb13162901	>FR0160866|Putative conserved noncoding region (RNAz) Hsapiens	fRNAdb_v3.4	Ab initio prediction	1.3e-12	100	30	100
	8470016	IDncRNAdb8470016	>gnl|Rnaz|RNZ4263 120	RNAdb_ALL.fasta	Ab initio prediction	1.3e-12	100	30	100
9281 chromosome 22: 22007279 - 22007314	13155521	IDncRNAdb13155521	>FR0137102|Putative conserved noncoding region (EvoFold) Hsapiens	fRNAdb_v3.4	Ab initio prediction	6.8e-13	100	60	100
	8222384	IDncRNAdb8222384	>gnl|evofold|EVF48071 60	RNAdb_ALL.fasta	Ab initio prediction	6.6e-13	100	61	100
9432 chromosome 1: 22441121 - 22441156	13201144	IDncRNAdb13201144	>FR0285131|Putative conserved noncoding region (RNAz) Hsapiens	fRNAdb_v3.4	Ab initio prediction	1.5e-12	100	17	100
	8443399	IDncRNAdb8443399	>gnl|Rnaz|RNZ1330 206	RNAdb_ALL.fasta	Ab initio prediction	1.5e-12	100	17	100
956 chromosome 10: 106504668 - 106504703	13147857	IDncRNAdb13147857	>FR0112745|Putative conserved noncoding region (RNAz) Hsapiens	fRNAdb_v3.4	Ab initio prediction	1.3e-12	100	30	100
	8471574	IDncRNAdb8471574	>gnl|Rnaz|RNZ5821 120	RNAdb_ALL.fasta	Ab initio prediction	1.3e-12	100	30	100
