# Supplementary material for: Small RNAs in metastatic and non-metastatic oral squamous cell carcinoma
Source: BMC Med Genomics. 2015 Jun 24;8:31. doi: 10.1186/s12920-015-0102-4 (PMC4479233; doi:10.1186/s12920-015-0102-4)
Supplement: Additional file 9: — Expression levels of all small RNAs other than miRNA identified in tumor samples. The annotation procedure of this set of small RNAs used BLAST search against available databanks of non-coding RNA sequences but reports showed only evidences from ab initio prediction. [file 12920_2015_102_MOESM9_ESM.pdf]

Additional File 9:

easRNA, pasRNA, rasRNA, nasRNA																		
	**0040**	**0151**	**0291**	**0340**	**0418**	**0486**	**1022**	**1125**	**0012**	**0280**	**0374**	**0397**	**0441**	**0652**	**0677**	**1231**	**1381**	**1642**	**N0/N+**
10517 chromosome X: 26029858 - 26029893	1.9	0	0	0	0	0	0	0	2.97	0	0	0	0	0	0	0	0	0	0.00
10633 chromosome 17: 26662603 - 26662638	0	0	0	0	0	0	0	0	0	0	0	0	0	0	0	0	0	1.48	0.00
10701 chromosome 17: 27050704 - 27050739	0	0	0	0	0	0	0	0	0	0	0	0	0	0	2.12	0	0	0	0.00
11055 chromosome 1: 28833876 - 28833911	0	0	0	0	1.79	0	0	1.85	0	0	0	1.6	0	0	0	0	0	2.81	0.00
11135 chromosome 17: 29298071 - 29298106	0	1.41	0	0	0	0	0	0	0	0	0	0	0	0	0	0	0	0	0.00
11198 chromosome 22: 29729165 - 29729200	0	0	0	0	0	0	0	0	0	0	0	0	0	0	0	0	0	4.89	0.00
11199 chromosome 22: 29729187 - 29729222	0	0	0	0	0	0	0	0	0	0	0	0	0	0	0	0	0	0	0.00
13101 chromosome 19: 3978023 - 3978058	0	0	1.45	0	0	0	0	0	0	0	0	0	0	0	0	0	0	0	0.00
13733 chromosome 7: 43313857 - 43313892	0	12.43	0	0	0	0	0	0	0	0	0	0	0	0	0	0	0	0	0.00
14288 chromosome 21: 46221693 - 46221728	0	2.05	0	0	0	0	0	0	0	0	0	0	0	0	0	0	0	0	0.00
14940 chromosome 19: 49994164 - 49994199	9.7	4.11	43.25	3.27	19.51	0	0	4.04	3.37	28.97	0	9.59	0	0	7.41	10.73	0	10.22	0.00
16801 chromosome 2: 61765303 - 61765338	0	0	0	0	0	0	0	0	0	0	0	2.56	0	0	0	0	0	0	0.00
16935 chromosome 11: 62621145 - 62621180	0	0	0	0	1.97	0	0	0	0	0	0	0	0	0	0	0	0	0	0.00
17156 chromosome 11: 64126641 - 64126676	0	1.41	0	0	0	0	0	0	0	0	0	0	0	0	0	0	0	0	0.00
5101 chromosome 11: 14380686 - 14380721	0	0	1.59	0	0	0	0	0	0	0	0	0	0	0	0	0	0	0	0.00
185 chromosome 3: 101280735 - 101280770	0	0	0	0	0	0	0	0	0	0	0	0	0	0	0	0	0	7.7	0.00
5930 chromosome X: 153657094 - 153657129	3.42	0	0	0	0	0	0	0	3.24	0	10.64	0	0	0	14.81	0	0	0	-6.71
7177 chromosome 17: 1733301 - 1733336	0	0	0	0	0	7.39	0	0	0	4.93	0	0	0	0	0	0	0	0	0.00
7955 chromosome 3: 186504463 - 186504498	0	0	0	0	0	0	0	0	0	2.17	0	0	0	0	0	0	0	0	0.00
7962 chromosome 3: 186505199 - 186505237	0	0	0	0	0	0	0	0	0	0	0	0	0	0	0	0	0	18.66	0.00

asRNA																		
	**0040**	**0151**	**0291**	**0340**	**0418**	**0486**	**1022**	**1125**	**0012**	**0280**	**0374**	**0397**	**0441**	**0652**	**0677**	**1231**	**1381**	**1642**	**N0/N+**
11695 chromosome 10: 3214886 - 3214921	0	0	0	0	0	0	0	7.41	0	0	0	0	0	0	0	0	0	0	0.000
11896 chromosome 20: 33292111 - 33292146	0	0	0	10.57	3.4	0	0	0	0	0	0	0	0	7.72	0	0	0	0	2.717
17551 chromosome 16: 66586389 - 66586424	0	0	0	2.77	0	0	0	0	0	0	0	0	0	0	0	0	0	0	0.000
22537 chromosome 12: 98910173 - 98910208	0	0	0	0	0	0	0	0	0	0	0	0	0	0	0	0	0	4.15	0.000
2282 chromosome X: 117479814 - 117479849	0	0	0	0	0	0	0	0	0	0	0	0	0	0	0	0	0	1.93	0.000
17170 chromosome 14: 64194739 - 64194774	0	0	13.16	0	0	0	2.38	0	0	0	0	0	0	0	0	0	0	0	0.000
7580 chromosome 1: 180123773 - 180123808	0	0	0	0	0	0	0	0	0	0	0	2.88	0	0	0	0	0	0	0.000
**easRNA**																			
11719 chromosome 10: 32298282 - 32298317	**0040**	**0151**	**0291**	**0340**	**0418**	**0486**	**1022**	**1125**	**0012**	**0280**	**0374**	**0397**	**0441**	**0652**	**0677**	**1231**	**1381**	**1642**	**N0/N+**
15645 chromosome 17: 54672051 - 54672087	0	0	0	0	0	0	0	50.05	0	0	0	0	0	0	0	0	0	0	0.000
19996 chromosome 8: 81153691 - 81153726	0	11.89	94.6	0	0	64.32	0	0	3.91	3.55	10.64	5.59	103.29	2.74	9.52	334.95	23.85	0	0.000
4479 chromosome 9: 136629173 - 136629208	0	0	0	0	0	0	0	0	0	0	0	0	0	0	0	0	0	2.22	0.000
5159 chromosome 8: 144660317 - 144660352	0	0	0	0	0	9.37	0	0	0	9.66	0	0	1.8	0	0	0	14.27	0	0.000
570 chromosome 5: 10394191 - 10394227	0	0	0	0	0	0	0	0	0	0	0	0	0	0	0	0	0	5.48	0.000
5741 chromosome 7: 151163616 - 151163652	0	0	2.46	0	2.33	0	0	0	0	0	0	0	0	4.48	0	0	0	0	0.000
7421 chromosome 5: 177631825 - 177631860	0	0	0	0	0	0	0	0	0	0	2.43	0	0	0	7.62	0	0	0	0.000
7799 chromosome 19: 18418328 - 18418363	0	6.27	0	0	0	0	0	0	0	0	0	0	0	0	0	0	0	0	0.000
8117 chromosome 10: 18964393 - 18964428	0	0	0	8.81	0	0	0	1.69	0	0	0	0	0	0	0	0	0	0	0.000
	0	0	0	0	0	0	3.09	0	0	0	0	0	0	0	0	0	0	0	0.000
**rasRNA**																			
	**0040**	**0151**	**0291**	**0340**	**0418**	**0486**	**1022**	**1125**	**0012**	**0280**	**0374**	**0397**	**0441**	**0652**	**0677**	**1231**	**1381**	**1642**	**N0/N+**

10073 chromosome 19: 24185779 - 24185814	0	0	0	0	0	0	0	0	0	0	0	0	0	0	0	0	0	7.7	0.000
1157 chromosome X: 108297800 - 108297835	220.14	0	0	0	0	0	0	0	0	0	0	0	0	0	0	0	0	4.15	79.638
11937 chromosome 17: 33478243 - 33478278	3.62	0	2.31	8.81	12.89	2.16	0	5.39	0	2.17	0	0	0	0	0	0	10.95	1.78	3.545
14495 chromosome X: 47453337 - 47453372	0	1.08	0	0	0	0	0	0	0	0	0	0	0	0	0	0	0	0	0.000
17878 chromosome 6: 68597690 - 68597725	0	0	0	0	0	0	0	0	0	0	0	0	0	0	0	2.44	0	0	0.000
21781 chromosome 13: 93142439 - 93142474	1.9	0	0	0	0	0	0	0	2.16	0	0	0	0	0	0	0	0	0	0.000
3483 chromosome 12: 127650892 - 127650927	0	0	0	0	0	0	0	0	0	2.56	0	0	0	0	0	0	0	0	0.000
5520 chromosome 7: 148638632 - 148638675	0	0	0	0	3.94	0	0	0	0	0	0	0	0	0	0	0	0	1.78	3.323
6196 chromosome 19: 1574499 - 1574534	0	0	0	0	0	0	0	0	1.75	0	0	0	0	0	0	0	0	0	0.000
8094 chromosome 2: 189189558 - 189189593	0	1.41	0	0	0	0	0	0	0	0	0	0	0	0	0	0	0	0	0.000
8493 chromosome 2: 198099548 - 198099583	0	0	0	0	0	0	0	18.37	0	0	0	0	0	0	0	2.68	0	4	4.129
**nasRNA**																			
	**0040**	**0151**	**0291**	**0340**	**0418**	**0486**	**1022**	**1125**	**0012**	**0280**	**0374**	**0397**	**0441**	**0652**	**0677**	**1231**	**1381**	**1642**	**N0/N+**
13687 chromosome 22: 43011319 - 43011354	0	0	0	0	0	0	0	0	0	0	0	3.52	0	0	0	0	0	0	0.00
19109 chromosome 14: 76070569 - 76070604	0	0	0	0	0	0	0	0	0	0	0	0	0	0	0	0	0	2.22	0.00
769 chromosome 11: 10529817 - 10529860	2.09	0	0	0	0	0	0	0	5.67	0	20.72	0	0	0	7.83	0	0	0	-10.91
**SnoRNA**																			
	**0040**	**0151**	**0291**	**0340**	**0418**	**0486**	**1022**	**1125**	**0012**	**0280**	**0374**	**0397**	**0441**	**0652**	**0677**	**1231**	**1381**	**1642**	**N0/N+**
11629 chromosome 6: 31803072 - 31803107	4.38	0	0	0	28.1	0	0	0	0	0	0	0	0	0	0	0	0	0	0.00
1332 chromosome 1: 109643203 - 109643238	0	0	0	0	0	0	0	0	0	0	0	0	0	0	0	0	0	8	0.00
16047 chromosome 8: 56986428 - 56986463	0	0	1.74	0	0	0	0	0	0	0	0	0	0	0	0	0	0	0	0.00
21116 chromosome 20: 8811963 - 8811998	0	0	0	2.52	0	0	0	0	0	0	0	0	0	0	0	0	0	0	0.00
22007 chromosome 9: 95054743 - 95054778	0	0	0	0	0	0	0	0	0	0	0	0	0	0	0	0	0	4.3	0.00

2942 chromosome 11: 122929679 - 122929714	0	0	0	0	0	0	0	2.02	0	0	0	0	0	0	0	0	0	3.11	0.00
3542 chromosome 19: 12817263 - 12817298	0	0	0	0	0	0	0	0	0	0	0	0	0	0	0	0	0	2.22	0.00
7203 chromosome 1: 173833966 - 173834001	0	0	0	0	0	0	0	0	0	0	0	0	0	0	0	0	0	1.63	0.00
**U6 sliceosomal**																			
	**0040**	**0151**	**0291**	**0340**	**0418**	**0486**	**1022**	**1125**	**0012**	**0280**	**0374**	**0397**	**0441**	**0652**	**0677**	**1231**	**1381**	**1642**	**N0/N+**
6114 chromosome 2: 156186951 - 156186986	0.00	0.00	0.00	0.00	0.00	0.00	0.00	0.00	0.00	0.00	0.00	0.00	0.00	0.00	0.00	0.00	0.00	0.00	0.000
15541 chromosome 2: 53797551 - 53797586	0	0	0	0	0	0	0	2.53	0	0	0	0	0	0	0	0	0	0	0.000
20706 chromosome 15: 85481716 - 85481751	0	1.3	0	0	0	0	0	0	0	0	0	0	0	0	0	0	0	0	0.000
5715 chromosome 1: 150995291 - 150995326	0	0	0	0	0	0	0	0	1.35	0	0	0	0	0	0	0	0	0	0.000
9957 chromosome 2: 239321299 - 239321334	0	26.06	0	0	0	6.67	0	0	0	0	0	0	0	0	0	0	0	0	0.000
6405 chromosome 17: 16041148 - 16041183	0	0	0	0	0	0	0	0	0	0	0	0	3.31	0	0	0	0	0	0.000
**Y_RNA**																			
	**0040**	**0151**	**0291**	**0340**	**0418**	**0486**	**1022**	**1125**	**0012**	**0280**	**0374**	**0397**	**0441**	**0652**	**0677**	**1231**	**1381**	**1642**	**N0/N+**
17799 chromosome 16: 68123395 - 68123430	0	0	0	0	0	10.27	0	0	0	0	0	0	0	0	0	0	0	0	0.000
**piRNA**																			
	**0040**	**0151**	**0291**	**0340**	**0418**	**0486**	**1022**	**1125**	**0012**	**0280**	**0374**	**0397**	**0441**	**0652**	**0677**	**1231**	**1381**	**1642**	**N0/N+**
4377 chromosome 18: 13543935 - 13543970	0	0	3.18	0	0	0	0	0	0	0	0	0	0	0	0	0	0	0	0.000
9123 chromosome 18: 21603268 - 21603303	0	0	0	0	0	0	0	0	0	0	0	0	5.39	0	0	0	1.95	0	0.000
**predicted ncRNA**																			
	**0040**	**0151**	**0291**	**0340**	**0418**	**0486**	**1022**	**1125**	**0012**	**0280**	**0374**	**0397**	**0441**	**0652**	**0677**	**1231**	**1381**	**1642**	**N0/N+**

11200 chromosome 22: 29729209 0 29729245	20.74	5.19	1.59	8.81	36.51	0	0	19.72	0	0	0	2.72	2.21	2.49	16.93	12.92	4.3	62.35	0.00
11950 chromosome 18: 33514065 - 33514101	0	0	0	0	0	0	0	0	0	0	0	0	0	0	0	0	0	4.44	0.00
12706 chromosome 21: 37522584 - 37522619	0	1.3	0	0	0	0	0	0	0	0	0	0	0	0	0	0	0	3.11	0.00
12984 chromosome 7: 39019812 - 39019847	0	0	0	0	0	0	0	0	0	0	0	0	0	0	0	0	0	4.74	0.00
13587 chromosome 8: 42396236 - 42396271	0	5.19	0	0	0	0	0	0	0	0	0	0	1.66	0	0	0	0	0	4.69
13855 chromosome 11: 4400339 - 4400375	0	0	5.79	6.04	7.7	0	0	0	0	0	0	0	0	9.71	0	0	0	0	3.02
14794 chromosome 13: 49107193 - 49107228	0	1.19	0	0	0	0	0	0	0	0	0	0	0	0	0	0	0	0	0.00
15395 chromosome 6: 52990602 - 52990637	4	0	0	0	0	0	0	0	0	0	0	0	0	0	0	0	0	0	0.00
15787 chromosome 16: 55513381 - 55513416	2.47	0	0	0	0	0	0	0	2.83	0	0	0	0	0	0	0	0	0	0.00
16223 chromosome 12: 58007376 - 58007411	0	0	0	0	0	2.88	0	0	0	0	0	0	4	0	0	0	3.13	0	0.00
16578 chromosome 15: 60334099 - 60334134	0	0	0	0	0	0	0	0	0	0	0	0	1.38	0	0	0	0	0	0.00
16757 chromosome 11: 61404307 - 61404342	0	0	0	0	0	0	0	0	3.91	0	0	0	0	0	0	0	0	0	0.00
16793 chromosome 11: 61723233 - 61723268	0	0	0	0	0	0	0	0	0	0	0	0	0	0	0	0	2.93	0	0.00
17317 chromosome 8: 65182995 - 65183031	0	13.41	0	0	0	15.49	0	0	1.35	20.7	0	0	106.88	0	0	0	33.03	0	-3.73
17344 chromosome 17: 65373515 - 65373550	0	0	0	0	0	0	0	0	0	0	2.24	0	0	0	0	0	0	0	0.00
19764 chromosome 17: 79669775 - 79669810	0	0	7.38	0	2.15	0	0	3.2	0	0	0	0	0	0	0	0	0	0	0.00
203 chromosome 10: 101380916 - 101380951	0	0	0	6.04	2.86	0	0	2.02	0	0	0	0	0	0	0	0	0	0	0.00
20828 chromosome 15: 86291864 - 86291899	0	2.16	0	0	0	0	0	0	0	0	0	0	1.66	0	0	0	0	0	0.00
21622 chromosome 16: 9185324 - 9185359	0	0	0	0	0	0	0	0	0	0	0	0	0	0	0	0	0	2.22	0.00
21791 chromosome 5: 93334408 - 93334443	0	0	0	0	0	0	6.42	0	0	0	0	9.43	0	0	0	3.9	0	7.85	-2.20
3642 chromosome 9: 129171091 - 129171126	0	0	0	0	0	0	0	0	1.48	0	0	0	0	0	0	0	0	0	0.00
5210 chromosome 8: 145133507 - 145133542	0	1.3	0	0	0	0	0	0	0	0	0	0	4.42	0	0	0	0	0	-2.26
5549 chromosome 7: 148910945 - 148910982	6.47	0	0	0	0	0	0	0	12.01	0	25.94	0	0	0	20.1	0	0	0	-5.98

6016 chromosome 19: 15490319 - 15490354	0	0	1.45	0	0	0	0	2.19	0	0	0	0	0	0	0	0	0	0	0.00
630 chromosome 12: 104324223 - 104324258	2.09	2.6	0	0	0	0	0	1.85	3.64	0	0	0	2.9	0	0	0	0	1.78	0.00
7378 chromosome 2: 176972944 - 176972979	2.09	0	0	0	0	0	0	0	5.67	0	20.72	0	0	0	7.83	0	0	0	-10.91
8010 chromosome 5: 1875043 - 1875078	0	0	0	0	0	0	0	0	0	2.37	0	0	2.21	0	0	0	0	0	0.00
8130 chromosome 21: 18985777 - 18985812	0	0	0	0	0	0	0	0	1.62	0	0	0	0	0	0	0	0	0	0.00
8154 chromosome 22: 19032147 - 19032182	0	1.08	0	0	0	0	0	0	0	0	0	0	0	0	0	0	0	0	0.00
8571 chromosome 17: 2004898 - 2004933	0	0	0	0	0	0	0	0	0	0	0	0	0	2.49	0	0	0	0	0.00
8621 chromosome 11: 2017798 - 2017833	0	1.08	0	0	0	0	0	0	0	0	0	0	0	0	0	0	0	0	0.00
9181 chromosome 2: 217736642 - 217736677	0	0	0	0	0	0	0	6.57	0	0	0	0	0	0	0	0	0	0	0.00
980 chromosome 8: 106686400 - 106686435	0	0	0	0	0	0	0	0	0	0	0	0	0	0	0	0	3.52	0	0.00
9835 chromosome 19: 23558048 - 23558083	0	1.95	0	0	0	0	0	0	0	0	0	0	0	0	0	0	0	0	0.00
**Ab initio prediction**																			
	**0040**	**0151**	**0291**	**0340**	**0418**	**0486**	**1022**	**1125**	**0012**	**0280**	**0374**	**0397**	**0441**	**0652**	**0677**	**1231**	**1381**	**1642**	**N0/N +**
10074 chromosome 19: 24185867 - 24185902	0	0	0	0	0	0	0	5.22	0	0	0	0	0	0	0	0	0	0	0.00
10396 chromosome 2: 25454991 - 25455026	0	1.19	0	0	0	0	0	0	0	0	0	0	0	0	0	0	0	0	0.00
10567 chromosome 1: 26253801 - 26253836	0	0	0	0	0	0	0	6.07	0	0	0	0	0	0	0	0	0	0	0.00
10872 chromosome 1: 27929061 - 27929096	0	0	6.08	0	0	0	0	0	0	0	0	0	0	0	0	0	0	0	0.00
10983 chromosome 3: 2843082 - 2843117	0	0	0	0	0	0	0	0	0	0	0	0	0	0	0	0	2.74	0	0.00
11420 chromosome 19: 30832066 - 30832101	0	0	0	0	0	0	0	0	0	0	0	0	0	0	0	0	0	2.07	0.00
11425 chromosome 19: 30860748 - 30860783	0	0	0	0	0	0	4.75	0	0	0	0	0	0	0	0	0	0	0	0.00
11881 chromosome 6: 33175618 - 33175653	0	1.19	0	0	2.51	0	0	3.71	0	0	0	0	1.8	0	0	7.56	0	6.96	0.00
11923 chromosome 6: 33419940 - 33419975	13.51	0	0	0	0	1.98	0	0	26.05	0	14.18	0	4.56	0	25.81	0	5.86	0	-3.29
12006 chromosome 17: 33884684 - 33884719	0	0	0	0	0	0	0	0	0	0	0	0	0	2.49	0	0	0	0	0.00
12677 chromosome 15: 37388498 - 37388533	0	3.14	0	0	0	0	0	0	0	0	0	0	0	0	0	0	0	0	0.00

12750 chromosome 6: 37693032 - 37693067	0	5.73	0	0	0	0	0	0	0	0	0	0	4	0	0	0	0	0	2.15
1283 chromosome X: 109298576 - 109298611	0	3.78	0	0	0	0	0	0	0	0	0	0	0	0	0	0	0	0	0.00
12947 chromosome 20: 38792900 - 38792935	0	3.78	0	0	0	0	0	0	0	0	0	0	0	0	0	0	0	0	0.00
13390 chromosome 17: 41215797 - 41215832	0	0	2.6	0	0	0	0	0	0	0	0	0	0	0	0	0	0	0	0.00
13566 chromosome 2: 42255151 - 42255186	0	3.14	0	0	0	0	0	0	0	0	0	0	0	0	0	0	0	0	0.00
1402 chromosome X: 110187501 - 110187536	0	0	0	0	0	2.7	0	0	2.16	2.56	1.87	3.52	0	0	0	0	4.5	0	-3.60
14322 chromosome 19: 46440705 - 46440740	0	0	0	0	0	0	0	0	0	0	0	0	2.35	0	0	0	0	0	0.00
14548 chromosome 1: 47697993 - 47698028	0	0	0	0	0	1.98	0	0	0	0	0	0	1.38	0	0	0	0	0	2.15
14693 chromosome 18: 4847267 - 4847302	0	0	13.02	18.37	3.4	0	0	0	0	0	0	0	0	21.17	0	0	3.13	0	2.15
14743 chromosome X: 48815192 - 48815227	0	2.05	0	0	0	2.88	0	0	0	2.76	0	0	12.29	0	0	0	7.82	0	-3.09
15925 chromosome 17: 56408644 - 56408679	0	0	2.89	0	0	0	0	0	0	0	0	0	0	0	0	0	0	0	0.00
16106 chromosome 14: 57321252 - 57321287	0	0	0	0	0	0	0	0	1.75	0	0	0	0	0	0	0	0	0	0.00
16118 chromosome 11: 57408685 - 57408720	5.71	2.05	0	6.04	8.95	0	0	2.86	0	0	0	19.81	0	2.74	0	0	0	0	0.00
16207 chromosome 17: 57918633 - 57918668	0	0	5.35	0	0	0	0	0	0	0	0	0	0	0	0	0	0	0	0.00
1708 chromosome 8: 112453349 - 112453386	17.5	0	0	0	0	0	0	0	28.34	0	30.61	0	0	0	0	0	0	0	-2.24
17625 chromosome 11: 67134690 - 67134725	0	0	0	0	0	0	0	0	0	0	0	0	0	0	0	0	0	3.55	0.00
18109 chromosome 7: 70038210 - 70038245	0	0	0	0	0	0	0	0	0	0	2.99	0	0	0	0	0	0	0	0.00
18122 chromosome 3: 70125795 - 70125831	0	1.19	0	0	0	0	0	0	0	0	0	0	5.8	0	0	0	0	0	-3.25
18157 chromosome 15: 70391562 - 70391597	11.23	0	0	0	0	0	0	0	25.37	0	0	0	0	0	0	0	0	0	0.00
18160 chromosome 17: 704270 - 704306	0	0	3.62	4.03	0	0	0	0	0	0	0	0	0	2.49	0	0	0	0	4.61
18336 chromosome 3: 71591156 - 71591193	35.96	44.44	52.65	0	32.39	3.96	3.57	11.12	43.05	68	8.03	1.76	39.77	3.74	4.23	0	2.54	15.85	0.00
18442 chromosome 3: 72221386 - 72221422	0	0	0	0	0	0	0	0	0	0	0	2.56	0	0	0	0	0	36.43	0.00
18526 chromosome 3: 72817283 - 72817318	0	0	0	0	1.97	0	0	0	0	0	0	0	0	0	0	0	0	0	0.00

18665 chromosome 2: 7350334 - 7350369	2.85	19.57	0	0	1.97	1.8	0	0	2.29	1.97	0	0	30.93	0	0	0	0	0	0.00
18716 chromosome X: 73834571 - 73834606	0	0	3.91	0	0	0	4.52	0	0	0	0	8.47	0	0	0	0	0	0	0.00
19479 chromosome 15: 78031473 - 78031508	0	0	0	0	0	0	0	0	0	0	0	0	0	0	0	0	2.15	0	0.00
20187 chromosome 18: 8218318 - 8218353	0	0	0	0	0	0	0	0	0	0	0	0	1.8	0	0	0	5.28	0	0.00
20303 chromosome 5: 82968713 - 82968748	0	0	0	0	0	0	0	3.03	0	0	0	0	0	0	0	0	0	0	0.00
20488 chromosome 7: 8418219 - 8418254	0	0	0	0	0	0	0	0	0	0	0	0	0	0	0	0	0	13.48	0.00
20805 chromosome 10: 86151459 - 86151494	0	0	1.59	0	0	0	0	0	0	0	0	0	0	0	0	0	0	0	0.00
210 chromosome 17: 1014140 - 1014175	0	0	0	0	0	0	0	0	0	0	0	0	0	0	0	0	0	1.48	0.00
2116 chromosome 5: 115962268 - 115962303	0	0	10.56	0	5.19	0	0	6.91	0	0	0	0	0	0	0	0	0	0	0.00
21163 chromosome 1: 88450683 - 88450718	2.66	0	0	0	0	0	0	0	0	0	0	0	0	0	0	0	0	0	0.00
21184 chromosome 5: 88586421 - 88586456	0	0	0	0	0	1.98	0	0	0	2.17	0	0	0	0	0	0	0	0	0.00
22243 chromosome 15: 96949661 - 96949696	0	0	1.74	0	0	0	0	0	0	0	0	0	0	0	0	0	0	0	0.00
22320 chromosome 12: 97503032 - 97503067	0	0	0	0	0	0	0	0	0	0	0	0	0	0	0	0	0	1.48	0.00
22595 chromosome 8: 99395031 - 99395066	0	0	0	0	0	0	3.09	0	0	0	0	0	0	0	0	0	0	0	0.00
2436 chromosome X: 118827625 - 118827660	0	0	5.5	6.04	2.68	0	0	4.89	0	0	0	0	0	5.73	0	0	0	0	5.01
2640 chromosome 1: 120396276 - 120396311	0	0	0	0	0	0	0	0	6.07	0	0	0	0	0	0	0	0	0	0.00
2796 chromosome 4: 121771413 - 121771448	0	0	0	0	0	0	0	0	0	0	0	0	0	0	2.54	0	0	0	0.00
2877 chromosome 5: 122435527 - 122435562	0	0	0	0	0	0	0	0	3.1	0	0	0	0	0	0	0	0	0	0.00
309 chromosome 10: 102194923 - 102194958	0	0	0	0	0	0	0	0	0	0	0	0	0	0	11.85	0	0	0	0.00
3313 chromosome 11: 126148420 - 126148455	0	1.3	0	0	0	0	0	0	0	0	0	0	0	0	0	0	0	0	0.00
3737 chromosome 11: 130097531 - 130097566	0	1.08	0	0	0	0	0	0	0	0	0	0	0	0	0	0	0	0	0.00
3802 chromosome 7: 130561507 - 130561543	0	0	0	7.3	0	0	0	0	0	0	0	0	0	0	0	0	0	0	0.00
4316 chromosome 7: 134955336 - 134955371	0	0	0	0	0	0	0	0	1.48	0	0	2.72	0	0	0	0	0	0	0.00

4815 chromosome 9: 140066431 - 140066466	3.42	0	0	0	0	0	0	0	11.3 4	0	0	0	0	0	0	0	0	0	-2.21
5186 chromosome 3: 14494716 - 14494751	0	0	1.74	0	0	0	0	2.02	0	0	0	0	0	2.49	0	0	0	0	2.27
5437 chromosome 4: 147574412 - 147574448	0	0	24.3	0	11.8 1	0	0	0	5.4	0	0	0	0	0	0	0	0	2.37	6.98
5778 chromosome 17: 15166633 - 15166668	0	0	0	0	0	2.7	0	0	1.75	0	0	0	0	0	0	0	0	0	2.32
5780 chromosome 4: 151678609 - 151678644	0	1.08	0	0	0	0	0	0	0	0	0	0	3.59	0	0	0	2.15	0	-3.54
654 chromosome 20: 10447092 - 10447127	0	0	0	0	0	0	0	0	2.29	0	0	0	0	0	0	0	0	0	0.00
6540 chromosome 9: 16262515 - 16262550	0	0	0	0	0	0	0	0	1.89	0	0	0	0	0	0	0	0	0	0.00
672 chromosome 14: 104583768 - 104583803	0	0	0	0	0	2.16	0	0	0	0	0	0	2.76	0	0	0	0	0	0.00
6962 chromosome 3: 169382202 - 169382237	0	1.19	0	0	0	0	0	0	3.37	0	0	0	0	0	0	0	0	1.78	-2.88
7169 chromosome 9: 17323262 - 17323297	0	0	0	0	0	0	7.85	0	0	0	0	0	0	0	0	0	0	0	0.00
7337 chromosome 5: 176307457 - 176307492	0	0	7.09	0	3.22	0	0	0	0	0	0	0	0	0	0	0	0	0	0.00
7352 chromosome 5: 176561551 - 176561586	4.76	0	0	0	0	0	0	0	4.99	0	0	0	0	0	0	0	0	7.55	0.00
7494 chromosome 3: 17878622 - 17878657	0	0	0	0	0	0	4.52	0	0	0	0	4.47	0	0	0	0	0	0	0.00
79 chromosome 14: 100576031 - 100576066	0	0	0	15.1	0	0	0	0	0	0	0	0	0	0	0	0	0	0	0.00
7956 chromosome 3: 186504488 - 186504529	0	0	0	0	0	0	0	4.55	0	0	0	0	0	0	0	0	0	9.92	0.00
8070 chromosome 18: 18880113 - 18880148	0	0	1.88	0	0	0	0	0	0	0	0	0	0	0	0	0	0	0	0.00
8252 chromosome 1: 19253769 - 19253804	0	1.95	0	0	0	0	0	0	0	0	0	0	0	0	0	0	0	0	0.00
8519 chromosome 1: 198828219 - 198828254	11.23	16.5 4	11.2 8	0	7.7	0	0	8.09	6.34	0	2.05	0	18.2 3	0	0	0	0	3.41	2.74
8975 chromosome 2: 21130849 - 21130884	0	0	0	0	0	0	0	0	0	0	0	0	0	0	0	0	0	1.78	0.00
9063 chromosome 1: 213898547 - 213898582	0	0	0	0	0	0	0	0	0	0	0	3.36	0	0	0	0	0	0	0.00
9281 chromosome 22: 22007279 - 22007314	0	0	0	0	0	0	0	0	0	0	0	1.6	0	0	0	0	0	0	0.00
9432 chromosome 1: 22441121 - 22441156	2.66	0	0	0	0	0	0	65.0 4	0	0	0	0	0	0	0	0	0	0	0.00
956 chromosome 10: 106504668 - 106504703	0	3.46	0	0	0	0	0	0	0	0	0	0	0	0	0	0	0	0	0.00
